# Proof of Concept of an Eclectic, Integrative Therapeutic Approach to Mental Health and Well-Being Through Virtual Reality Technology

**DOI:** 10.3389/fpsyg.2020.00858

**Published:** 2020-06-05

**Authors:** Paul Frewen, Divya Mistry, Jenney Zhu, Talia Kielt, Christine Wekerle, Ruth A. Lanius, Rakesh Jetly

**Affiliations:** ^1^University of Western Ontario, London, ON, Canada; ^2^McMaster University, Hamilton, ON, Canada; ^3^Canadian Armed Forces, Ottawa, ON, Canada; ^4^University of Ottawa, Ottawa, ON, Canada

**Keywords:** virtual reality, integrative psychotherapy, posttraumatic stress disorder (PTSD), dissociative experiences, wellbeing, positive affect (PA)

## Abstract

Across three studies, we provide a proof-of-concept evaluation of an integrative psychotherapeutic application of virtual reality (VR) technology. Study 1 (*n* = 36) evaluated an unguided “safe-place” imagery task, where participants were instructed “to create a safe space… [such as] a scene, item, design, or any visual representation that makes you feel safe” using either the Google Tilt Brush application (VR condition), the standard Microsoft Paint application (2-D condition), or via eyes-closed mental imagery alone (IMG condition). Study 2 (*n* = 48) evaluated a narrative episodic recall task, where participants viewed their childhood and adult homes and places of schooling either using either the Google Earth VR application (VR condition) or the standard Google Earth application (2-D condition) or recalled these places with their eyes closed via mental imagery alone (IMG condition). Finally, Study 3 (*n* = 48) evaluated a guided wilderness imagery task, during which different scripts were narrated, specifically, a trail walk in autumn, a spring meadow, and a hillside walk in snowy winter, while either these same scenes were visually presented using the Nature Treks VR application (VR condition), the scenes were presented using the same software but shown on standard computer monitor (2-D condition), or participants’ eyes were closed (IMG condition). Order of intervention format was randomized across participants. Across all three studies, quantitative survey ratings showed that the VR format of intervention delivery produced greater positive affect and satisfaction and perceived credibility ratings as an intervention for trauma- and stressor-related disorders and psychological well-being as rated by university students who varied in traumatic and stressful life event history and symptoms of posttraumatic stress disorder, whereas qualitative findings revealed additional themes of experiential response including increased experience of presence and vividness in the VR condition. Future research directions and clinical applications are discussed.

## Proof of Concept of Virtual Reality Integrative Therapy for Mental Health and Well-Being

Whereas computer graphical environments are usually depicted on a two-dimensional (2-D) screen such as a standard monitor, virtual reality (VR) can be defined as “a computer-generated simulation of a lifelike environment that can be interacted with in a seemingly real or physical way by a person, especially by means of responsive hardware such as a visor with screen,” that is, using a head-mounted display (HMD) in place of a standard monitor (Virtual Reality, 2019). Depicting graphical stimuli in this way can increase their perceived vividness and the extent to which the user experiences a sense of “presence,” that is, that the user is “in” the computer graphical environment.

Current articles in popular scientific media tout the potential of today’s VR technology for improving mental health and well-being (Temming, 2018; Weir, 2018; Martin, 2019); however, the peer-reviewed clinical psychology literature investigating VR applications to mental health is only in a nascent stage. For example, referring specifically to treatment for trauma and stressor-related disorders, clinical research published to date is almost exclusively limited to the evaluation of VR applications for exposure therapy (VRET), a treatment based on the principles of extinguishing conditioned fears by repeatedly habituating participants to stimuli reminiscent of their traumatic memories (Foa and Kozak, 1986). A recent systematic review and meta-analysis of 18 studies including a total 759 participants demonstrated that VRET reduced posttraumatic stress disorder (PTSD) and depressive symptoms compared to inactive control groups (e.g., waitlist) with a moderate effect size, but no significant differences were found when comparing VRET with active control groups (e.g., non-VR exposure therapy) (Deng et al., 2019). Moreover, effect sizes were seen to correlate positively with increasing number of sessions and to be maintained at 3- and 6-month follow-ups (Deng et al., 2019). One can conclude from these results that VRET is more effective than no treatment, but not more effective than non-VR exposure therapy. These results agree with an earlier systematic review suggesting significantly better PTSD outcomes for VRET when compared to inactive (waitlist) control, but only equivalent efficacy in comparison with active controls (e.g., traditional imaginal and *in vivo* exposure therapy) (Gonçalves et al., 2012). Such results are also broadly consistent with findings observed for VRET for other anxiety disorders (e.g., Opriş et al., 2012).

However, from the perspective of the psychotherapy integration movement, exposure represents only one of many different psychological interventions that could bring about recovery from trauma and stressor-related disorders, and it is puzzling that so little research has evaluated outcomes for other structured psychological interventions when administered within VR. In fact, writing more than 15 years earlier, one pioneering VR psychologist had recommended that “because VR could be part of the future of clinical psychology, it is critical to all psychotherapists that it should be defined broadly” (Riva, 2005, p. 220), describing several different applications for VR informed by the different schools of psychotherapy. Specifically, Riva recommended that whereas “behavioral therapists may use a virtual environment for activating the fear structure in a phobic patient through confrontation with the feared stimuli” (pp. 225–226), consistent with the aforementioned concept of VRET, he further considered that “a cognitive therapist may use VR situations to assess situational memories or disrupt habitual patterns of selective attention; experiential therapists may use VR to isolate the patient from the external world and help him or her in practicing the right actions; [and] psychodynamic therapists may use virtual environments as complex symbolic systems for evoking and releasing affect” (pp. 225–226).

Consistent with the broad application of VR for clinical psychology advanced by Riva (2005), here we provide a proof-of-concept, single-session evaluation of a psychotherapy integration approach to the application of VR technology to improve mental health and well-being that we will term *virtual reality integrative therapy* (VRIT). One popular way to define psychotherapy integration is by emphasizing “common factors” (e.g., therapeutic alliance, empathy) rather than specific psychotherapy techniques or tasks (e.g., unguided or guided imagery, episodic recall) as supposed primary mediators of psychotherapy outcomes (Zarbo et al., 2016). However, the alternative principle of “technical eclecticism” defines the scope and practice of psychotherapy integration differently (Zarbo et al., 2016). Specifically, technical eclecticism involves administering specific psychotherapeutic interventions developed within various schools of psychotherapy without rigid adherence to the theoretical principles of any specific school (e.g., behavioral, cognitive-behavioral, interpersonal, psychodynamic) or even the need for understanding of the psychological mechanisms of change underlying therapeutic effects (Zarbo et al., 2016). Consistent with the technical eclecticism principle and considering psychotherapy as the provision of a menu of structured psychological interventions tailored to individual patient needs and preferences, here we administer three common psychotherapeutic tasks in each of three formats to evaluate the impact of delivery format on immediate affective responses and perceived satisfaction and credibility of the tasks as interventions for trauma- and stressor-related disorders and improving mental health and well-being more generally. Specifically, we administered each intervention: (1) in the traditional noncomputerized psychotherapy format as generally relying on mental imagery and episodic recall rather than stimulus perception while participants’ eyes were closed [imagery condition (IMG)]; (2) in the traditional computerized format involving depicting visual stimuli in two dimensions via standard flat-screen (e.g., laptop) monitor (2-D perception condition); and (3) in the computerized format of VR involving depicting stimuli in three-dimensional format through the use of an HMD.

**FIGURE 1:**
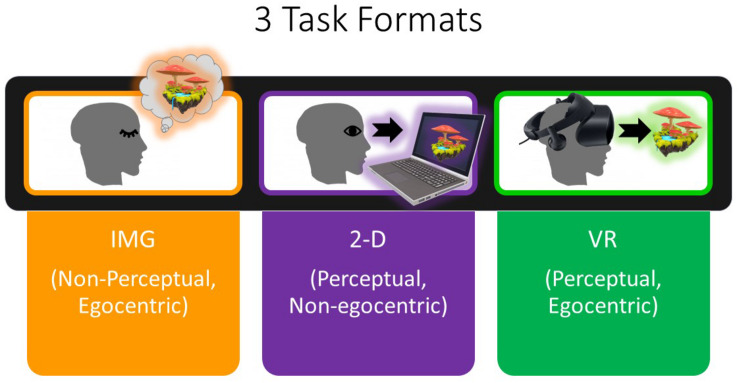
Completion of the IMG (noncomputerized) format of each task is thought to be mediated by eyes-closed imagery and/or episodic recall rather than perception and therefore subjectively experienced with lower vividness at least among certain participants. Nevertheless, participants were expected to typically experience the IMG format of each task from an egocentric frame of reference or sense of “presence” during the task. In comparison, in the 2-D format, participants were expected to experience each task less from the egocentric point of view and rather more so from a third-person perspective; as a result, the 2-D format of the task was thought to be experienced with a lower level of presence. Finally, the VR format of each task, by comparison to the others, was expected to be experienced with high vividness of perception and high egocentricity (presence). IMG, imagery; 2-D, two-dimensional; VR, virtual reality.

Further, although the eclectic approach to psychotherapy is generally described as disinterested in the study of psychological mechanisms of change but rather only in the practical clinical effectiveness of different interventions (e.g., Zarbo et al., 2016), the present work is interested to initiate the study of common psychological mechanisms of change mediating the outcomes of these different VR versus non-VR intervention formats. One means to conceptualize these differing treatment delivery formats is to consider that the traditional noncomputerized intervention format, typically conducted with participants’ eyes closed, will be mediated primarily through the use of memory and/or mental imagery, the latter defined as “the experience of perception in the absence of external sensory input” (e.g., Ji et al., 2019). In comparison, the computerized modes of treatment delivery will be rendered through direct perception of visual (and auditory) stimuli when conducted with eyes open. Although Ji et al. review studies showing that mental imagery often evokes strong emotional responses in comparison with verbal stimuli; neuroimaging studies provide a theoretical basis for considering mental imagery only as a form of “weak perception,” that is, capable of evoking response in similar neural networks to direct perception but typically only at a comparably lower level (Pearson and Kosslyn, 2015; Pearson et al., 2015). As such, we develop the hypothesis that direct perception of stimuli as conducted in the realistic, immersive medium of VR should evoke stronger emotional response when compared with mental imagery and/or episodic recall as is typically conducted in traditional psychotherapy settings. This hypothesis is further in keeping with Riva (2005, p. 226) definition of VR “as an advanced imaginal system: an experiential form of imagery that is as effective as reality in inducing emotional responses.” Moreover, VR is known to engender greater feelings of “presence” within the computer graphically depicted environments when compared to presenting the same stimuli on standard flat-screen (e.g., laptop) monitors (e.g., Lessiter et al., 2001). As such, we conceived that the IMG condition would typically involve the naturalistic egocentric frame of reference but did not involve direct perception of visual stimuli while participants’ eyes were closed. In comparison, we considered that the 2-D condition would involve direct visual perception but from a third-person point of view. Finally, we regarded the VR condition as involving direct visual perception from the point of view of egocentricity (Figure 1).

Regarding choice of psychotherapeutic tasks, here we examine affective response to an unguided “safe place” imagery task (Study 1), to a structured “narrative” task involving location-based autobiographical recall (Study 2), and to a guided (script-driven) imagery task referring to natural wilderness environments (Study 3). These tasks were selected based on the eclectic perspective that they are each frequently used in the conduct of psychotherapy irrespective of any broader, integrative theory that might explain their clinical effectiveness. The order in which the three formats of each of these three tasks was administered was randomized across participants in a within-subjects design utilizing a common methodology involving collection of open-ended (qualitative) experiential responses to each format immediately following each task and comparative questionnaire (quantitative) ratings following the completion of all three formats of the task.

The present study recruited university students to participate in this initial proof-of-concept study (Study 1, *n* = 36; Study 2, *n* = 48; Study 3, *n* = 48). Besides acting as a sample of convenience, university students were of interest to recruit given past literature suggesting that, as a group, college-attending young adults are frequently exposed to traumatic and nontraumatic stressors and experience a high degree of PTSD symptoms, including an increased risk for exposure to certain traumatic stressors such as sexual assault, compared to the general population (Avant et al., 2011; Read et al., 2011; Johnson and Johnson, 2013). Furthermore, university represents a period marked by adjustment to challenges in various life domains including social and psychological challenges (Credé and Niehorster, 2012) and is a period of time in which mental health concerns often emerge (Hunt and Eisenberg, 2010). As such, exploration of affective responses to psychotherapeutic tasks among a sample of university students may inform current understanding of trauma- and stressor-related disorders and psychological well-being within this population. The following section describes the general methodology used in all three studies, which is followed by a description of the rationale, specific methods and results unique to each study. This report concludes with a general discussion regarding the collective results of all three studies.

## General Methods

### Research Procedures Across All Three Studies

This research involving human participants was reviewed and approved by the health sciences research ethics board of Western University, Canada. All participants provided informed consent prior to participation. The authors have no potential conflicts of interest to disclose. The authors also note that no adverse events occurred throughout the conduct of this research.

Individuals were invited to participate in the present three studies by viewing an advertisement presented on the university’s psychology department research participation website. Interested participants met individually with the experimenter at a designated location on the university’s campus and received partial credit toward requirements for an introductory psychology course in which they were enrolled. It was explained that participants were permitted to leave the study at any point without penalization.

Participants were asked to complete self-report questionnaires that assessed their degree of exposure to stressful and traumatic life events [Life Events Survey (LES; Sarason et al., 1978), Life Events Checklist for *DSM-5* (LEC-5; Weathers et al., 2013), Adverse Childhood Experiences (ACE; Felitti et al., 1998)], or measured psychological symptoms associated with PTSD [Posttraumatic Stress Disorder Checklist for *DSM-5* (PCL-5; Blevins et al., 2015)], dissociative experiences [Trauma-Related Altered States of Consciousness (TRASC; Frewen and Lanius, 2014; Frewen et al., 2016)], and depression and anxiety [Patient Health Questionnaire 4 (Kroenke et al., 2009)]. Further description of each of these psychometrically validated questionnaires can be found in the Supplementary Material.

Participants were randomized to either complete the VR format of the exercises first, second, or third in comparison to completing the exercises using an analogous 2-D display (laptop screen, 2-D format) or via eyes-closed mental imagery/recall alone (IMG format). In other words, all participants completed the exercises in all three formats, a VR format and two non-VR (2-D and IMG) formats, during the same experimental session, in counterbalanced random order. Concerning the VR administration, a Samsung Odyssey+ Windows Mixed Reality HMD was used along with the associated hand controllers. This HMD has a 110° field of view via a dual 3.5″ AMOLED display providing 1,440 × 1,600 pixel resolution up to 90 Hz and includes integrated headphones facilitating AKG 360° spatial sound^1^. All software applications were powered by a Microsoft Windows 10 computer and the Windows Mixed Reality functionality. All applications were also rated as being appropriate for use by “EVERYONE” by the international Entertainment Software Rating Board, indicating that the “content is generally suitable for all ages”^2^.

After completing each exercise separately in each format, the researcher interviewed participants about their phenomenological response to the exercise: “What was that like? How did you feel? What did you experience?” Participants’ qualitative responses to these questions were recorded verbatim and transcribed. After completing all three formats of the exercise, participants also completed a satisfaction and credibility survey as used in Frewen et al. (2015) and the Modified Differential Emotions Scale (mDES; Fredrickson et al., 2003) comparatively, that is, providing responses to each of the three exercises separately in the order in which they were completed. Detailed description of these validated surveys can also be found in the Supplementary Material.

### Research Design and Analysis Across All Three Studies

The research design for each study involved one within-group repeated-measures independent variable, namely, the FORMAT in which each psychotherapeutic exercise was delivered (i.e., IMG versus 2-D versus VR; Figure 1). In other words, all participants completed all three formats of the task. The study also included one between-group independent variable, referring to which of a possible six different counterbalanced random ORDERs that different participants completed the exercises in, although for the purposes of statistical analysis this grouping factor will be simplified to describing only whether the VR FORMAT was completed first (i.e., regardless of the ORDER in which the remaining two tasks were completed). As we were interested in specific emotional responses and satisfaction–credibility ratings to each of the three tasks in each of the three formats, statistical analysis of questionnaire responses was undertaken at the item level using multivariate approaches to control for multiple comparisons [i.e., split-plot multivariate analysis of variance (MANOVA) with Greenhouse–Geisser correction]. Two-tailed statistical significance was determined at *p* < 0.05 for multivariate effects and follow-up univariate effects. See the Supplementary Material for detailed results of all main effects and interactions for the MANOVA and follow-up analysis of variance (ANOVA) and paired *t* tests including obtained effect sizes and power analysis; by contrast, only statistically significant results are reported herein. We also evaluated whether baseline symptoms of PTSD (PCL-5 scores) predicted negative emotional responses on the mDES to each FORMAT of the task in the form of Pearson *r*. However, we emphasize that participants were not randomized to the three tasks [i.e., guided “safe place” imagery, narrative “life-review” exercise, and guided (scripted) imagery of natural wilderness environments], but rather these tasks were administered to different participants in three consecutive studies described separately in the foregoing.

Finally, in this mixed-methods study, participant responses to the open-ended questions were assessed using thematic analysis. Specifically, two student researchers independently identified codes summarizing participant responses to the open-ended questions into themes. These themes were subsequently compared to identify overlapping themes and a final set of agreed-upon codes in consultation with the principal investigator, the supervising psychologist. The student researchers then individually coded each participant response for the presence of the final set of codes, and instances of disagreement were broken by the supervising psychologist. Further, as this program of research was conducted consecutively over the course of three studies, we added to the code list used in Study 1 during Study 2, and to the list used in Study 2 during Study 3. However, only those codes that characterized a minimum 10% of participants’ comments are described individually in this report, generally using one to three verbatim examples. Further, making use of the entire set of codes, we identified the mean number of codes identified in the comments collected across participants, as well as the standard deviation, and report in full the comments made by participants whose self-reported responses included a number of codes that was two standard deviations above the mean, thereby indicative of particularly qualitatively rich responses. Only codes descriptive of responses to the VR format of the tasks are described herein.

## Study 1: Unguided Imagery

Providing instruction in negative emotion regulation skills including so-called “self-soothing techniques” (e.g., Wright, 2009) is frequently undertaken in the context of psychological treatment for PTSD including as a precursor to traumatic memory-focused therapy (e.g., Cloitre et al., 2004). One such approach includes utilizing mental imagery to create a “safe space,” often used both as a “warm-up” to psychotherapy sessions and as a “sanctuary” following therapy sessions (e.g., Jubb, 2017). Within the context of treatment for PTSD, imagining the safe space could involve visualizing pleasant scenes or memories to protect from distressing experiences and intrusive traumatic memories.

Although most psychotherapists administer the safe space exercise as a mental imagery task, art therapy represents an alternative, behavioral–perceptual means of constructing a safe place. In general, art therapy can provide an avenue for helping individuals understand their thoughts and feelings, serve as a medium to express oneself, and explore the concept of psychological safety (e.g., Nielsen et al., 2019). Referring to PTSD treatment, art therapy has also been used for the purpose of consolidating traumatic memories, creating a coherent trauma narrative, and allowing for nonverbal expression of traumatic experiences (e.g., Wertheim-Cahen, 1991; Smeijsters, 2008; Gantt and Tinnin, 2009; Van Lith, 2016). Art therapy may also be useful for the direct management of distress and physical symptoms resulting from traumatic events (e.g., Ballou, 1995; Cohen et al., 1995; Morgan and White, 2003; Rankin and Taucher, 2003; Lyshak-Stelzer et al., 2007). For example, Hass-Cohen et al. (2018) demonstrated among a group of trauma-exposed participants that drawing pictures of the self, the problem, and coping resources significantly reduced the rating of the effect of the traumatic event, negative affect, and increased endorsement of resiliency resources, whereas Henderson et al. (2007) showed that three 20-min mandala drawing sessions were associated with significantly reduced PTSD symptoms compared to a control condition. Further, among persons with combat-associated PTSD, eight sessions of art therapy combined with cognitive processing therapy (CPT) resulted in significantly reduced PTSD symptoms compared to an equal number of sessions of CPT when combined with supportive psychotherapy (Decker et al., 2018). Indeed the use of art therapy for the treatment of mental health conditions represents a field of growing interest (e.g., Van Lith, 2016; Haeyen et al., 2018), and previous literature has examined the use of art therapy for treating a range of trauma-related psychological disorders including depression, bipolar disorder, schizophrenia, and PTSD (e.g., Lyshak-Stelzer et al., 2007; Crawford et al., 2012; Van Lith, 2015).

However, use of art therapy exercises as implemented in the aforementioned clinical literature has used traditional art materials such as paint and pencil and paper, and to our knowledge, the ability of VR technology to provide an immersive modality for artistic expression within the context of psychotherapy for PTSD has not yet been studied. The present study therefore sought to be the first to compare affective responses associated with (1) simply imagining a safe space without the aid of a computer (IMG format), (2) drawing a 2-D safe space using a laptop and the standard Microsoft Paint application (2-D format), and (3) creating a three-dimensional safe space using the VR application Google Tilt Brush (VR format).

### Study 1 Methods

#### Participants

The sample comprised 36 (29 females) university students. About three of every four (*n* = 27, 75%) of the participants reported that they had experienced at least one type of ACE (i.e., before the age of 18 years) (mean = 2.50, SD = 2.26, min. = 0, max. = 7); the same number (*n* = 27, 75%) had experienced at least one type of traumatic life event as an adult as measured by the LEC-5 (mean = 2.06, SD = 2.07, min. = 0, max. = 8), and all participants had experienced at least one type of stressful life event in the past year as measured by the LES (*M* = 11.22, SD = 5.18, min. = 4, max. = 21). Twelve participants (33%) scored above the recommended cutoff of 33 for probable *Diagnostic and Statistical Manual of Mental Disorders, Fifth Edition* (*DSM-5*) PTSD diagnosis on the PCL-5 (*n* = 12), and the overall sample PCL-5 descriptive statistics was as follows: mean = 24.36, SD = 15.02. Among the 12 participants who met probable *DSM-5* PTSD diagnosis on the PCL-5, four (33%) also met probable diagnosis of the dissociative subtype of PTSD (D-PTSD) as measured by a score of at least three on at least one of the two TRASC items described by Frewen et al. (2015). Multivariate effects comparing responses to the baseline questionnaires between groups were nonsignificant in all cases, implying that the groups can be assumed to be equivalent at baseline irrespective of the ORDER in which they were randomized to complete the exercises in.

#### Task Formats and Instructions

The VR software application Google Tilt Brush was utilized in order to administer the VR format of the exercise^3^. In this application, while wearing the HMD, users utilize the VR hand controllers to draw whatever they wish, making use of various options for types of paint brushes, color, stimuli, backgrounds, and so on. First, participants were given a brief tutorial describing how to use the application. Following completion of the tutorial, instructions for the exercise given to participants were as follows: “Please use the application to create a safe space. This safe space can include, but is not limited to, the creation of a scene, item, design, or any visual representation that makes you feel safe.” By comparison, for the 2-D computer format of the exercise, the standard Paint application in Windows 10 was used^4^. Participants were again provided a brief tutorial on the use of the application, and participants were given the same instructions. Finally, for the noncomputerized format of the exercise, participants were instructed as follows: “Please use your imagination to think of, and imagine yourself in, a safe space. This safe space can include, but is not limited to, imagining a scene, item, design, or any visual representation that makes you feel safe.” Participants took as much time as they wished with each exercise, indicating when they were done verbally, usually within about 10 min. During debriefing following each exercise, participants described the scenes they imagined or constructed and what they experienced during each format of the task.

### Study 1 Results and Discussion

**FIGURE 2:**
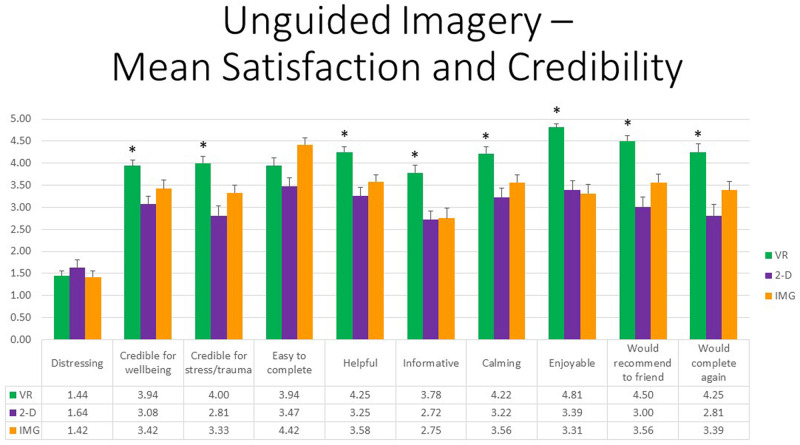
Bars illustrate the mean ratings obtained on a 0- to 5-point scale for the VR (green), 2-D (purple), and IMG (orange) formats of the task, also written in the embedded table below. Error bars illustrate the standard error of measurement. Asterisks denote the 8 of 10 ratings in which the mean rating for the VR condition significantly exceeded the mean rating for both of the 2-D and IMG conditions. Any other statistically significant pairwise comparisons are described in the text. IMG, imagery; 2-D, two-dimensional; VR, virtual reality.

Referring to response to the 10 items measured by the satisfaction and credibility questionnaire, at the multivariate level, first, referring to within-group effects, the main effect of TYPE was highly statistically significant, *F*(20,14) = 6.666, *p* < 0.001, η^2^ = 0.905, whereas the effect of ORDER or the interaction between ORDER and TYPE was not. At the univariate level, the within-group main effect of TYPE was statistically significant for all satisfaction and credibility ratings except in the case of “distressing” (*p* = 0.410). All follow-up paired *t* tests indicated that participants were more satisfied in response to the VR exercise in comparison to the 2-D computer exercise except in the case of “easy to complete,” for which the difference fell short of statistical significance (*p* = 0.054). Further, all follow-up paired *t* tests indicated that participants were more satisfied in response to the VR exercise in comparison with the mental imagery exercise except in a single case whereby participants rated mental imagery to be more “easy to complete” than the VR exercise (*p* = 0.039). Note that obtained effect sizes for these paired comparisons involving the VR format were large in most instances (i.e., *d′ >* 0.80); see Supplementary Material for full tabled effect sizes for all pairwise comparisons. Finally, mental imagery was regarded as more “credible as an intervention for mental health problems associated with stressful/traumatic life events” and was also considered more “easy to complete” in comparison to the 2-D computer exercise. Moreover, participants reported that they would more highly “recommend this exercise to a friend” and would more likely “complete this exercise again” in reference to the mental imagery exercise when compared to the 2-D computer exercise. Results are shown in Figure 2. We therefore conclude that the safe place exercise produced the greatest perceived satisfaction and credibility as an intervention for trauma- and stressor-related disorders and mental health and well-being more generally when completed within the immersive format of VR as compared to standard laptop screen (2-D) format or using visual imagery alone, whereas visual imagery was also preferred over the 2-D format in certain respects.

**FIGURE 3:**
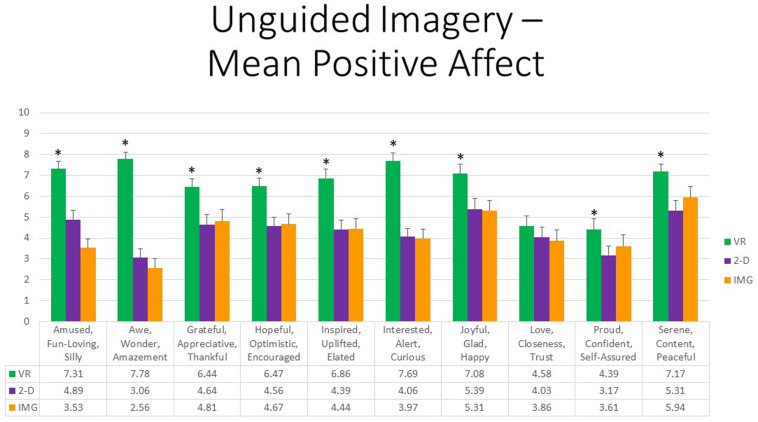
Bars illustrate the mean ratings obtained on a 0- to 10-point scale for the VR (green), 2-D (purple), and IMG (orange) formats of the task, also written in the embedded table below. Error bars illustrate the standard error of measurement. Asterisks denote the 9 of 10 ratings in which the mean rating for the VR condition significantly exceeded the mean rating for both of the 2-D and IMG conditions. Any other statistically significant pairwise comparisons are described in the text. IMG, imagery; 2-D, two-dimensional; VR, virtual reality.

Referring to 10 positive affective states measured by the mDES-PA items, at the multivariate level, the main effect of exercise FORMAT was also highly statistically significant, *F*(20,14) = 14.28, *p* < 0.001, η^2^ = 0.95, whereas the effects of ORDER and the interaction between ORDER and FORMAT again were not. At the univariate level, the within-group main effect of FORMAT was statistically significant for all positive emotions except in the case of “love, closeness, trust” (*p* = 0.252). All follow-up paired *t* tests indicated that participants experienced greater positive emotions in response to the VR exercise in comparison to both the 2-D computer exercise and noncomputer exercise, whereas the latter two conditions differed only in response to a single rating, “amused, fun-loving, and silly,” whereby the 2-D computerized task received the higher rating; results are depicted in Figure 3. Further, obtained effect sizes for these paired comparisons involving the VR format were also typically large (i.e., *d′ >* 0.80); see Supplementary Material. We therefore conclude that the safe place exercise produced the greatest positive affect when completed within the immersive format of VR as compared to constructing the safe place using a standard laptop screen or using visual imagery alone.

**FIGURE 4:**
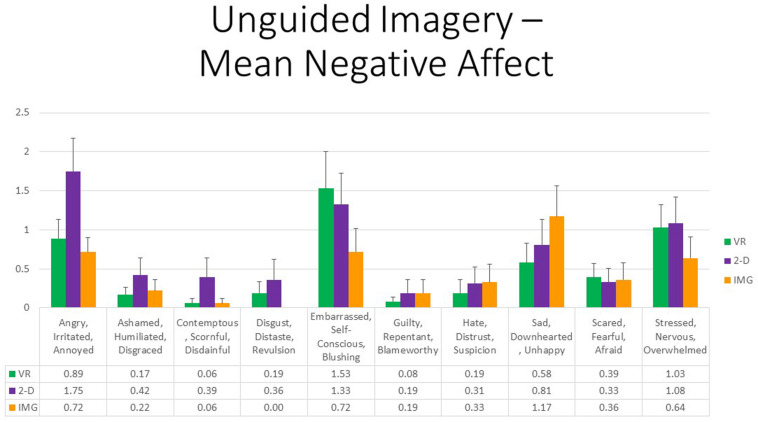
Bars illustrate the mean ratings obtained on a 0- to 10-point scale for the VR (green), 2-D (purple), and IMG (orange) formats of the task, also written in the embedded table below. Error bars illustrate the standard error of measurement. In the absence of a significant ANOVA, no pairwise comparisons were conducted but rather all are assumed to be nonsignificant. See text for further description. IMG, imagery; 2-D, two-dimensional; VR, virtual reality.

Finally, referring to 10 negative affective states measured by the mDES-NA survey, at the multivariate level, no between- or within-group main effects or interactions were statistically significant; therefore, no follow-up univariate tests were conducted. To afford comparability with the findings reported for positive affect, Figure 4 also displays results for all self-reported negative emotions. However, whereas the vertical axis referring to positive affect in Figure 3 runs between 0 and 10, the same axis in Figure 4 referring to negative affect is fourfold smaller. As a result, we conclude that negative affect of any kind was reported only very rarely or at a very low intensity in response to any format of the exercise. Some participants did report a mild sense of feeling “embarrassed, self-conscious, blushing” while completing the exercises on computer, primarily relating to their believing they were of low artistic skill when completing the exercise, and their ongoing work was viewable to the experimenter; this variably occurred in both the VR and 2-D conditions.

**FIGURE 5:**
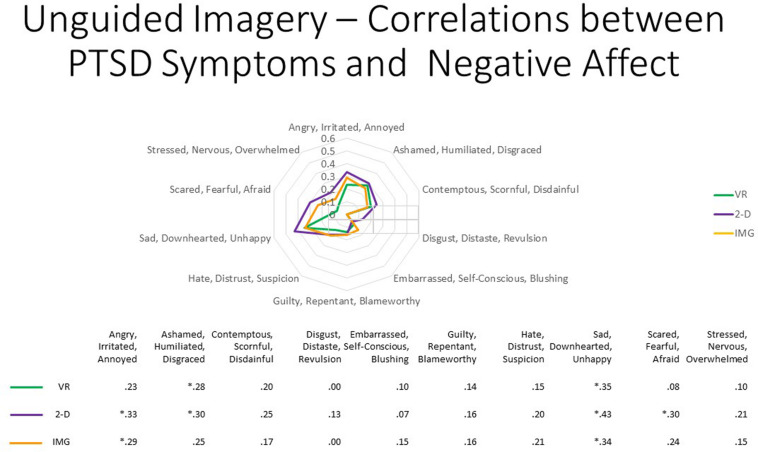
The radar figure further depicts the pattern of the correlations noted in the table below between PTSD symptoms (measured by the PCL-5), on the one hand, and the various negative affect ratings (measured by the mDES), on the other. Asterisks denote the 8 of 30 ratings in which the correlation is statistically significant at *p* < 0.05 (1-tailed). See text for further description. IMG, imagery; 2-D, two-dimensional; VR, virtual reality.

**FIGURE 6:**
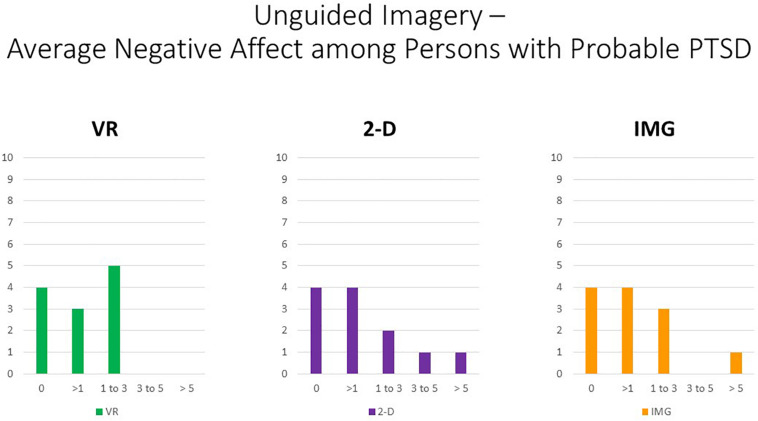
Bars illustrate the number of participants in the sample with probable PTSD (i.e., the subsample scoring above 33 on the PCL-5) whose average score across the 10 negative affect ratings on the MDES exceeded the values indicated on the *x* axis. It can be seen that very few scored above even a relatively low value of 3 on the 0- to 10-point scale for any of the task formats: VR (green), 2-D (purple), and IMG (orange). See text for further description. IMG, imagery; 2-D, two-dimensional; VR, virtual reality.

Nevertheless, persons with higher PTSD symptoms reported greater negative affective states in response to the tasks, especially with regard to feeling “angry, irritated, annoyed,” “ashamed, humiliated, disgraced,” and “sad, downhearted, unhappy,” responses that were relatively consistent across task formats and averaged around a correlation of *r* ≈ 0.20 (Figure 5). We therefore conclude that participants with PTSD are likely at increased risk of responding with increased negative affect to the task of unguided “safe place” imagery regardless of the format in which it is administered. Nevertheless, supplementary analyses found that, even among the 12 participants who scored above the recommended cutoff of 33 for probable *DSM-5* PTSD diagnosis on the PCL-5, the majority had an average mDES-NA score of less than 1 on the 0- to 10-point scale across the 10 emotional states surveyed, and only a single person had an average score of 5 or more on the 0- to 10-point scale for any format (Figure 6). This emphasizes the rarity of clinically significant negative affective responses observed among any participant in the present study, including even among those who reported higher PTSD symptoms.

Regarding the thematic analysis of open-ended comments, seven themes were observed by at least four (i.e., >10% of) participants, which were, in decreasing order of observed frequency: (1) positive affect (*n* = 14), (2) calming (*n* = 14), (3) fun–interesting (*n* = 11), (4) safe–secure (*n* = 7), (5) vividness–realism (n = 6), (6) presence (*n* = 6), and (7) positive memories (*n* = 5). However, the first three themes were entirely redundant with the survey items of the mDES-PA, and so these will not be discussed further. In comparison, referring to the “safe–secure” theme, participants commented on the sense of feeling safety and security while completing the VR task, for example, stating that: “I really like bright things so the stars and sun make me feel safe. I used warm colors, which also makes me feel safe”; and “I drew some objects that are important to me that feel safe: a mug, a wine glass, I drew two lamps…”; and “I made a room, and usually I’m with my boyfriend, and he makes me feel really comfortable, and that’s where I feel safe.” Referring to the “vividness–realism” theme, participants commented on the vivid, realistic nature of the VR task, often associated with the immersive, interactive nature of the task, for example, stating that: “It felt more realistic… It felt more real”; and “It was immersive”; and “It almost felt real with all of the features.” Referring to the “presence” theme, participants commented on the sense in which the VR experience created an immersive, egocentric frame of reference, for example, “I was imagining myself in the room”; and “I feel like I’m actually in the space”; and “As I was making it and as it became more complete, I actually felt like I was in a room.” Finally, referring to the “positive memories” theme, participants commented that the VR task invoked positive memories, for example, “The drawing is supposed to mimic this carpet that is in my grandparent’s house… A lot of my fond childhood memories happened there”; and “I created a beach… while I was doing it, I started to have good memories of travelling with my family years ago”; and “I drew my friend and I at a museum… I haven’t seen the friend in a while, and it reminded me of her, so that was really nice. It made me miss her.”

The mean number of themes identified in participants’ comments was 2.31 (SD = 1.26). Two participants’ comments were coded to exhibit five or more themes, thus being approximately two standard deviations above the mean. These participants’ comments are noted in full in order to share some of the qualitatively richer phenomenological descriptions received from participants regarding the ways in which they responded to this VR unguided imagery task. Specifically, the first participant, whose response was coded to have six themes, commented:

“I used the brushes and colors to create patterns I think are interesting and triggers my creativity. It’s not a “place” but a scene I feel comfort in and can explore myself. It’s almost like a different dimension. I created a tunnel that almost teleports you to another place. There is a sense of mystery and sense of unknown. There is a sun that symbolizes happiness and warmth. There are stars and bubbles that remind me of my childhood.”

Further, the second participant, whose response was coded to have ‘five themes, commented:

“That was, in one word, awesome. I loved the freedom of space around me, because it gave me more to work with than a 2-D space, I felt more free, it’s shocking to see how you can change the environment. The amount of tools available allowed me to explore and that was fun and freeing. I think it made me feel better than the first activity because it made me more excited to draw myself into the safe place and felt more at ease.”

## Study 2: Guided Autobiographical Recall

Research shows that narrative therapy can also produce positive clinical outcomes, for example, reducing symptoms of depression compared to wait list over long-term follow-up (e.g., Lopes et al., 2014a, b) and reducing PTSD among adult and child refugees, asylum seekers, survivors of mass violence and torture, and earthquake survivors (e.g., Robjant and Fazel, 2010; McPherson, 2012; Zang et al., 2013). The conceptual framework underlying narrative therapy (White and Epston, 1989) and narrative exposure therapy (Neuner et al., 2002) is to view the collection of life events that a person has experienced as a library of personal stories and to recognize that a certain number of these stories may be significant determinants of identity, commonly known in the PTSD literature as “self-defining memories” (e.g., Conway and Pleydell-Pearce, 2000; Sutherland and Bryant, 2005; Singer et al., 2013). Through imagination and episodic recall, however, narrative therapy involves “revisiting” such memories in order to reflect on the meanings people ascribed to their life experiences and to potentially make new meanings where appropriate as a psychotherapeutic means. Within narrative therapy, doing so, especially when highly negative appraisals and attributions can be “rewritten” more adaptively, is considered a practice of “reauthoring identity” that can bring about therapeutic change.

Further, narrative exposure therapy, as a particularly structured delivery of narrative therapy, includes creation of a narrative of all of the client’s stressful life events in chronological order, modifying and elaborating the list until the traumatic events they have experienced are embedded within the context of an autobiography (Neuner et al., 2002). This approach therefore emphasizes the relevance of assessing trauma history from a developmental life course perspective inclusive of events from childhood and adulthood. Moreover, this otherwise private autobiography may further stand as a form of public testimony to traumas that occurred on a population scale, for example, among traumatized refugees from low-income and war-torn countries and other victims of severe human rights violations (Neuner et al., 2002).

Although usually implemented as imaginative or talk therapy, narrative exposure therapy can also be administered in other formats, for example, as writing therapy, and there is accumulating evidence for its efficacy and acceptability as a treatment delivered in various formats (e.g., van Emmerik et al., 2013). However, to our knowledge, no prior studies have investigated affective responses to immersive, visual processing of autobiographical stimuli in persons with PTSD, for example, as perceived by viewing 360° (spherical) photographs of autobiographical stimuli through an HMD. With this in mind, we conceived that a safe “revisiting” of important location-based landmarks relevant to personal history and identity (e.g., childhood and adult home, school) could occasion meaningful episodic recall, self-reflection, and associated affective responses as a brief structured psychological intervention consistent with the narrative therapy approach. In Study 2, we therefore compared affective responses to traditional uncued recall and visualization of a person’s childhood and adult home and school (IMG format) to visually cued recall of the same environments when administered in 2-D versus VR formats.

### Study 2 Methods

#### Participants

The sample comprised 48 (28 females) undergraduate university students. Half of the participants (*n* = 24, 50%) reported that they had experienced at least one type of ACE (i.e., before the age of 18 years) (mean = 1.56, SD = 1.99, min. = 0, max. = 7); approximately two in every three participants (n = 33, 69%) had experienced at least one type of traumatic life event as an adult as measured by the LEC-5 (mean = 1.90, SD = 1.90, min. = 0, max. = 7), and all participants had experienced at least one stressful life event in the past year as measured by the LES (mean = 12.23, SD = 6.11, min. = 1, max. = 30). One in every four participants (25%) scored above the recommended cutoff of 33 for probable *DSM-5* PTSD diagnosis on the PCL-5 (*n* = 12), and the overall sample PCL-5 descriptive statistics was as follows: mean = 22.50, SD = 15.13. Among the 12 participants who met probable *DSM-5* PTSD diagnosis on the PCL-5, four (33%) also met probable diagnosis of D-PTSD. Multivariate effects comparing responses to the baseline questionnaires between groups were nonsignificant but trending (*p* = 0.062), causing a hazard that the groups might not to be equivalent at baseline despite randomization. Follow-up univariate analyses suggested that, unfortunately, despite randomization to groups, those who completed the VR intervention first reported overall greater PTSD symptoms, dissociative experiences (TRASC), anxiety, and depression symptoms at baseline (*p* < 0.05).

#### Task Formats and Instructions

The VR software application Google Earth VR was utilized in order to administer the VR FORMAT of the exercise^5^, whereas the 2-D computer FORMAT of the exercise used the more familiar standard (non-VR) desktop version of the same software^6^. All task formats were completed in seated position in a standard office roller-swivel chair. In the VR application, while wearing the HMD, users utilize the VR hand controllers to navigate a computer graphical rendering of the earth’s surface, with the “street view” functionality further providing the viewing of 360° spherical photographs of certain locations typically taken from a roadside position and ordered by the global positioning system (GPS). Participants were asked to wear the HMD and were introduced to the application through an approximately 5-min tutorial on how to use the controllers provided by the VR Google Earth application itself, including how to search for locations, how to orient themselves in street view, and how to maneuver through the locations. The tutorial also served as a way to orient and habituate the participants to the experience of VR. Similar instructions were provided with regard to use of the non-VR version of the Google Earth application, although many participants were already familiar with the use of this software.

Besides the instructions given, in randomized order, participants were asked to locate their childhood and adult homes and places of schooling in the various computerized formats using either the search functionality by address if known or by navigating by visual memory if not known, or simply recalled how each place looks and imagined “seeing” themselves in each place in their mind’s eye in the noncomputerized IMG format of the task. In the computerized formats, the locations were explored in street view if available until the participant was satisfied, including having the opportunity to navigate within nearby GPS coordinates within the VR format using the “blink teleport” functionality within the VR version of the application. Experientially this was akin to “walking” up and down various streets using the hand controllers, while behaviorally this involved looking around the virtual environment using head movements and swiveling in their roller chairs. Once participants were satisfied with each experience, the researcher transcribed participants’ responses to the open-ended questions and rating surveys that were administered. Although responses were collected separately to the childhood and adult locations, results are presented as averaged across these responses to adhere to the research design used in Study 1.

### Study 2 Results and Discussion

**FIGURE 7:**
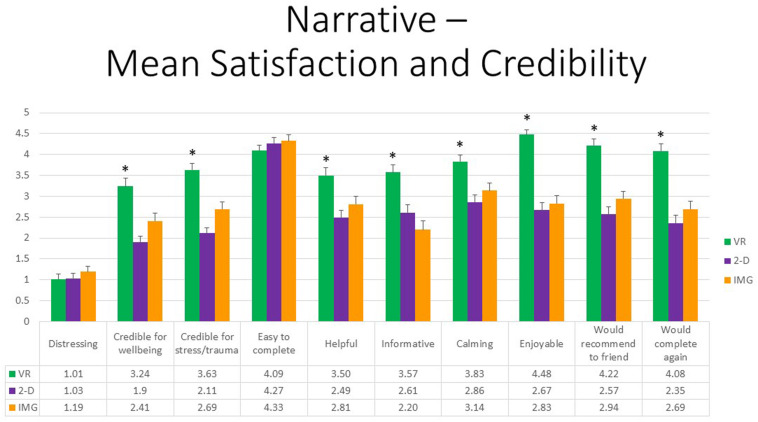
Bars illustrate the mean ratings obtained on a 0- to 5-point scale for the VR (green), 2-D (purple), and IMG (orange) formats of the task, also written in the embedded table below. Error bars illustrate the standard error of measurement. Asterisks denote the 8 of 10 ratings in which the mean rating for the VR condition significantly exceeded the mean rating for both of the 2-D and IMG conditions. Any other statistically significant pairwise comparisons are described in the text. IMG, imagery; 2-D, two-dimensional; VR, virtual reality.

First, referring to the 10 items assessed by the satisfaction and credibility questionnaire, at the multivariate level, referring to within-group effects, the main effect of FORMAT was again highly statistically significant, *F*(20,27) = 12.44, *p* < 0.001, η^2^ = 0.90, whereas the effect of ORDER was not. However, it should also be noted that the interaction between ORDER and FORMAT trended toward significance, *F*(20,27) = 1.88, *p* = 0.06, η^2^ = 0.58. At the univariate level, the within-group main effect of FORMAT was statistically significant for all satisfaction and credibility ratings except in the case of “distressing” (*p* = 0.17) and “easy to complete” (*p* = 22). Concerning follow-up paired *t* tests of the main effect, participants were more satisfied in response to the VR exercise in comparison to both the 2-D computer and noncomputer exercise for all ratings. Moreover, obtained effect sizes for these paired comparisons involving the VR format were large in most instances (i.e., *d′ >* 0.80); see Supplementary Material for listing of effect sizes for all pairwise comparisons. By comparison, the noncomputer format of the exercise was regarded as a more “credible way to improve self-regulation and enhance well-being” (*p* = 0.02) and as more “credible as an intervention for mental health problems” (*p* < 0.01) when compared with the 2-D computer format, whereas the reverse was true regarding the rating of “informative” (*p* = 0.03); several other ratings evidenced trend-level significance (*p* < 0.10). Results are shown in Figure 7. By contrast, no univariate effects were statistically significant for the interaction between FORMAT and ORDER, and so no follow-up paired *t* tests were conducted. We therefore conclude that the narrative exercise produced the greatest perceived satisfaction and credibility ratings as an intervention when completed within the immersive format of VR as compared to standard laptop screen format or using imagery and recall alone.

**FIGURE 8:**
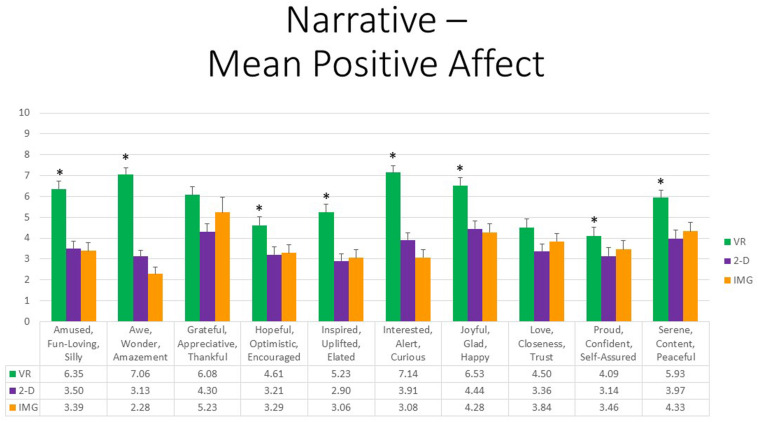
Bars illustrate the mean ratings obtained on a 0- to 10-point scale for the VR (green), 2-D (purple), and IMG (orange) formats of the task, also written in the embedded table below. Error bars illustrate the standard error of measurement. Asterisks denote the 8 of 10 ratings in which the mean rating for the VR condition significantly exceeded the mean rating for both of the 2-D and IMG conditions. Any other statistically significant pairwise comparisons are described in the text. IMG, imagery; 2-D, two-dimensional; VR, virtual reality.

Referring to the 10 positive affective states measured by the mDES-PA survey, at the multivariate level, the main effect of exercise FORMAT was also highly statistically significant, *F*(20,27) = 7.00, *p* < 0.001, η^2^ = 0.84, whereas the effects of ORDER and the interaction between ORDER and FORMAT were not. At the univariate level, the within-group main effect of FORMAT was statistically significant for all positive emotions without exception. All follow-up paired *t* tests further indicated that participants experienced greater positive emotion in response to the VR exercise in comparison to the 2-D computer exercise. Additionally, follow-up paired *t* tests indicated that participants experienced greater positive emotion in response to the VR exercise in comparison to the noncomputer exercise with the exception of “grateful, appreciative, thankful” (*p* = 0.15) and “love, closeness, trust” (*p* = 0.06). Note further that obtained effect sizes for these paired comparisons involving the VR format were also large in the majority of instances (i.e., *d*’ *>* 0.80); see Supplementary Material. Finally, the 2-D computer exercise was associated with more “awe, wonder, amazement” when compared with the noncomputer IMG format of this exercise (*p* = 0.01), whereas all other comparisons were nonsignificant. Results are depicted in Figure 8. We therefore conclude that again the narrative exercise produced the greatest positive affect when completed within the immersive format of VR as compared to completing the same exercise using a standard laptop screen or using visual imagery and episodic recall alone.

**FIGURE 9:**
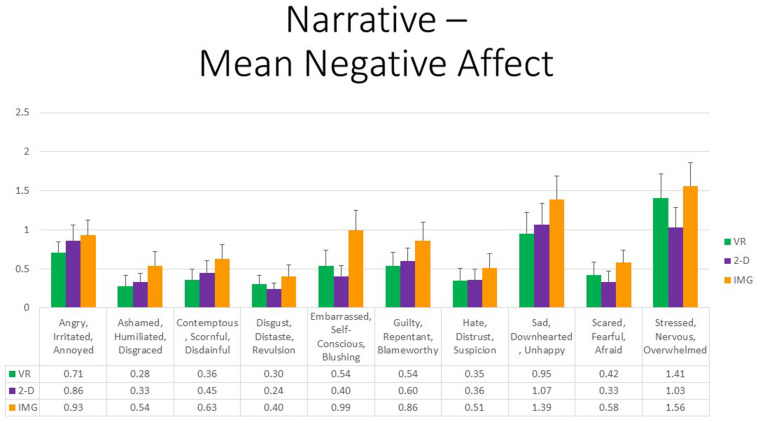
Bars illustrate the mean ratings obtained on a 0- to 10-point scale for the VR (green), 2-D (purple), and IMG (orange) formats of the task, also written in the embedded table below. Error bars illustrate the standard error of measurement. In the absence of a significant ANOVA, no pairwise comparisons were conducted, but rather all are assumed to be nonsignificant. See text for further description. IMG, imagery; 2-D, two-dimensional; VR, virtual reality.

Finally, referring to the 10 negative affective states measured by the mDES-NA survey, at the multivariate level, no between- or within-group main effects were statistically significant. Further, although the multivariate interaction between FORMAT and ORDER trended toward significance, *F* (20,27) = 1.98, *p* = 0.05, η^2^ = 0.59, follow-up univariate tests were nonsignificant in all cases. To afford comparability with the findings reported for positive affect, Figure 9 also displays results for self-reported negative affects. Note, however, again the difference in scaling, suggesting that negative emotional responses to the narrative exercise in any format were rare or of minimal intensity.

**FIGURE 10:**
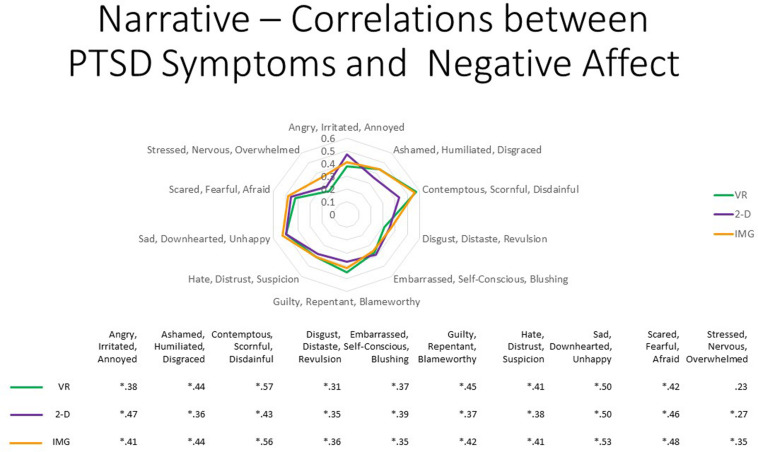
The radar figure further depicts the pattern of the correlations noted in the table below between PTSD symptoms (measured by the PCL-5), on the one hand, and the various negative affect ratings (measured by the mDES), on the other. Asterisks denote the 29 of 30 ratings in which the correlation is statistically significant at *p* < 0.05 (1-tailed). See text for further description. IMG, imagery; 2-D, two-dimensional; VR, virtual reality.

**FIGURE 11:**
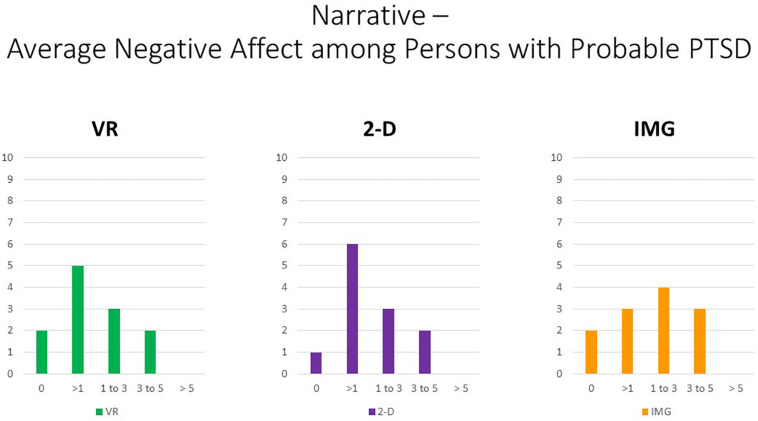
Bars illustrate the number of participants in the sample with probable PTSD (i.e., the subsample scoring above 33 on the PCL-5) whose average score across the 10 negative affect ratings on the MDES exceeded the values indicated on the *x* axis. It can be seen that very few scored above even a relatively low value of 3 on the 0- to 10-point scale for any of the task formats: VR (green), 2-D (purple), and IMG (orange). See text for further description. IMG, imagery; 2-D, two-dimensional; VR, virtual reality.

That observation notwithstanding, concerning participant symptoms of PTSD, Pearson correlations again suggested that persons with higher PTSD symptoms reported greater negative affective states in response to the tasks for all negative emotions measured, responses that were highly consistent and did not differ appreciably across task formats (Figure 10, where the effect sizes average approximately *r* ≈ 0.35). We therefore must again conclude that participants with probable PTSD are likely at increased risk of responding with increased negative affect to the narrative task irrespective of task format. Even still, supplementary analyses found that, even among the 12 participants who scored above the recommended cutoff of 33 for probable *DSM-5* PTSD diagnosis on the PCL-5, the majority again had an average mDES-NA score of less than one on the 0- to 10-point scale across the 10 emotional states surveyed, and only a single person had an average score of 5 or more on the 0- to 10-point scale for any format (Figure 11).

Regarding the thematic analysis of open-ended comments, in considering that participants responded to the task as a response to both their childhood and adulthood locations, we found that seven themes were observed at least 10 times (i.e., >10% of participant ratings), namely, (1) fun–interesting (*n* = 55), (2) memories of unstated valence (*n* = 33), (3) positive affect (*n* = 25), (4) vividness–realism (*n* = 20), (5) positive memories (*n* = 19), (6) nostalgia (*n* = 16), and (7) negative affect (*n* = 11). Again, themes 1, 3, and 7 were wholly redundant with response to the mDES survey and so will not be discussed further. Referring to the “memories of unstated valence” theme, participants described the task as invoking episodic recall, but whether the memories were positive or negative could not be determined with certainty, in most cases seeming either neutral or only mildly positive. For example, participants described: “I saw the field where I used to play football and thought about all the times I walked to and from school in the evenings”; and “Campus—remembering my first day on campus, figuring out how to get around. Home—I thought about walking my dog—I walk him around there every day” and “I remembered specific events like moments of my life when I was there.” Regarding the previously described “vividness–realism” theme, examples to the narrative exercise included “It was weird how realistic it is… because when I think about it [referring to the IMG task] I know it’s supposed to be there but I can’t see—but the VR shows details and brings back more memories”; and “More immersed, can move around and interact—kind of explore areas where you had experiences as a child”; and “It was also immersive. I saw more than in the 2-D condition. It made me more in touch with those spaces. It felt more real.” Regarding the previously described “Positive Memories” theme, examples in response to the narrative exercise included “I guess because I was thinking about my childhood I remembered all the fun we had at school, with neighbors, with friends. Happy, missing it a little. Being thankful that I had a really good childhood there”; and “It was a little emotional to see where I remember being young, naive, innocent; its heartwarming. When I saw the school, I was actually laughing inside because I thought about funny situations that have happened to me”; and “I really enjoyed it. I felt warm, and it brings back happy memories.” Finally, as a closely related theme, coded instances of the “Nostalgia” theme included “Saw my car which brought back memories of family and the longing”; and “Really interesting and nostalgic, and it brought back old memories”; and “When I saw my home it was nostalgic—I still live there sometimes. I saw my old car and some old features—saw my grandma there—reminded me of growing up.”

The mean number of themes identified in participants’ comments in response to the task when referring to adult locations was 2.30 (SD = 1.44), whereas for the child locations, this was 3.24 (SD = 1.77). Three participants’ comments were coded to exhibit seven or more themes, thus being approximately two standard deviations above either mean, indicative of some of the richer phenomenological descriptions we received. Specifically, one participant, whose response was coded to have seven themes, commented:

“Did a lot of talking while experiencing. The feeling is fleeting, but the memory or the emotion behind it was more intense. Remembered more specific things. My parents divorced right before we moved out of the house—so the house has more memories—nostalgic and happy—warm fuzzy feelings. The yellow warm color of the house might have induced warm feelings. There were bad memories with the divorce, but I didn’t think of that as much. Compared to 2-D it felt warmer. Thought more about the divorce in imagination. 2-D was just like a picture but in VR it felt more personal. School—specific details and memories, would talk about life. The proportions of things were clearer and you realize that when you’re a kid everything looks big. It’s a sunny setting—I thought about the peaceful summer days. For the most part, a happy childhood.”

Another participant, whose comments were coded to exhibit eight themes, reported:

“I’ve never done VR so that was something new. Much clearer experience than looking at a screen. It’s like everything is right there, but everything is static. I was reminded of some things that didn’t come to mind when doing 2-D. Like when I biked it was scary because Toronto drivers are reckless. So having to be really careful. This was all on the road, and I was right there on that bike lane. Specific scenarios of drivers doing some really shady things and realizing I could have died right there. Overall seeing the architecture and everything was really spot on. Mostly thought about how dangerous it is biking in Toronto. Frustration because of those experiences, sometimes anger, when people break road rules. You quickly get over it, though. In general—definitely closer to what life was like over there. Some sense of appreciation—I was there. It’s a great school; campus is great. Appreciation for not just education but the community. Grateful. Sense of belonging with complete strangers. Thought about convocation—that was a great experience. More bittersweet. Sometimes we’re too critical about ourselves, but at the same time I made it through, so there is that sense of happiness. You think of your friends who helped you out. You’re all there.”

Finally, the third participant, whose response was coded to have 10 themes, commented:

“Such an incredible feeling to be honest. The premise of what you guys are doing is really good. I study a lot about experience—there is a lot of intrinsic value—higher sense of self and worth. Music helps too, a really positive feeling. Like being really excited for the day and studying and getting an education. Home—spent a lot of time outside. Especially with birds. Loved studying things and remembering who I am. School—people telling you to do things, boring and isolated, brought back the stress that my mom put on me and teachers telling me how to be. The field—I also felt the way I was at home, discovering things, having friends, doing my own thing. School was more negative but still positive, and home was more supportive.”

## Study 3: Guided “Wilderness” Imagery

Coupled to standard individual and group-based psychotherapy, “wilderness therapy,” otherwise known as “adventure therapy,” involves the organized engagement in outdoor environmental experiences or “adventures” partly in order to provoke positive emotions and encourage social and family engagement and effective problem solving (e.g., Russell and Phillips-Miller, 2002). Wilderness therapy is often conducted in residential settings with adolescents including those who have recently experienced traumatic stressors (e.g., Bettmann et al., 2011), and there is an increasing emphasis on delivering interventions within the framework of trauma-informed care (Norton et al., 2019). The wilderness-based activities available to participants variably include hiking, canoeing or kayaking, cross-country skiing, snow-shoeing, rock-climbing, and related activities (Tucker and Norton, 2013). Recent meta-analyses show that wilderness therapy has proven efficacy for improving psychosocial outcomes on a range of measures (Bettmann et al., 2016; Gillis et al., 2016). For example, trauma-exposed youth and their families who participated in a wilderness therapy experience as an adjunct to standard trauma-focused treatment reported greater reduction in depression, anxiety, anger, and PTSD symptoms and improved family function when compared with youth and families who received only the standard trauma-focused care (i.e., treatment as usual, excluding the outdoor environmental experiences). Together with the common factors that are intrinsic to wilderness therapy attributable to its inclusion of psychoeducation and individual and group psychotherapy (e.g., Russell et al., 2017), it is thought that positive psychosocial outcomes brought about by wilderness therapy will be at least partially mediated by the unique immersive, naturalistic outdoor experiences that are provided. For example, experiences conducted in outdoor environments such as forests and mountainous areas can provoke highly positive emotions such as the appreciation of beauty, wonder, and awe (e.g., Anderson et al., 2018).

Unfortunately, however, access to therapeutic experiences in wilderness settings will be limited to most urban persons, and given that wilderness therapy is a highly integrative intervention that comprised many elements, it is relatively difficult to study. By contrast, guided (script-driven) imagery of the experience of naturalistic environments is comparably straightforward to administer and could be a basis for bringing about positive emotions as well. Indeed Holmes et al. (2016) review findings that depression is associated with impoverished positive imagery, providing a rationale for practice of positive imagery as an intervention for depression. However, Holmes et al. also discuss that depression may result in the impoverishment of positive imagery vividness, potentially limiting the impact of mental imagery-based interventions alone.

As such, exploration of VR used to provide a vivid medium for perceptual immersion into realistic, naturalistic environments as a means of inducing positive emotions is necessary. Although we assume that the most effective therapeutic means for inducing positive affect and the awe-inducing appreciation of nature will be through *in vivo* experience of real-world natural environments, the present research evaluated whether positive emotions can also be engendered by immersing oneself in realistic computer graphical renderings of naturalistic environments in VR as compared with viewing the same environments in standard flat-screen (2-D) computer format and imagining the same kinds of scenes using mental imagery alone (IMG format).

### Participants

The Study 3 sample comprised 48 (28 females) undergraduate university students. Approximately two of every five participants (*n* = 21, 44%) reported that they had experienced at least one type of ACE (i.e., before the age of 18 years) (mean = 1.08, SD = 1.62, min. = 0, max. = 7); approximately two of every three participants (*n* = 31, 65%) had experienced at least one type of traumatic life event as an adult as measured by the LEC-5 (mean = 1.04, SD = 1.03, min. = 0, max. = 4), and all participants had experienced at least one type of stressful life event in the past year as measured by the LES (mean = 8.75, SD = 4.14, min. = 1, max. = 19). Approximately one in every four participants (27%) scored above the recommended cutoff of 33 for probable *DSM-5* PTSD diagnosis on the PCL-5 (*n* = 13), and the overall sample PCL-5 descriptive statistics were: mean = 24.36, SD = 15.02. Among the 13 participants who met probable *DSM-5* PTSD diagnosis on the PCL-5, two (15%) also met probable diagnosis of D-PTSD as measured by a score of at least three on at least one of the two TRASC items described by Frewen et al. (2015). Multivariate effects comparing responses to the baseline questionnaires between groups were nonsignificant in all cases, implying that the groups can be assumed to be equivalent at baseline irrespective of the ORDER in which they were randomized to complete the exercises in.

### Task Formats and Instructions

The VR software application “*NatureTreks*,” was utilized in order to administer the VR FORMAT of the exercise^7^. In this application, while wearing the HMD, users utilize the VR hand controllers to navigate seasonal scenes in naturalistic environments, as well as to modify the environment in various ways (e.g., planting trees or flowers, causing it to rain or snow, changing the degree of sunlight). Three different scripts were narrated by the experimenter coinciding with the selection of three different environments, specifically, a trail walk in autumn, a spring meadow, and a hillside walk in snowy winter. Instructions given to participants were as follows: “During each session, I will be guiding you through the experience by reading a script. In this part of the exercise, we will be entering into an environment via our imagination, TV screen, or VR. By reading a script, I will guide you through the environment and we will explore and experience the ability to manipulate it… All the while, please pay attention to what it is like for you to complete this exercise, so that you can describe the memory of your experiences when it is over. Pay attention to thoughts, feelings, sensations, and anything else that comes up…” The full instructions and scripts that were read verbatim are included in the Supplementary Material. Further, video recordings of a user experience of the same scripted exercise was used for the 2-D computer FORMAT of the task while for the noncomputerized FORMAT of the exercise participants listened to and imagined each of the scripts with their eyes closed. In the case of the 2-D monitor format, we displayed the videos on a wide-screen (34″ diagonal) curved monitor to increase the potential for immersion via that medium. The scripts describing each of the three different seasonal environments were randomized to the three different task formats across participants (e.g., one participant might have completed the trail walk in autumn in VR, the spring meadow walk in 2-D format, and the hillside snowy winter walk using mental imagery alone, this combination being different for another participant).

### Study 3 Results and Discussion

**FIGURE 12:**
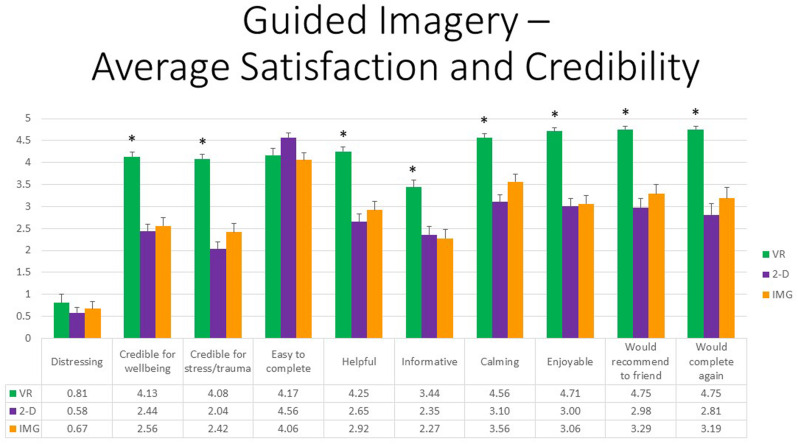
Bars illustrate the mean ratings obtained on a 0- to 5-point scale for the VR (green), 2-D (purple), and IMG (orange) formats of the task, also written in the embedded table below. Error bars illustrate the standard error of measurement. Asterisks denote the 8 of 10 ratings in which the mean rating for the VR condition significantly exceeded the mean rating for both of the 2-D and IMG conditions. Any other statistically significant pairwise comparisons are described in the text. IMG, imagery; 2-D, two-dimensional; VR, virtual reality.

First, referring to response to the 10 items of the satisfaction and credibility questionnaire, at the multivariate level, referring to within-group effects, the main effect of FORMAT was again highly statistically significant, *F*(20,27) = 7.617, *p* < 0.001, η^2^ = 0.849, whereas the effect of ORDER or the interaction between ORDER and FORMAT were not. At the univariate level, the within-group main effect of TYPE was statistically significant for all satisfaction and credibility ratings except in the case of “distressing” (*p* = 0.357). All follow-up paired *t* tests indicated that participants were more satisfied in response to the VR exercise in comparison to the 2-D computer exercise, and the same was found when comparing the VR exercise to eyes-closed imagery except that these two task formats were not reported to differ regarding how “easy to complete” they were (*p* = 0.627). Additionally, obtained effect sizes for these paired comparisons involving the VR format were again large in most instances (i.e., *d′ >* 0.80); see Supplementary Material for tabled effect sizes. Finally, eyes-closed mental imagery was regarded as less “easy to complete” (*p* = 0.005) but more “calming” (*p* = 0.049) when compared with eyes-open viewing of the scenes on the 2-D monitor. Results are shown in Figure 12.

**FIGURE 13:**
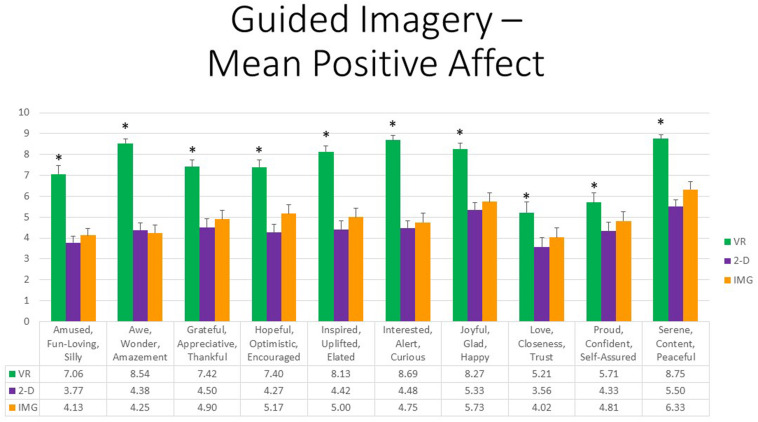
Bars illustrate the mean ratings obtained on a 0- to 10-point scale for the VR (green), 2-D (purple), and IMG (orange) formats of the task, also written in the embedded table below. Error bars illustrate the standard error of measurement. Asterisks denote the 10 of 10 ratings in which the mean rating for the VR condition significantly exceeded the mean rating for both of the 2-D and IMG conditions. Any other statistically significant pairwise comparisons are described in the text. IMG, imagery; 2-D, two-dimensional; VR, virtual reality.

Further, referring to the 10 positive affective states measured by the mDES-PA survey, at the multivariate level, the main effect of exercise FORMAT was again highly statistically significant, *F*(20,27) = 8.90, *p* < 0.001, η^2^ = 0.87, whereas the effects of ORDER and the interaction between ORDER and FORMAT were not. At the univariate level, the within-group main effect of FORMAT was statistically significant for all positive emotions without exception. Moreover, all follow-up paired *t* tests indicated that participants experienced greater positive emotion in response to the VR exercise in comparison to both the 2-D computer exercise and noncomputer IMG exercise, whereas the latter two conditions differed only in response to two ratings, “hopeful, optimistic, encouraged” and “serene, content, peaceful,” whereby the eyes-closed imagery condition received the higher rating; results are depicted in Figure 13. Again, obtained effect sizes for these paired comparisons involving the VR format were also found to be large in most instances (i.e., *d′ >* 0.80); see Supplementary Material. We therefore conclude that this guided imagery exercise produced the greatest positive affect when completed within the immersive format of VR as compared to completing it while viewing the scenes on a 2-D monitor or with one’s eyes closed via mental imagery alone.

**FIGURE 14:**
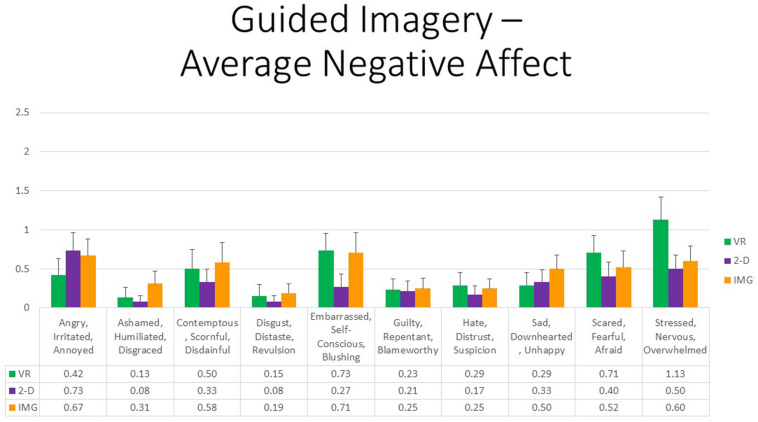
Bars illustrate the mean ratings obtained on a 0- to 10-point scale for the VR (green), 2-D (purple), and IMG (orange) formats of the task, also written in the embedded table below. Error bars illustrate the standard error of measurement. In the absence of a significant ANOVA, no pairwise comparisons were conducted but rather all are assumed to be nonsignificant. See text for further description. IMG, imagery; 2-D, two-dimensional; VR, virtual reality.

Finally, referring to the 10 negative affective states measured by the mDES-NA survey, at the multivariate level, once again no within-group main effects or interactions were statistically significant. Moreover, whereas the between-group multivariate effect of ORDER was significant, *F*(10,37) = 2.13, *p* < 0.05, η^2^ = 0.37, all follow-up univariate tests were nonsignificant, and so no between-group comparisons were undertaken. To afford comparability with the findings reported for positive affect, Figure 14 also displays results for self-reported negative affects, but again note the differences between these figures in vertical scaling. We are therefore again led to conclude that the guided imagery exercise produced little negative affect when completed in any format, and there were no significant differences in experienced negative affect between task formats.

**FIGURE 15:**
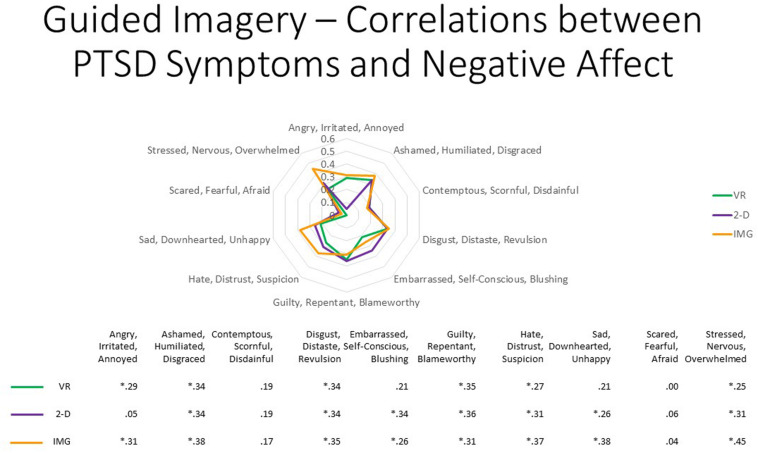
The radar figure further depicts the pattern of the correlations noted in the table below between PTSD symptoms (measured by the PCL-5), on the one hand, and the various negative affect ratings (measured by the mDES), on the other. Asterisks denote the 20 of 30 ratings in which the correlation is statistically significant at *p* < 0.05 (1-tailed). See text for further description. IMG, imagery; 2-D, two-dimensional; VR, virtual reality.

**FIGURE 16:**
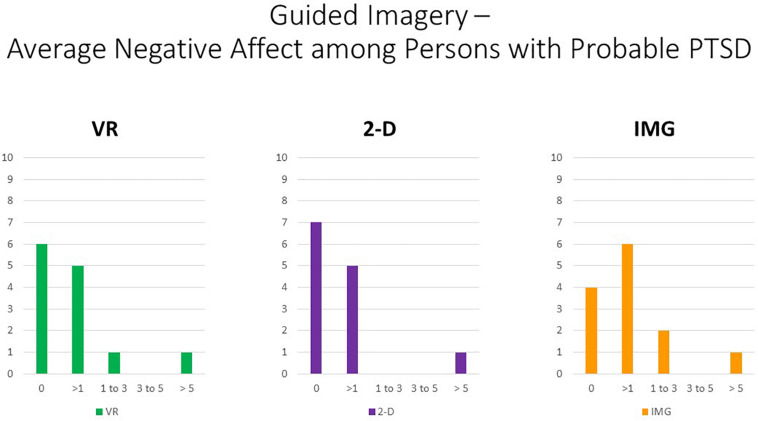
Bars illustrate the number of participants in the sample with probable PTSD (i.e., the subsample scoring above 33 on the PCL-5) whose average score across the 10 negative affect ratings on the MDES exceeded the values indicated on the *x* axis. It can be seen that very few scored above even a relatively low value of 3 on the 0- to 10-point scale for any of the task formats: VR (green), 2-D (purple), and IMG (orange). See text for further description.

Nevertheless, once again persons with increased PTSD symptoms tended to report greater negative affect in response to the task when completed in any of the three different formats for most of the different negative emotions surveyed; Pearson correlations are reported in Figure 15 and average approximately *r* ≈ 0.30. We therefore again conclude that participants with probable PTSD are likely at increased risk of responding with increased negative affect to the task regardless of the format in which it is administered. However, once again, supplementary analyses found that, even among the 13 participants who scored above the recommended cutoff of 33 for probable *DSM-5* PTSD diagnosis on the PCL-5, the majority again had an average mDES-NA score of less than one on the 0- to 10-point scale across the 10 emotional states surveyed, and only a single person had an average score of 5 or more on the 0- to 10-point scale for any format (Figure 16).

Regarding the thematic analysis of open-ended comments, we found that 14 themes were observed at least five times (i.e., >10% of participant ratings). These themes were: (1) Calming (*n* = 27), (2), vividness–realism (*n* = 27), (3) Positive Affect (*n* = 19), (4) fun–interesting (*n* = 20), (5) nausea–dizziness (*n* = 8), (6) Auditory stimuli facilitated immersion (*n* = 7), (7) Visual stimuli facilitated immersion (*n* = 6), (8) Beautiful-Vibrant Scene (*n* = 6), (9) Respite-Escape (*n* = 6), (10) Active-Engaging (*n* = 5), (11) Novel-Unique (*n* = 5), (12) Sense of Control (*n* = 5), (13) Increased intensity of emotions (*n* = 5), and (14) Preferred VR (*n* = 5). Again, themes 1, 3, and 4 were conceived as redundant with response to the mDES survey and so are not discussed further. Moreover, given the increased number of themes identified, for the sake of brevity we will only describe those themes that were identified at least six rather than only five times and provide fewer examples for less frequently observed themes.

First, referring to the previously mentioned theme of “vividness–realism,” examples included “The experience was quite realistic. I felt like I was in a forest. I could observe nature’s beauty like the deer…”; and “Felt like I was in a completely different space. Lost a sense of everything in the room—wasn’t even in a room anymore—it was crazy. It was realistic. It really felt like standing there”; and “That one was definitely more immersive, I didn’t get distracted at all…” Referring to the theme of experienced “nausea–dizziness,” however, several participants reported experiencing this, for example, “I felt dizzy in the beginning, kind of motion sickness.” This was often invoked when participants navigated the scenes using a smooth transition between frames rather than the blink teleport functionality. Participants also frequently reported the multisensory aspects of the VR experience supported its immersive effects, referring both to audition and vision, for example, “The visuals were soothing. The music and sound effects were nice too.” Several participants also described the beauty and vibrancy of the scenes, for example, “The symbol at the end; it was radiant.” Finally, several participants described the experience as providing a brief respite or escape from life stressors, for example, “It was life changing, I never expected this to be happening. I was feeling overwhelmed, I am stressed because of final exams right now, so this relieved me of the stress and took me to another world” and “I could go escape in that for sure, if I ever wanted to go take a break and walk… That was a cool experience. Liberating, escaping.”

The mean number of themes identified in participants’ comments was 4.56 (SD = 2.37). Four participants’ comments that were coded as exhibiting nine or more themes, thus being approximately two standard deviations of the mean, are reported verbatim in order to describe some of the more qualitatively rich phenomenological descriptions reported in response to this guided wilderness imagery task within VR. Specifically, the first participant, whose response was coded to have nine themes, commented:

“It felt like it was actually happening in reality. Felt peaceful, it was soothing, and it was really close to nature; the group of deer felt real and the sound of ducks and water, it felt like I had some time to myself—“me time.” Watching the plants grow was realistic and hearing the rain—it actually felt like it was raining around me. And the aura and colored lights made me feel like I was in the center of a circle, and everything around me was magical.”

Another participant whose response was coded as including nine themes described the VR experience as follows:

“I felt like I was actually on a hike. It was really real, all the visuals and sounds. I forgot that I was wearing the headset for a bit, and I forgot what the room looked like. Usually when I go for hikes I usually see all these things, but I don’t usually get to interact with them like I did today, for example, I was able to climb the boulder, which I wouldn’t do in real life. And it made you happy. It was wowing.”

A third participant, whose response was coded to represent 10 themes, commented:

“You can actually look around like you were there. More realistic, all the light and sound, as if you’re actually hearing the sounds. Soothing and maybe want to stay in that place. Helped me forget about stressful things I’m going through. I want to stay in that place. Very realistic.”

Finally, the fourth participant, whose response was coded to contain 11 themes, commented:

“It was stressful, but you feel release—especially the tree and flowers, they were so powerful and energetic and full of hope. The rain felt better and peaceful than before—raindrops—it was a very quiet and peaceful scene where no one can disturb you; you can focus on your own. Such a wonderful place.”

## General Discussion

The three studies conducted herein provide a proof of concept of an integrative therapeutic approach to the application of VR to interventions for trauma- and stressor-related disorders and improvement of mental health and well-being more broadly that we have titled *virtual reality integrative therapy*. Across instances of three different but each standard psychotherapeutic interventions (unguided imagery, autobiographical recall, and guided imagery), participants reported the greatest perceived satisfaction and credibility and experienced the greatest positive affect in response to the intervention when delivered in the perceptual, egocentric format of VR as compared to the computerized format of a standard flat-screen monitor (2-D) and the usual noncomputerized and nonperceptual (IMG) format of psychotherapy as relying on the use of mental imagery and episodic recall alone. In so far as our chief results were replicated across the three different tasks we administered, VRIT can be understood from the integrative psychotherapeutic perspective of technical eclecticism (Zarbo et al., 2016). Specifically, the practice of VRIT can be understood as involving the selection of different VR interventions to suit the presenting problems of different clients partly based on client preference. This notion of VRIT therefore expands the scope of VR in mental healthcare beyond the practice of exposure therapy (VRET) to include applications better associated with other schools of psychotherapy (e.g., cognitive, psychodynamic, experiential) consistent with the previously cited early vision of Riva (2005). However, we also would like to suggest that the experiential approach embraces the notion of VRIT as conducted here in so far as we have collected affective and phenomenological responses to each intervention; consistent with this, the experiential approach to psychotherapy has itself been considered an instance of integrative therapy (e.g., Greenberg et al., 1998). It is also interesting to point out in the context of our results that, in contrast with the longer-term results of VRET that tend to show equivalence rather than superiority of VRET over non-VR exposure therapy (Gonçalves et al., 2012; Opriş et al., 2012; Wenrui et al., 2019), the current results showed that the VRIT interventions studied here were usually found to be superior to closely matched non-VR control conditions in producing positive affect and satisfaction–credibility as interventions for trauma- and stressor-related disorders as well as improving psychological well-being more broadly. In any case, from the perspective of VRIT as espoused herein, the three tasks we evaluated are considered only a sampling of the kinds of psychotherapeutic tasks to which VR could be applied, and we look forward to the creative design of other psychotherapeutic tasks for use within VR by other clinical researchers.

**FIGURE 17:**
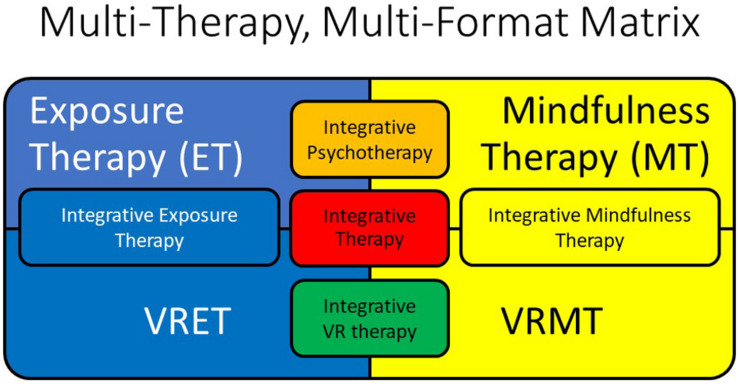
This schematic of a Venn diagram illustrates commonalities and differences between two psychological interventions, namely, exposure therapy (in blue) versus mindfulness therapy (in yellow). Commonalities between the interventions when delivered without use of VR are illustrated as the concept of integrative psychotherapy (in orange), whereas commonalities between the interventions when delivered through VR are illustrated as the concept of integrative VR therapy (in green). Commonalities between each of the independent non-VR versus VR formats of each of the independent interventions are illustrated in their respective same-colored insets. Finally, the notion of integrative therapy as commonalities across the different interventions that are in turn across the different formats of delivery is illustrated at the center of the figure (in red). See text for further description. VR, virtual reality; VRET, virtual reality exposure therapy; VRMT, virtual reality mindfulness therapy.

Comparing results obtained across our three studies and therefore between different kinds of psychotherapeutic tasks, whereas the VR format of all three interventions received high satisfaction and credibility ratings, positive affect ratings tended to be highest for the guided imagery task used in Study 3 followed by the unguided imagery task used in Study 1, while the correlation between negative affect and PTSD symptoms appeared to be highest in response to the narrative task used in Study 2 followed by the guided imagery task used in Study 3. Further, the qualitative themes most often present in participants’ open-ended comments seemed also to vary across the three different psychotherapeutic tasks administered. These results suggest that different kinds of psychological interventions may vary in their expected benefits when delivered in VR. However, we emphasize that in the current research participants were *not* randomized to the three different tasks we assessed, but rather each task was examined in three separate and consecutively conducted studies. As a result, we cannot be sure that such observations relate to effects of the three different tasks we administered or only to unmeasured variables that may also differentiate and confound our participant groups. In the future, we recommend the comparative study of two or more interventions each delivered in VR and non-VR formats as a means of identifying what may be unique to the VR medium across different interventions, what may be unique to different kinds of psychological interventions across different (i.e., VR versus non-VR) administration formats, and finally what may be unique to the interactive combination of task by format. Figure 14 provides a schematic of this research methodology in the form of a Venn diagram described as a “multitherapy, multiformat matrix” similar to the well-known approach to psychometric validation proffered by Campbell and Fiske (1959). Given in Figure 17 is the example of comparing exposure therapy to mindfulness-based therapy, the latter of which is a therapeutic orientation our group has also applied to VR in a previous study (Mistry et al., manuscript submitted for publication). We emphasize that this schematic was *not* examined in the present studies due to lack of random assignment to task types; instead, this kind of analysis is suggested for future research.

**FIGURE 18:**
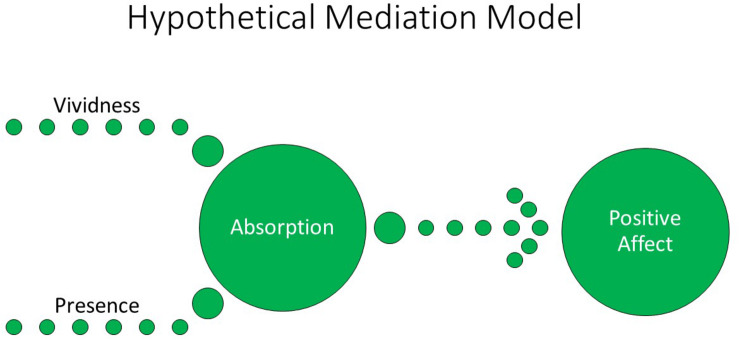
Schematic of a multiple mediation model that suggests the experience of absorption as a highly immersive state may be partially the product of the experience of vivid perception and presence (egocentricity). When stimuli are positively valenced, this schematic predicts a highly immersive experience of such stimuli should produce increased positive affect. See text for further description.

Beyond collecting affective and credibility ratings on a numeric scale, we also conducted open-ended phenomenological interviews following each task that we submitted to thematic analysis. Beyond identifying emotional responses that overlapped those already assessed by the rating scales, these interviews also clarified other phenomenological responses that were experienced frequently to the VR format of each of the tasks we administered. In particular, partly informed by the qualitative feedback we received from our participants’ open-ended experiential response to our VR tasks, we are led to speculate that what may make VR a relatively unique and particularly impactful medium for delivering psychotherapeutic interventions relates to its effects on at least two phenomenological variables, that is, vividness and presence, relating to the perceptual (versus imagery-based) and egocentric (versus nonegocentric) frames of reference afforded by different intervention formats, respectively (Figure 1). Particularly as compared to mental imagery without the aid of computers, VR may afford an increase in vividness as a perceptual modality as compared to mental imagery alone especially among persons who are characteristically limited in vividness of mental imagery (e.g., May et al., 2013). Further, especially as compared to 2-D perception of stimuli on computer flat screens, VR affords an increase in the sense of “presence” as an egocentric frame of reference into the depicted environments (e.g., Lessiter et al., 2001). Further, it would seem that, together, vividness and the sense of presence may combine to create the highly immersive experience often characteristic of VR, which, when especially pronounced, might constitute an instance of the phenomenological state of absorption (e.g., Tellegen and Atkinson, 1974; Roche and McConkey, 1990). However, it is also important to point out that neither vividness nor presence appears necessary to produce the experience of absorption, where even playing Tetris in 2-D format can also be experienced as absorbing, even though such an activity would seem to produce neither vividness or presence, implying that the experience of absorption must also be capably experienced through mechanisms beyond vividness and presence alone. Future studies should evaluate whether baseline individual differences in each of these psychological variables moderate outcomes to interventions administered in VR versus non-VR formats, and whether experienced levels of each variable partially mediate differential outcomes for affective responses and experienced satisfaction and credibility between formats. Also partly guided by qualitative feedback we received from participant responses, we hypothesize as a multiple mediation model that VR, as compared to other intervention formats, may produce increased vividness and presence, which in turn may produce increased absorption as a partial mediator of the uniqueness of VR in leading to primary intervention outcomes such as for increasing positive affect and psychological well-being; Figure 18 depicts this hypothesized process model, which could be evaluated in future studies. Moreover, both of the aforementioned primary phenomenological elements (vividness, presence) should be responsive to ongoing technical improvements in VR technology, for example, pixel resolution, field of view, and the use of multisensory stimuli. Unfortunately, a limitation of the present study is that we did not administer validated measures of experienced vividness, presence, or absorption that would have enabled evaluation of the proposed multiple mediation model in the current study, and so this task must be left for future research.

Importantly, much research has investigated VR as an effective medium for engendering the psychological experience of awe, which may also be a mediator of the effects of VR for overall positive affect. For example, Chirico and colleagues demonstrated that VR depictions of natural environments (e.g., mountains, trees, the Earth) elicited significantly stronger self-reported feelings of awe when compared to neutral emotional conditions (Chirico et al., 2016, 2017a,b), and Quesnel and Riecke (2018) showed similar results for subjective reports that were validated against an objective physiological measure of awe, that is, goose bumps on the skin. The experience of “awe, wonder, amazement” was indeed one of the most pronounced positive affective responses to the VR tasks studied here, extending these prior findings. Nevertheless, we emphasize that VR effects in generating awe may be at least partly contingent on novelty effects among first-time users that may habituate and even extinguish with repeated use. Indeed, based on the theory that provoking awe is thought to require experiencing a need for accommodation (e.g., Keltner and Haidt, 2003), repeated users of VR technology would be expected to be able to assimilate new VR experiences into episodic memory and therefore be less amenable to experiencing awe with repeated use. We therefore recommend that future researchers examine the awe-provocative effects of VR over repeated use and acknowledge that a limitation of the current study was its neglect of assessing prior experience with VR among our participants. In any case, the present conceptual framework, in its emphasis on producing high-intensity positive affective responses such as awe as well as other emotions, fits with the notion of VR as a potentially effective medium for engendering transformative experiences (Riva et al., 2016). We thus look forward to the design of VR experiences not only for engendering awe but also other equally profound alterations in consciousness and self-transcendence such as intense absorption, ecstasy (e.g., rapture, bliss), reverence (e.g., sacred, hallowed), loving-kindness and equanimity, and other nondual states (e.g., Yaden et al., 2017; Hanley et al., 2018) that were unfortunately not measured in the studies described here.

It is important to point out that persons with high PTSD symptoms reported more distress in response to all of the tasks administered in all of the formats we studied them, that is, also including in the VR format. Nevertheless, supplementary follow-up analyses showed that, even among those with high PTSD symptoms, self-reported distress in the present sample remained low in intensity on the 0- to 10-point scale we administered, with only a single participant reporting that they experienced distress with moderate intensity in response to any of the therapeutic tasks we evaluated. In any case, we recommend all VRIT tasks be administered using a trauma-informed care approach that emphasizes safety, trust, choice, collaboration, and empowerment (Harris and Fallot, 2001; Fallot and Harris, 2008; Classen and Clark, 2017).

The present research is described only as an initial proof-of-concept of VRIT due to several inherent limitations. First, our interventions were evaluated only during a single session; we did not assess participants’ prior use and familiarity with VR technology, and the long-term effects of these interventions when administered via VR are unknown. Moreover, participants in our studies were restricted with respect to age range and educational levels, and their traumatic and stressful life event history and psychological symptoms were only assessed by self-report rather than clinical interview. Further, thematic coding was not blinded to the format of the task in which the responses referred. Moreover, although no adverse events were noted in response to our procedures, it is a limitation that we did not screen for neurocognitive disorders that could be risk factors for participation in VR such as epilepsy and severe motion sickness and that we did not measure motion sickness as a response to the tasks we administered using a validated measure. These concerns will need to be addressed in future research studies that replicate and extend the present work. Further, cost-effectiveness analyses of administering these psychological interventions in differing formats should also be undertaken in the future. We may simply note that at present to administer any of the three interventions studied herein would require the use of a computer with a suitable graphics card (≈US $1,500), a suitable HMD (≈US $500), and the respective software applications (≈ US $20).

We look forward to trials of other psychotherapeutic tasks within VR as may be developed by other clinicians and researchers. We hope that, through the integrative, eclectic notion of VRIT, psychotherapists may creatively explore the use of VR technology to deliver vivid, egocentric, and immersive therapeutic experiences that bring about clinically significant and transformative change in persons experiencing trauma- and stressor-related disorders and other mental health problems. Following Riva (2005), we also envision VR as increasingly part of the future of clinical psychology and think that it is critical that psychotherapists explore its clinical utility broadly.

## Data Availability Statement

The datasets generated for this study are available on request to the corresponding author.

## Ethics Statement

The studies involving human participants were reviewed and approved by Western University Research Ethics Board. The patients/participants provided their written informed consent to participate in this study.

## Author Contributions

All authors listed have made a substantial, direct and intellectual contribution to the work, and approved it for publication.

## Conflict of Interest

The authors declare that the research was conducted in the absence of any commercial or financial relationships that could be construed as a potential conflict of interest.

## Footnotes

1 https://www.samsung.com/us/support/owners/product/hmd-odyssey-plusmixed-reality

2 https://www.esrb.org/ratings-guide/

3 https://www.tiltbrush.com/

4 https://www.microsoft.com/en-us/p/paint-3d/9nblggh5fv99?activetab=pivot:overviewtab

5 https://arvr.google.com/earth/

6 https://www.google.com/earth/

7 https://naturetreksvr.com/

## References

[B1] Anderson C. L. Monroy M. Keltner D. (2018). Awe in nature heals: evidence from military veterans, at-risk youth, and college students. *Emotion* 18 1195–1202. 10.1037/emo0000442 29927260

[B2] Andrade J. May J. Deeprose C. Baugh S. Ganis G. (2013). Assessing vividness of mental imagery: the plymouth sensory imagery questionnaire. *Br. J. Psychol.* 105 547–563. 10.1111/bjop.12050 24117327

[B3] Avant E. Swopes R. Davis J. Elhai J. (2011). Psychological abuse and posttraumatic stress symptoms in college students. *J. Int. Viol.* 26 3080–3097. 10.1177/0886260510390954 21156683

[B4] Ballou M. (1995). *Psychological Interventions: A Guide to Strategies.* Westport, CT: Praeger Publishers.

[B5] Bettmann J. E. Gillis H. L. Speelman E. A. Parry K. J. Case J. M. (2016). A meta-analysis of wilderness therapy outcomes for private pay clients. *J. Child Fam. Stud.* 25 2659–2673. 10.1007/s10826-016-0439-0

[B6] Bettmann J. E. Lundahl B. W. Wright R. Jasperson R. A. McRoberts C. H. (2011). Who are they? A descriptive study of adolescents in wilderness and residential programs. *Resident. Treat. Child. Youth* 28 192–210. 10.1080/0886571X.2011.596735

[B7] Blevins C. A. Weathers F. W. Davis M. T. Witte T. K. Domino J. L. (2015). The posttraumatic stress disorder checklist for DSM-5 (PCL-5): development and initial psychometric evaluation. *J. Trauma Stress* 28 489–498. 10.1002/jts.22059 26606250

[B8] Campbell D. T. Fiske D. W. (1959). Convergent and discriminant validation by the multitrait-multimethod matrix. *Psychol. Bull.* 56 81–105. 10.1037/h0046016 13634291

[B9] Chirico A. Cipresso P. Yaden D. B. Biassoni F. Riva G. Gaggioli A. (2017a). Effectiveness of immersive videos in inducing awe: an experimental study. *Sci. Rep.* 7:1218. 10.1038/s41598-017-01242-0 28450730 PMC5430774

[B10] Chirico A. Ferrise F. Cordella L. Gaggioli A. (2017b). Designing awe in virtual reality: an experimental study. *Front. Psychol.* 8:2351. 10.3389/fpsyg.2017.02351 29403409 PMC5786556

[B11] Chirico A. Yaden D. B. Riva G. Gaggioli A. (2016). The potential of virtual reality for the investigation of awe. *Front. Psychol.* 7:1766. 10.3389/fpsyg.2016.01766 27881970 PMC5101419

[B12] Classen C. C. Clark C. S. (2017). “Trauma-informed care,” in *APA Handbooks in Psychology^®^. APA Handbook of Trauma Psychology: Trauma Practice*, ed. Gold S. N. (Washington, DC: American Psychological Association), 515–541. 10.1037/0000020-025

[B13] Cloitre M. Stovall-McClough K. C. Miranda R. Chemtob C. M. (2004). Therapeutic alliance, negative mood regulation, and treatment outcome in child abuse-related posttraumatic stress disorder. *J. Consult. Clin. Psychol.* 72 411–416. 10.1037/0022-006X.72.3.411 15279525

[B14] Cohen B. M. Barnes M. Rankin A. B. (1995). *Managing Traumatic Stress Through Art: Drawing From the Center.* Baltimore, MD: The Sidran Press.

[B15] Conway M. A. Pleydell-Pearce C. W. (2000). The construction of autobiographical memories in the self-memory system. *Psychol. Rev.* 107 261–288. 10.1037/0033-295X.107.2.261 10789197

[B16] Crawford M. J. Killaspy H. Barnes T. R. Barrett B. Byford S. Clayton K. (2012). Group art therapy as an adjunctive treatment for people with schizophrenia: multicentre pragmatic randomised trial (MATISSE). *Br. Med. J.* 344 1–9. 10.1136/bmj.e846 22374932 PMC3289714

[B17] Credé M. Niehorster S. (2012). Adjustment to college as measured by the student adaptation to college questionnaire: a quantitative review of its structure and relationships with correlates and consequences. *Educ. Psychol. Rev.* 24 133–165. 10.12691/rpbs-3-2-1

[B18] Decker K. P. Deaver S. P. Abbey V. Campbell M. Turpin C. (2018). Quantitatively improved treatment outcomes for combat-associated PTSD with adjunctive art therapy: randomized controlled trial. *Art Ther.* 35 184–194. 10.1080/07421656.2018.1540822

[B19] Deng W. Hu D. Xu S. Liu X. Zhao J. Chen Q. (2019). The efficacy of virtual reality exposure therapy for PTSD symptoms: a systematic review and meta-analysis. *J. Affect. Disord.* 257 690–709. 10.1016/j.jad.2019.07.086 31382122

[B20] Ehring T. Quack D. (2010). Emotion regulation difficulties in trauma survivors: the role of trauma type and PTSD symptom severity. *Behav. Ther.* 41 587–598. 10.1016/j.beth.2010.04.004 21035621

[B21] Fallot R. D. Harris M. (2008). Trauma-informed approaches to systems of care. *Trauma Psychol. Newslett.* 3:6.

[B22] Felitti V. J. Anda R. F. Nordenberg D. Williamson D. F. Spitz A. M. Edwards V. (1998). Relationship of childhood abuse and household dysfunction to many of the leading cause of death on adults: the Adverse childhood Experiences (ACE) study. *Am. J. Prevent. Med.* 14 245–258. 10.1016/S0749-3797(98)00017-8 9635069

[B23] Foa E. B. Kozak M. J. (1986). Emotional processing of fear: exposure to corrective information. *Psychol. Bull.* 99 20–35. 10.1037/0033-2909.99.1.20 2871574

[B24] Fredrickson B. L. Tugade M. M. Waugh C. E. Larkin G. R. (2003). What good are positive emotions in crisis? A Prospective study of resilience and emotions following the terrorist attacks on the United States on September 11th, 2001. *J. Pers. Soc. Psychol.* 84 365–376. 10.1037/0022-3514.84.2.365 12585810 PMC2755263

[B25] Frewen P. Hargraves H. DePierro J. D’Andrea W. Flodrowski L. (2016). Meditation Breath Attention Scores (MBAS): development and investigation of an internet-based assessment of focused attention during meditation practice. *Psychol. Assess.* 28 830–840. 10.1037/pas0000283 27078182

[B26] Frewen P. Rogers N. Flodrowski L. Lanius R. (2015). Mindfulness and metta-based trauma therapy (MMTT): initial development and proof-of-concept of an internet resource. *Mindfulness* 6 1322–1334. 10.1007/s12671-015-0402-y 26609330 PMC4646922

[B27] Frewen P. A. Lanius R. A. (2014). Trauma-related altered states of consciousness: exploring the 4-D model. *J. Trauma Dissoc.* 15 436–456. 10.1080/15299732.2013.873377 24650122 PMC4440663

[B28] Gaggioli A. A. Ferscha A. Riva G. Dunne S. (2016). *Human Computer Confluence. Transforming Human Experience Through Symbiotic Technologies.* Berlin: De Gruyter.

[B29] Gantt L. Tinnin L. W. (2009). Support for a neurobiological view of trauma with implications for art therapy. *Arts Psychother.* 36 148–153. 10.1016/j.aip.2008.12.005

[B30] Gillis H. Speelman E. Linville N. Bailey E. Kalle A. Oglesbee N. (2016). Meta-analysis of treatment outcomes measured by the Y-OQ and Y-OQ-SR comparing wilderness and non-wilderness treatment programs. *Child Youth Care Forum* 45 851–863. 10.1007/s10566-016-9360-3

[B31] Gonçalves R. Pedrozo A. L. Coutinho E. S. F. Figueira I. Ventura P. (2012). Efficacy of virtual reality exposure therapy in the treatment of PTSD: a systematic review. *PLoS One* 7:e48469. 10.1371/journal.pone.0048469 23300515 PMC3531396

[B32] Greenberg L. S. Wastson J. C. Lietaer G. (1998). *Handbook of Experiential Psychotherapy.* New York, NY: The Guilford Press.

[B33] Haeyen S. van Hooren S. van der Veld William M. Hutschemaekers G. (2018). Promoting mental health versus reducing mental illness in art therapy with patients with personality disorders: a quantitative study. *Arts Psychother.* 58 11–16. 10.1016/j.aip.2017.12.009

[B34] Hanley A. W. Nakamura Y. Garland E. L. (2018). The Nondual Awareness Dimensional Assessment (NADA): new tools to assess nondual traits and states of consciousness occurring within and beyond the context of meditation. *Psychol. Asssess.* 30 1625–1639. 10.1037/pas0000615 30058824 PMC6265073

[B35] Harris M. Fallot R. (2001). *Using Trauma Theory to Design Service Systems.* San Francisco, CA: Josseey-Bass.

[B36] Hass-Cohen N. Bokoch R. Clyde Findlay J. Banford Witting A. (2018). A four-drawing art therapy trauma and resiliency protocol study. *Arts Psychother.* 61 44–56. 10.1016/j.aip.2018.02.003

[B37] Henderson P. Rosen D. Mascaro N. (2007). Empirical study on the healing nature of mandalas. *Psychol. Aesthet. Creat. Arts* 1 148–154. 10.1037/1931-3896.1.3.148

[B38] Holmes E. A. Blackwell S. E. Burnett Heyes S. Renner F. Raes F. (2016). Mental imagery in depression: phenomenology, potential mechanisms, and treatment implications. *Ann. Rev. Clin. Psychol.* 12 249–280. 10.1146/annurev-clinpsy-021815-092925 26772205

[B39] Hunt J. Eisenberg D. (2010). Mental Health Problems and help-seeking behavior among college students. *J. Adolesc. Health* 46 3–10. 10.1016/j.jadohealth.2009.08.008 20123251

[B40] Ji J. Kavanagh D. Holmes E. MacLeod C. Di Simplicio M. (2019). Mental imagery in psychiatry: conceptual & clinical implications. *CNS Spectr.* 24 114–126. 10.1017/S1092852918001487 30688194

[B41] Johnson N. Johnson D. (2013). Factors influencing the relationship between sexual trauma and risky sexual behavior in college students. *J. Interpers. Viol.* 28 2315–2331. 10.1177/0886260512475318 23400885

[B42] Jubb H. (2017). The effectiveness of self-soothing techniques for people with PTSD in secure units. *Mental Health Pract.* 20 28–32. 10.7748/mhp.2017.e1142

[B43] Keltner D. Haidt J. (2003). Approaching awe, a moral, spiritual, and aesthetic emotion. *Cogn. Emot.* 17 297–314. 10.1080/02699930302297 29715721

[B44] Kroenke K. Spitzer R. L. Williams J. B. Löwe B. (2009). An ultra-brief screening scale for anxiety and depression: the PHQ-4. *Psychosomatics* 50 613–621. 10.1176/appi.psy.50.6.613 19996233

[B45] Lessiter J. Freeman J. Keogh E. Davidoff J. (2001). A cross-media presence questionnaire: the ITC-sense of presence inventory. *Presence* 10 282–297. 10.1162/105474601300343612

[B46] Lopes R. T. Gonçalves M. M. Fassnacht D. B. Machado P. P. P. Sousa I. (2014a). Long-term effects of psychotherapy on moderate depression: a comparative study of narrative therapy and cognitive-behavioral therapy. *J. Affect. Disord.* 167 64–73. 10.1016/j.jad.2014.05.042 25082116

[B47] Lopes R. T. Gonçalves M. M. Machado P. P. P. Sinai D. Bento T. Salgado J. (2014b). Narrative therapy vs. cognitive-behavioral therapy for moderate depression: empirical evidence from a controlled clinical trial. *Psychother. Res.* 24 662–674. 10.1080/10503307.2013.874052 24479576

[B48] Lyshak-Stelzer F. Singer P. Patricia S. J. Chemtob C. M. (2007). Art therapy for adolescents with posttraumatic stress disorder symptoms: a pilot study. *Art Ther.* 24 163–169. 10.1080/07421656.2007.10129474

[B49] Martin S. (2019). *Virtual Reality Might be the Next Big Thing for Mental Health.* Berlin: Scientific American.

[B50] McPherson J. (2012). Does narrative exposure therapy reduce PTSD in survivors of mass violence? *Res. Soc. Work Pract.* 22 29–42. 10.1177/1049731511414147

[B51] Morgan K. E. White P. R. (2003). The functions of art-making in CISD with children and youth. *Int. J. Emerg. Mental Health* 5 61–76. 12882092

[B52] Neuner F. Schauer M. Roth W. T. Elbert T. (2002). A narrative exposure treatment as intervention in a refugee camp: a case report. *Behav. Cogn. Psychother.* 30 205–210. 10.1017/S1352465802002072

[B53] Nielsen F. Isobel S. Starling J. (2019). Evaluating the use of responsive art therapy in an inpatient child and adolescent mental health services unit. *Aust. Psychiatry* 27 165–170. 10.1177/1039856218822745 30652940

[B54] Norton C. L. Tucker A. Farnham-Stratton M. Borroel F. Pelletier A. (2019). Family enrichment adventure therapy: a mixed methods study examining the impact of trauma-informed adventure therapy on children and families affected by abuse. *J. Child Adolesc. Trauma* 12 85–95. 10.1007/s40653-017-0133-4 32318182 PMC7163833

[B55] Opriş D. Pintea S. García-Palacios A. Botella C. Szamosközi Ş David D. (2012). Virtual reality exposure therapy in anxiety disorders: a quantitative meta-analysis. *Depress. Anxiety* 29 85–93. 10.1002/da.20910 22065564

[B56] Pearson J. Kosslyn S. M. (2015). The heterogeneity of mental representation: ending the imagery debate. *Proc. Natl. Acad. Sci. U.S.A.* 112 10089–10092. 10.1073/pnas.1504933112 26175024 PMC4547292

[B57] Pearson J. Naselaris T. Holmes E. A. Kosslyn S. M. (2015). Mental imagery: functional mechanisms and clinical applications. *Trends Cogn. Sci.* 19 590–602. 10.1016/j.tics.2015.08.003 26412097 PMC4595480

[B58] Quesnel D. Riecke B. E. (2018). Are you awed yet? How virtual reality gives us awe and goose bumps. *Front. Psychol.* 9:2158. 10.3389/fpsyg.2018.02158 30473673 PMC6237834

[B59] Rankin A. B. Taucher L. C. (2003). A task-oriented approach to art therapy in trauma treatment. *Art Ther.* 20 138–147. 10.1080/07421656.2003.10129570

[B60] Read J. Ouimette P. White J. Colder C. Farrow S. Read J. (2011). Rates of DSM–IV–TR trauma exposure and posttraumatic stress disorder among newly matriculated college students. *Psychol. Trauma* 3 148–156. 10.1037/a0021260 25621098 PMC4301258

[B61] Riva G. (2002). Virtual reality for health care: the status of research. *Cyberpsychol. Behav.* 5 219–225. 10.1089/109493102760147213 12123244

[B62] Riva G. (2005). Virtual reality in psychotherapy: review. *CyberPsychol. Behav.* 8 220–230. 10.1089/cpb.2005.8.220 15971972

[B63] Riva G. Baños R. M. Botella C. Mantovani F. Gaggioli A. (2016). Transforming experience: the potential of augmented reality and virtual reality for enhancing personal and clinical change. *Front. Psychiatry* 7:164. 10.3389/fpsyt.2016.00164 27746747 PMC5043228

[B64] Robjant K. Fazel M. (2010). The emerging evidence for narrative exposure therapy: a review. *Clin. Psychol. Rev.* 30 1030–1039. 10.1016/j.cpr.2010.07.004 20832922

[B65] Roche S. M. McConkey K. M. (1990). Absorption: nature, assessment, and correlates. *J. Pers. Soc. Psychol.* 59 91–101. 10.1037/0022-3514.59.1.91

[B66] Russell K. C. Gillis H. Kivlighan D. (2017). Process factors explaining psycho-social outcomes in adventure. *Psychotherapy* 54 273–280. 10.1037/pst0000131 28922006

[B67] Russell K. C. Phillips-Miller D. (2002). Perspectives on the wilderness therapy process and its relation to outcome. *Child Youth Care Forum* 31 415–437. 10.1023/A:1021110417119

[B68] Sarason I. G. Johnson J. H. Siegel J. M. (1978). Assessing the impact of life changes: development of the Life Experiences Survey. *J. Consult. Clin. Psychol.* 46 932–946. 10.1037/0022-006X.46.5.932 701572

[B69] Singer J. Blagov P. Berry M. Oost K. (2013). Self-defining memories, scripts, and the life story: narrative identity in personality and psychotherapy. *J. Pers.* 81 569–582. 10.1111/jopy.12005 22925032

[B70] Smeijsters H. (2008). *Handboek Creatieve Therapie.* Bussum: Coutinho.

[B71] Sutherland K. Bryant R. (2005). Self-defining memories in post-traumatic stress disorder. *Br. J. Clin. Psychol.* 44 591–598. 10.1348/014466505X64081 16368036

[B72] Tellegen A. Atkinson G. (1974). Openness to absorbing and self-altering experiences (“absorption”), a trait related to hypnotic susceptibility. *J. Abnorm. Psychol.* 83 268–277. 10.1037/h0036681 4844914

[B73] Temming M. *Virtual Reality Therapy Has Real-Life Benefits for Some Mental Disorders.* Berlin: Science News.

[B74] Tucker A. R. Norton C. L. (2013). The use of adventure therapy techniques by clinical social workers: implications for practice and training. *Clin. Soc. Work J.* 41 333–343. 10.1007/s10615-012-0411-4

[B75] van Emmerik A. A. P. Reijntjes A. Kamphuis J. H. (2013). Writing therapy for posttraumatic stress: a meta-analysis. *Psychother. Psychosom.* 82 82–88. 10.1159/000343131 23295550

[B76] Van Lith T. (2015). Art making as a mental health recovery tool for change and coping. *Art Ther.* 32 5–12. 10.1080/07421656.2015.992826

[B77] Van Lith T. (2016). Art therapy in mental health: a systematic review of approaches and practices. *Arts Psychother.* 47 9–22. 10.1016/j.aip.2015.09.003

[B78] Virtual Reality (2019). *In Oxford Online Dictionary.* Berlin: Springer.

[B79] Weathers F. W. Litz B. T. Keane T. M. Palmieri P. A. Marx B. P. Schnurr P. P. (2013). *The PTSD Checklist for DSM–5 (PCL-5).* Boston, MA: National Center for PTSD.

[B80] Weir K. (2018). Virtual reality expands its reach. *Monit. Psychol.* 49:52.

[B81] Wertheim-Cahen T. (1991). *Getekend Bestaan: Creatieve Therapie Met Oorlogsgetroffenen.* London: ICODO.

[B82] White M. Epston D. (1989). *Literate Means to Therapeutic Ends.* Adelaide SA: Dulwich Centre Publications.

[B83] Wright J. (2009). Self-soothing - A recursive intrapsychic and relational process: the contribution of the Bowen theory to the process of self-soothing. *Aust. New Zealand J. Fam. Ther.* 30 29–41. 10.1375/anft.30.1.29 17045519

[B84] Yaden D. B. Haidt J. Hood R. W. Vago D. R. Newberg A. B. (2017). The varieties of self-transcendent experience. *Rev. Gen. Psychol.* 21 143–160. 10.1037/gpr0000102

[B85] Zang Y. Hunt N. Cox T. (2013). A randomised controlled pilot study: the effectiveness of narrative exposure therapy with adult survivors of the Sichuan earthquake. *BMC Psychiatry* 13:41. 10.1186/1471-244X-13-41 23363689 PMC3570314

[B86] Zarbo C. Tasca G. A. Cattafi F. Compare A. (2016). Integrative psychotherapy works. *Front. Psychol.* 6:2021. 10.3389/fpsyg.2015.02021 26793143 PMC4707273

