# Supplementary material for: Proof of Concept of an Eclectic, Integrative Therapeutic Approach to Mental Health and Well-Being Through Virtual Reality Technology
Source: Front Psychol. 2020 Jun 5;11:858. doi: 10.3389/fpsyg.2020.00858 (PMC7290015; doi:10.3389/fpsyg.2020.00858)
Supplement: Supplementary file 1 [file Table_1.DOCX]

**Supplementary File to:**

**Proof of concept of Virtual Reality Integrative Therapy for Mental Health and Wellbeing**

**Additional Description of Methods**

**Psychometric Measures**

**Pre-Task Surveys**

***Life Experiences Survey (LES).*** Participant exposure to 47 non-traumatic life stressors encountered during adulthood (i.e., 18 years of age and older) was assessed using the LES (Sarason, Johnson, and Siegel, 1978). This self-report questionnaire assessed life events including “marriage”, “detention in jail or comparable institution” and “death of spouse”. Items were using a 0-2 scale with response options “No”, “Yes, at least once”, and “Yes, many times”.

***Life Events Checklist for DSM-5 (LEC-5).*** Participant experience of 17 different potentially traumatic events experienced during adulthood (i.e., 18 years of age and older) was assessed using the LEC-5 (Weathers et al., 2013). Sample items from this measure include “serious accident at work, home, or during recreational activity?” and “natural disaster (for example, flood, hurricane, tornado, earthquake)”. Response options were the same as those used for the LES.

***Adverse Childhood Experiences Questionnaire.*** This questionnaire was used to assess 10 different categories of caregiver dysfunction, abuse, and neglect experienced during childhood (i.e., before 18 years of age) (Felitti et al., 1998). Participants responded to items such as “When you were a child, did you often or very often feel that no one in your family loved you or thought you were important or special? Or your family did not look out for each other, feel close to each other, or support each other?”. Responses were collected using the same 0-2 scale as for the LES and LEC-5.

***Posttraumatic Stress Disorder Checklist for DSM-5 (PCL-5).*** Participant experience of PTSD symptoms within the last month were assessed using the 20-item PCL-5 (Blevins et al., 2015). A sample item includes “Repeated, disturbing dreams of a stressful experience.” Participants used a 5-point Likert scale (0 = “*Not at all*”, 1 = “*A little bit*”, 2 = “*Moderately*”, 3 = “*Quite a bit*”, and 4 = “*Extremely*”). The present study utilized the recommended cut-off score of at least 33 as being indicative of probable PTSD (Weathers et al., 2013).

***Trauma Related Altered States of Consciousness (TRASC) Items*.** Participants’ dissociative experiences were measured using the 10-item TRASC (Frewen and Lanius 2015; Frewen et al. 2016a). Participants used the same 0-4 scale as the PCL-5 to respond to items such as “Out of Body Experience - Feeling detached or separated from your body, for example, feeling like you are looking down on yourself from above, or like you are an outside observer of your own body”. Together with a ≥ 33 score on the PCL-5, a score of at least 3 on at least one of the two aforementioned items was described by Frewen et al. (2015) was potentially indicative of the dissociative subtype of PTSD.

**Post-Task Surveys**

***Satisfaction and Credibility Questionnaire*.** Ten questions were also included to measure participant satisfaction with and perceived credibility of each meditation as an intervention for mental health disorders and increasing general wellbeing (as used by Frewen, Rogers, Flodrowski, & Lanius, 2015), which was further an adaptation of the “Credibility/Expectancy questionnaire” developed by Devilly and Borkovec (2004). Participants rated their agreement with 10 items such as “Credible as a way to improve self-regulation and enhance well-being” on a 0-5 scale (0 = “Not at all” and 5 = “Extremely”); responses to the questionnaire were examined at the item level.

***Modified Differential Emotions Scale (mDES)*.** This 20-item scale was used to measure the degree of positive affect and negative affect that participants experienced during each meditation (developed by Fredrickson, Tugade, Waugh & Larkin, 2003). Participants rated their experience of 10 positive affect items such as “Grateful, appreciative, thankful” and “Inspired, uplifted, elevated”, and 10 negative affect items such as “Sad, downhearted, unhappy” and “Stressed, nervous, overwhelmed”. Items were scored using an 11-point scale (0 = “Yes, very much more than usual” and 10 = “No, not more than usual”) to compare response to the VRIT tasks relative to normative experience. Although the mDES is typically scored for overall positive and negative affect as an average or sum of participant responses to individual items, the present studies had an interest in determining response to the VR and non-VR tasks for specific emotional states and therefore scored responses to the mDES between different task formats at the item level while restricting risk of type-1 error associated with multiple exploratory comparisons by employing multivariate ANOVA.
