# Supplementary material for: Proof of Concept of an Eclectic, Integrative Therapeutic Approach to Mental Health and Well-Being Through Virtual Reality Technology
Source: Front Psychol. 2020 Jun 5;11:858. doi: 10.3389/fpsyg.2020.00858 (PMC7290015; doi:10.3389/fpsyg.2020.00858)
Supplement: Supplementary file 2 [file Table_2.DOCX]

**Legend:** blue **=** imagination; green = 2D; red = VR

**White Winter**

We will now imagine a winter forest (We are now entering the white winter) (With your index finger, hit and hold the trigger to enter the winter forest).

To start off, calmly take in your surroundings, taking a moment to look around yourself in all directions, including upward into the sky, and downward at the ground. Notice the rabbits nearby on the ground, and the eagles flying overhead.

To your right, you can see some stairs carved out of a rock face; walk toward the stairs and climb them. when you reach the top of stairs, gaze down at all the beauty of the winter forest.

Now walk down the hill to your left and eventually you will see a group of pine trees and a bare bush – walk through them and continue down the hill. Stop here.

Now, if you turn to your left, you will see some boulders at the top of the hill. Walk up the hill toward the boulder on the right. As you approach it, look to its right and you will see a slope, walk down it toward what looks like a treed path. Continue down it and if you stop and look around, you may see a bear or a snow leopard; they are safe, you can watch them and they will not bother you.

Now, continue down the treed path until you reach the end and then to the snowbank behind it. (Use your right trigger to teleport to the furthest tree. Teleport again to the snowbank behind it.) You are now at the foot of the mountain range.

Climb the snowbank, and walk along it to your right, keeping the mountain side to your left. Continue until you are off the snowbank and see some snow drift up the mountainside. Climb up the snowdrift on the mountain to get onto the next snowbank to your right; then continue to its end and stop there. Now, imagine that it begins to snow. Now, watch as it begins to snow. Now, make it begin to snow, by reaching for the cloud symbol.

Here, imagine some time has passed, and it becomes darker as the evening falls (to reflect this, make it darker, by reaching for the moon symbol). In the darkness, you can see the glorious northern lights in the sky. Now, plant some white snow flowers (Now, watch as some white flowers are planted) (by grasping and then gently tossing the flower symbol) and notice how they glow under the starry sky.

Now, it is time to reveal your aura or spirit (by reaching for the symbol of a lotus flower). In the form of a mandala, imagine and focus on the bright and beautiful aura emanating from within you, above you, and below you, surrounding you as it reaches out far into the forest. Take one last look at this beautiful forest and your aura, paying attention to what you are experiencing, what it feels like, so that you can describe the memory of it in a few moments. Now, it is time to leave this place; you can take the memory of it with you, but now it’s time to go home.

**Legend:** blue **=** imagination; green = 2D; red = VR

**Green Meadows (Spring)**

Imagine entering a green meadow. We are now entering the green meadows. With your index finger, hit and hold the trigger to enter the green meadows.

To start off, calmly take in your surroundings, taking a moment to look around yourself in all directions, including upward into the sky, and down at the ground. Looking around you notice the many rabbits hopping amongst each other and through the tall grasses. Notice also the snowcapped mountains in the distance to your left.

Now, walk through the meadow in the direction of the mountains, between two large boulders, and down the slowly sloping hill. You will also see a gentle river further off in the distance. As you approach another boulder on your left with the tall tree beside it, you will see a family of deer; they are safe, you can watch them, or go closer if you want to, as they will not run away.

Now continue walking further down the hill toward the river on your left. You will start to hear the babbling of the brook, and a family of ducks in the water. Walk up close to the riverbank and up onto the rock, and take a moment to watch the swimming ducks.

Moving back toward the riverbank, continue along the river, keeping it your left. Keep walking for a while until you come to a wooden bridge. The bridge is much further up, so use this time to really take in the beauty of your surroundings. Admire the mountain range far in the distance, the soothing clouds in the sky, and the bright sun shining down on you.

Now that you have reached the bridge, stand in the middle of it. Imagine releasing some butterflies into the sky and watch as they fly up, higher and higher and then fly away. Watch as some butterflies are released into the sky. Watch as the symbol flies up, higher and higher, until it releases the butterflies and they fly away. Release some butterflies into the sky by reaching for the butterfly symbol and gently tossing it in into the air. Watch as the symbol flies up, higher and higher, until it releases the butterflies and they fly away.

Now, continue across the bridge where there is shade from the sun created by all the trees. Here, take a moment to plant some flowers, to watch as some flowers are planted, to plant some flowers by grasping and then gently tossing the flower symbol. Take in the beauty of the flowers as they instantly grow and come to life.

Imagine some time has passed and it begins to rain and make it begin to rain by clicking on the rain cloud symbol. Immerse yourself in the feeling and sound of the rain.

Now, it is time to reveal your aura or spirit (by reaching for the symbol of a lotus flower). In the form of a mandala, focus now on the bright and beautiful aura emanating from within you, above you, and below you, surrounding you as it reaches out far into the meadow. Take one last look at this beautiful meadow and your aura, paying attention to what you are experiencing, what it feels like, so that you can describe the memory of it in a moment. Now, it is time to leave this place; you can take the memory of it with you, but now it’s time to go home.

**Legend:** blue **=** imagination; green = 2D; red = VR

**Red Fall (Autumn)**

We will now imagine entering red fall woods. We are now entering the red fall. With your index finger, hit and hold the trigger to enter the red fall.

To start off, calmly take in your surroundings, taking a moment to look around yourself in all directions, including upward into the sky, and downward at the ground. Notice the treed path, the hills in the distance, and the river on your right. Watch as leaves of the trees fall gracefully to the ground. Notice that a small red fox is nearby; it is safe, you can watch where it goes. In the river to your right there is a duck; listen to its quacking.

Now, walk along the path with the calm river on your right. Ahead, you will come to a fork in the river, where you will see some large rocks in the water that create a path. Use these rocks to walk to the center of the water, and take in the vast scenery around you. If you look down, you will see little fish swimming around you. Now, turn around and go back to the riverbank where you will be facing a steep hill. Climb the hill to the top and once you are there, gaze down at all the beauty of the forest. Take it all in.

Take a moment and imagine planting and growing some flowers, to watch as some flowers are planted, to plant and grow some flowers by reaching for the daisy flower symbol; take in the beauty of the flowers as they instantly come to life and grow. Now imagine the release of some butterflies into the sky and watch as they fly up, higher and higher. Now watch as some butterflies are released into the sky. Watch as the symbol flies up, higher and higher until the butterflies are released. Now, release a butterfly into the sky by reaching for the butterfly symbol and gently tossing it into the sky; watch as it flies up, higher and higher until the butterflies are released.

In the distance in front of you, you can see a large boulder, where the eagles fly by and sometimes land. Go down the hill, around to the right of the boulder and climb it.

Now that you are on the boulder, once again take in the scenery. Wait for an eagle to land for a moment on the boulder and watch it take off again.

Now, imagine some time has passed – to reflect this, imagine it getting darker, the sky becomes darker, and it starts to rain. To reflect this, make it darker and start to rain by reaching and clicking on the rainy cloud symbol. Immerse yourself in the feeling and sound of the rain in the darker evening.

Finally, it is time to reveal your aura or spirit by reaching for the symbol of a lotus flower. In the form of a mandala, focus now on the bright and beautiful aura emanating from within you, above you, and below you, surrounding you as it reaches out far into the forest. Take one last look of this beautiful forest and your aura, paying attention to what you are experiencing, what it feels like, so that you can describe the memory of it in a moment. Now, it is time to leave this place; you can take the memory of it with you, but it’s time to go home.
