# Supplementary material for: Proof of Concept of an Eclectic, Integrative Therapeutic Approach to Mental Health and Well-Being Through Virtual Reality Technology
Source: Front Psychol. 2020 Jun 5;11:858. doi: 10.3389/fpsyg.2020.00858 (PMC7290015; doi:10.3389/fpsyg.2020.00858)
Supplement: Supplementary file 3 [file Table_3.PDF]

Detailed Results of MANOVAs, ANOVAs, and Follow-up Paired t-tests. Note: In all Paired Samples Tests d' was hand calculated only for results for which $p < .05$ with the numerator as the given difference score and the denominator either as the SD of the non-VR condition for comparisons involving the VR format and the IMG condition for comparisons between 2D and IMG conditions.

Study 1 MANOVA of Satisfaction Ratings

Multivariate Tests[a]

Effect			Value	F	Hypothesis df	Error df	Sig.	Partial Eta Squared	Noncent. Parameter	Observed Power[d]
Between Subjects	Intercept	Pillai's Trace	.989	207.624[b]	10.000	24.000	.000	.989	2076.235	1.000
		Wilks' Lambda	.011	207.624[b]	10.000	24.000	.000	.989	2076.235	1.000
		Hotelling's Trace	86.510	207.624[b]	10.000	24.000	.000	.989	2076.235	1.000
		Roy's Largest Root	86.510	207.624[b]	10.000	24.000	.000	.989	2076.235	1.000
	Order	Pillai's Trace	.561	.976	20.000	50.000	.505	.281	19.514	.605
		Wilks' Lambda	.515	.945[b]	20.000	48.000	.539	.282	18.898	.581
		Hotelling's Trace	.794	.913	20.000	46.000	.574	.284	18.269	.556
		Roy's Largest Root	.495	1.238[c]	10.000	25.000	.316	.331	12.378	.486
Within Subjects	Format	Pillai's Trace	.905	6.666[b]	20.000	14.000	.000	.905	133.313	.999
		Wilks' Lambda	.095	6.666[b]	20.000	14.000	.000	.905	133.313	.999
		Hotelling's Trace	9.522	6.666[b]	20.000	14.000	.000	.905	133.313	.999
		Roy's Largest Root	9.522	6.666[b]	20.000	14.000	.000	.905	133.313	.999
	Format * Order	Pillai's Trace	1.288	1.356	40.000	30.000	.195	.644	54.232	.818
		Wilks' Lambda	.073	1.891[b]	40.000	28.000	.040	.730	75.631	.938
		Hotelling's Trace	7.758	2.521	40.000	26.000	.008	.795	100.849	.984

	Roy's Largest Root	7.058	5.293[c]	20.000	15.000	.001	.876	105.863	.997

Univariate Tests

Source	Measure		Type III Sum of Squares	df	Mean Square	F	Sig.	Partial Eta Squared	Noncent. Parameter	Observed Power[a]
Format	credibleasawaytoimproveselfregulationandenhancewellbeing	Sphericity Assumed	13.574	2	6.787	11.183	.000	.253	22.366	.990
		Greenhouse-Geisser	13.574	1.972	6.883	11.183	.000	.253	22.054	.989
		Huynh-Feldt	13.574	2.000	6.787	11.183	.000	.253	22.366	.990
		Lower-bound	13.574	1.000	13.574	11.183	.002	.253	11.183	.901
	credibleasaninterventionformentalhealthproblems	Sphericity Assumed	25.796	2	12.898	12.417	.000	.273	24.835	.995
		Greenhouse-Geisser	25.796	1.929	13.371	12.417	.000	.273	23.957	.994
		Huynh-Feldt	25.796	2.000	12.898	12.417	.000	.273	24.835	.995
		Lower-bound	25.796	1.000	25.796	12.417	.001	.273	12.417	.928
	easytocomplete	Sphericity Assumed	16.056	2	8.028	8.403	.001	.203	16.805	.957
		Greenhouse-Geisser	16.056	1.943	8.263	8.403	.001	.203	16.328	.953
		Huynh-Feldt	16.056	2.000	8.028	8.403	.001	.203	16.805	.957

		Lower-bound	16.056	1.000	16.056	8.403	.007	.203	8.403	.803
	helpful	Sphericity Assumed	18.667	2	9.333	10.034	.000	.233	20.069	.981
		Greenhouse-Geisser	18.667	1.821	10.251	10.034	.000	.233	18.272	.973
		Huynh-Feldt	18.667	2.000	9.333	10.034	.000	.233	20.069	.981
		Lower-bound	18.667	1.000	18.667	10.034	.003	.233	10.034	.867
	informative	Sphericity Assumed	26.056	2	13.028	17.254	.000	.343	34.508	1.000
		Greenhouse-Geisser	26.056	1.782	14.621	17.254	.000	.343	30.747	.999
		Huynh-Feldt	26.056	1.991	13.087	17.254	.000	.343	34.352	1.000
		Lower-bound	26.056	1.000	26.056	17.254	.000	.343	17.254	.981
	calming	Sphericity Assumed	18.667	2	9.333	8.419	.001	.203	16.838	.958
		Greenhouse-Geisser	18.667	1.924	9.701	8.419	.001	.203	16.199	.952
		Huynh-Feldt	18.667	2.000	9.333	8.419	.001	.203	16.838	.958
		Lower-bound	18.667	1.000	18.667	8.419	.007	.203	8.419	.804
	enjoyable	Sphericity Assumed	51.167	2	25.583	29.001	.000	.468	58.002	1.000
		Greenhouse-Geisser	51.167	1.924	26.599	29.001	.000	.468	55.786	1.000
		Huynh-Feldt	51.167	2.000	25.583	29.001	.000	.468	58.002	1.000
		Lower-bound	51.167	1.000	51.167	29.001	.000	.468	29.001	.999
	Iwouldrecommendthisexercisetoafriend	Sphericity Assumed	41.407	2	20.704	18.776	.000	.363	37.551	1.000

		Greenhouse-Geisser	41.407	1.913	21.641	18.776	.000	.363	35.925	1.000
		Huynh-Feldt	41.407	2.000	20.704	18.776	.000	.363	37.551	1.000
		Lower-bound	41.407	1.000	41.407	18.776	.000	.363	18.776	.988
	Iwouldcompletethisexerciseagain	Sphericity Assumed	38.019	2	19.009	16.016	.000	.327	32.033	.999
		Greenhouse-Geisser	38.019	1.755	21.660	16.016	.000	.327	28.113	.998
		Huynh-Feldt	38.019	1.958	19.413	16.016	.000	.327	31.366	.999
		Lower-bound	38.019	1.000	38.019	16.016	.000	.327	16.016	.973
	distressing	Sphericity Assumed	1.056	2	.528	.903	.410	.027	1.807	.200
		Greenhouse-Geisser	1.056	1.935	.545	.903	.407	.027	1.749	.197
		Huynh-Feldt	1.056	2.000	.528	.903	.410	.027	1.807	.200
		Lower-bound	1.056	1.000	1.056	.903	.349	.027	.903	.152

Paired Samples Test

		Paired Differences								
					95% Confidence Interval of the Difference					
		Mean	Std. Deviation	Std. Error Mean	Lower	Upper	t	df	Sig. (2-tailed)	d'
Pair 1	VR_credible as a way to improve self-regulation and enhance well-being - 2D_credible as a way to improve self-regulation and enhance well-being	.861	1.099	.183	.489	1.233	4.699	35	.000	.86
Pair 2	VR_credible as an intervention for mental health problems associated with stressful/traumatic life events like anxiety, depression, PTSD, and dissociation - 2D_credible as an intervention for mental health problems associated with stressful/traumatic life events like anxiety, depression, PTSD, and dissociation	1.194	1.489	.248	.691	1.698	4.812	35	.000	.93

Pair 3	VR_easy to complete - 2D_easy to complete	.472	1.424	.237	-.010	.954	1.990	35	.054	ns
Pair 4	VR_helpful – 2D_helpful	1.000	1.242	.207	.580	1.420	4.830	35	.000	.81
Pair 5	VR_informative – 2D_informative	1.056	1.264	.211	.628	1.483	5.012	35	.000	.87
Pair 6	VR_calming – 2D_calming	1.000	1.586	.264	.463	1.537	3.784	35	.001	.79
Pair 7	VR_enjoyable – 2D_enjoyable	1.417	1.273	.212	.986	1.848	6.675	35	.000	1.16
Pair 8	VR_I would recommend this exercise to a friend - 2D_I would recommend this exercise to a friend	1.500	1.558	.260	.973	2.027	5.775	35	.000	1.06
Pair 9	VR_I would complete this exercise again - 2D_I would complete this exercise again	1.444	1.629	.272	.893	1.996	5.320	35	.000	.93
Pair 10	VR_distressing (e.g., made me anxious or upset) - 2D_distressing (e.g., made me anxious or upset)	-.194	.980	.163	-.526	.137	-1.190	35	.242	ns

Paired Samples Test

		Paired Differences					t	df	Sig. (2-tailed)	d'
		Mean	Std. Deviation	Std. Error Mean	95% Confidence Interval of the Difference					
					Lower	Upper				
Pair 1	VR_credible as a way to improve self-regulation and enhance well-being - IMG_credible as a way to improve self-regulation and enhance well-being	.528	1.028	.171	.180	.876	3.081	35	.004	.46
Pair 2	VR_credible as an intervention for mental health problems associated with stressful/traumatic life events like anxiety, depression, PTSD, and dissociation - IMG_credible as an intervention for mental health problems associated with stressful/traumatic life events like anxiety, depression, PTSD, and dissociation	.667	1.265	.211	.239	1.095	3.162	35	.003	.64

Pair 3	VR_easy to complete - IMG_easy to complete	-.472	1.320	.220	-.919	-.026	-2.147	35	.039	.49
Pair 4	VR_helpful – IMG_helpful	.667	1.219	.203	.254	1.079	3.282	35	.002	.69
Pair 5	VR_informative – IMG_informative	1.028	1.383	.231	.560	1.496	4.458	35	.000	.76
Pair 6	VR_calming – IMG_calming	.667	1.454	.242	.175	1.159	2.751	35	.009	.62
Pair 7	VR_enjoyable – IMG_enjoyable	1.500	1.276	.213	1.068	1.932	7.052	35	.000	1.21
Pair 8	VR_I would recommend this exercise to a friend - IMG_I would recommend this exercise to a friend	.944	1.330	.222	.495	1.394	4.261	35	.000	.82
Pair 9	VR_I would complete this exercise again - IMG_I would complete this exercise again	.861	1.268	.211	.432	1.290	4.074	35	.000	.72
Pair 10	VR_distressing (e.g., made me anxious or upset) - IMG_distressing (e.g., made me anxious or upset)	.028	1.158	.193	-.364	.420	.144	35	.886	ns

Paired Samples Test

		Paired Differences								
					95% Confidence Interval of the Difference					
		Mean	Std. Deviation	Std. Error Mean	Lower	Upper	t	df	Sig. (2-tailed)	d'
Pair 1	2D_credible as a way to improve self-regulation and enhance well-being - IMG_credible as a way to improve self-regulation and enhance well-being	-.333	1.121	.187	-.713	.046	-1.784	35	.083	ns
Pair 2	2D_credible as an intervention for mental health problems associated with stressful/traumatic life events like anxiety, depression, PTSD, and dissociation - IMG_credible as an intervention for mental health problems associated with stressful/traumatic life events like anxiety, depression, PTSD, and dissociation	-.528	1.464	.244	-1.023	-.033	-2.164	35	.037	.51

Pair 3	2D_easy to complete - IMG_easy to complete	-.944	1.530	.255	-1.462	-.427	-3.705	35	.001	.98
Pair 4	2D_helpful – IMG_helpful	-.333	1.549	.258	-.858	.191	-1.291	35	.205	ns
Pair 5	2D_informative – IMG_informative	-.028	1.183	.197	-.428	.372	-.141	35	.889	ns
Pair 6	2D_Calming – IMG_calming	-.333	1.493	.249	-.838	.172	-1.340	35	.189	ns
Pair 7	2D_enjoyable – IMG_Enjoyable	.083	1.556	.259	-.443	.610	.321	35	.750	ns
Pair 8	2D_I would recommend this exercise to a friend - IMG_I would recommend this exercise to a friend	-.556	1.501	.250	-1.064	-.048	-2.220	35	.033	.48
Pair 9	2D_I would complete this exercise again - IMG_I would complete this exercise again	-.583	1.663	.277	-1.146	-.021	-2.105	35	.043	.49
Pair 10	2D_distressing (e.g., made me anxious or upset) - IMG_distressing (e.g., made me anxious or upset)	.222	1.098	.183	-.149	.594	1.214	35	.233	ns

Study 1 MANOVA of Positive Affect Ratings (mDES Positive)

Multivariate Tests[a]

Effect			Value	F	Hypothesis df	Error df	Sig.	Partial Eta Squared	Noncent. Parameter	Observed Power[d]
Between Subjects	Intercept	Pillai's Trace	.950	45.149[b]	10.000	24.000	.000	.950	451.487	1.000
		Wilks' Lambda	.050	45.149[b]	10.000	24.000	.000	.950	451.487	1.000
		Hotelling's Trace	18.812	45.149[b]	10.000	24.000	.000	.950	451.487	1.000
		Roy's Largest Root	18.812	45.149[b]	10.000	24.000	.000	.950	451.487	1.000
	Order	Pillai's Trace	.445	.715	20.000	50.000	.793	.222	14.290	.441
		Wilks' Lambda	.599	.701[b]	20.000	48.000	.805	.226	14.016	.427
		Hotelling's Trace	.596	.686	20.000	46.000	.819	.230	13.719	.412
		Roy's Largest Root	.425	1.063[c]	10.000	25.000	.424	.298	10.632	.418
Within Subjects	Format	Pillai's Trace	.953	14.279[b]	20.000	14.000	.000	.953	285.582	1.000
		Wilks' Lambda	.047	14.279[b]	20.000	14.000	.000	.953	285.582	1.000
		Hotelling's Trace	20.399	14.279[b]	20.000	14.000	.000	.953	285.582	1.000
		Roy's Largest Root	20.399	14.279[b]	20.000	14.000	.000	.953	285.582	1.000
	Format * Order	Pillai's Trace	1.336	1.511	40.000	30.000	.122	.668	60.423	.869
		Wilks' Lambda	.102	1.487[b]	40.000	28.000	.137	.680	59.492	.847
		Hotelling's Trace	4.479	1.456	40.000	26.000	.158	.691	58.225	.817
		Roy's Largest Root	3.094	2.320[c]	20.000	15.000	.051	.756	46.407	.816

Univariate Tests

Source	Measure		Type III Sum of Squares	df	Mean Square	F	Sig.	Partial Eta Squared	Noncent. Parameter	Observed Power[a]
Format	Amusedfunlovingsilly	Sphericity Assumed	263.574	2	131.787	39.950	.000	.548	79.899	1.000
		Greenhouse-Geisser	263.574	1.763	149.541	39.950	.000	.548	70.414	1.000
		Huynh-Feldt	263.574	1.967	133.981	39.950	.000	.548	78.591	1.000
		Lower-bound	263.574	1.000	263.574	39.950	.000	.548	39.950	1.000
	Awewonderamazement	Sphericity Assumed	597.852	2	298.926	79.929	.000	.708	159.858	1.000
		Greenhouse-Geisser	597.852	1.918	311.701	79.929	.000	.708	153.306	1.000
		Huynh-Feldt	597.852	2.000	298.926	79.929	.000	.708	159.858	1.000
		Lower-bound	597.852	1.000	597.852	79.929	.000	.708	79.929	1.000
	Gratefulappreciativethankful	Sphericity Assumed	71.685	2	35.843	10.442	.000	.240	20.883	.985
		Greenhouse-Geisser	71.685	1.683	42.594	10.442	.000	.240	17.573	.970
		Huynh-Feldt	71.685	1.871	38.318	10.442	.000	.240	19.534	.980
		Lower-bound	71.685	1.000	71.685	10.442	.003	.240	10.442	.880
	Hopefuloptimisticencouraged	Sphericity Assumed	83.352	2	41.676	13.356	.000	.288	26.712	.997
		Greenhouse-Geisser	83.352	1.963	42.454	13.356	.000	.288	26.222	.997
		Huynh-Feldt	83.352	2.000	41.676	13.356	.000	.288	26.712	.997
		Lower-bound	83.352	1.000	83.352	13.356	.001	.288	13.356	.944

	Inspiredupliftedelevated	Sphericity Assumed	143.463	2	71.731	21.204	.000	.391	42.407	1.000
		Greenhouse-Geisser	143.463	1.985	72.271	21.204	.000	.391	42.090	1.000
		Huynh-Feldt	143.463	2.000	71.731	21.204	.000	.391	42.407	1.000
		Lower-bound	143.463	1.000	143.463	21.204	.000	.391	21.204	.994
	Interestedalertcurious	Sphericity Assumed	325.241	2	162.620	40.965	.000	.554	81.931	1.000
		Greenhouse-Geisser	325.241	1.960	165.934	40.965	.000	.554	80.295	1.000
		Huynh-Feldt	325.241	2.000	162.620	40.965	.000	.554	81.931	1.000
		Lower-bound	325.241	1.000	325.241	40.965	.000	.554	40.965	1.000
	Joyfulgladhappy	Sphericity Assumed	72.463	2	36.231	12.973	.000	.282	25.945	.996
		Greenhouse-Geisser	72.463	1.999	36.243	12.973	.000	.282	25.937	.996
		Huynh-Feldt	72.463	2.000	36.231	12.973	.000	.282	25.945	.996
		Lower-bound	72.463	1.000	72.463	12.973	.001	.282	12.973	.938
	Loveclosenesstrust	Sphericity Assumed	10.296	2	5.148	1.408	.252	.041	2.816	.292
		Greenhouse-Geisser	10.296	1.849	5.568	1.408	.252	.041	2.603	.280
		Huynh-Feldt	10.296	2.000	5.148	1.408	.252	.041	2.816	.292
		Lower-bound	10.296	1.000	10.296	1.408	.244	.041	1.408	.211
	Proudconfidentselfassured	Sphericity Assumed	27.556	2	13.778	5.093	.009	.134	10.185	.804
		Greenhouse-Geisser	27.556	1.834	15.025	5.093	.011	.134	9.340	.778

		Huynh-Feldt	27.556	2.000	13.778	5.093	.009	.134	10.185	.804
		Lower-bound	27.556	1.000	27.556	5.093	.031	.134	5.093	.591
	Serenecontentpeaceful	Sphericity Assumed	64.389	2	32.194	8.465	.001	.204	16.931	.959
		Greenhouse-Geisser	64.389	1.879	34.270	8.465	.001	.204	15.906	.950
		Huynh-Feldt	64.389	2.000	32.194	8.465	.001	.204	16.931	.959
		Lower-bound	64.389	1.000	64.389	8.465	.006	.204	8.465	.806

Paired Samples Test

		Paired Differences								
					95% Confidence Interval of the Difference					
		Mean	Std. Deviation	Std. Error Mean	Lower	Upper	t	df	Sig. (2-tailed)	d'
Pair 1	VR_Amused, fun-loving, silly - 2D_Amused, fun-loving, silly	2.417	2.579	.430	1.544	3.289	5.623	35	.000	.95
Pair 2	VR_Awe, wonder, amazement - 2D_Awe, wonder, amazement	4.722	2.753	.459	3.791	5.654	10.293	35	.000	1.81
Pair 3	VR_Grateful, appreciative, thankful - 2D_Grateful, appreciative, thankful	1.806	2.539	.423	.946	2.665	4.267	35	.000	.63
Pair 4	VR_Hopeful, optimistic, encouraged - 2D_Hopeful, optimistic, encouraged	1.917	2.347	.391	1.123	2.711	4.900	35	.000	.35
Pair 5	VR_Inspired, uplifted, elevated - 2D_Inspired, uplifted, elevated	2.472	2.635	.439	1.581	3.364	5.630	35	.000	.86
Pair 6	VR_Interested, alert, curious - 2D_Interested, alert, curious	3.639	2.717	.453	2.720	4.558	8.037	35	.000	1.46
Pair 7	VR_Joyful, glad, happy - 2D_Joyful, glad, happy	1.694	2.315	.386	.911	2.478	4.391	35	.000	.57

Pair 8	VR_Love, closeness, trust - 2D_Love, closeness, trust	.556	3.093	.516	-.491	1.602	1.078	35	.289	ns
Pair 9	VR_Proud, confident, self-assured - 2D_Proud, confident, self-assured	1.222	2.508	.418	.374	2.071	2.924	35	.006	.44
Pair 10	VR_Serene, content, peaceful - 2D_Serene, content, peaceful	1.861	2.758	.460	.928	2.794	4.048	35	.000	.62

Paired Samples Test

		Paired Differences					t	df	Sig. (2-tailed)	d'
		Mean	Std. Deviation	Std. Error Mean	95% Confidence Interval of the Difference					
					Lower	Upper				
Pair 1	VR_Amused, fun-loving, silly - IMG_Amused, fun-loving, silly	3.778	2.331	.389	2.989	4.567	9.723	35	.000	1.47
Pair 2	VR_Awe, wonder, amazement - IMG_Awe, wonder, amazement	5.222	3.053	.509	4.189	6.255	10.263	35	.000	1.93
Pair 3	VR_Grateful, appreciative, thankful - IMG_Grateful, appreciative, thankful	1.639	2.440	.407	.813	2.464	4.031	35	.000	.51
Pair 4	VR_Hopeful, optimistic, encouraged - IMG_Hopeful, optimistic, encouraged	1.806	2.617	.436	.920	2.691	4.140	35	.000	.62
Pair 5	VR_Inspired, uplifted, elevated - IMG_Inspired, uplifted, elevated	2.417	2.557	.426	1.552	3.282	5.672	35	.000	.80
Pair 6	VR_Interested, alert, curious - IMG_Interested, alert, curious	3.722	2.690	.448	2.812	4.632	8.303	35	.000	1.36
Pair 7	VR_Joyful, glad, happy - IMG_Joyful, glad, happy	1.778	2.427	.405	.956	2.599	4.394	35	.000	.59

Pair 8	VR_Love, closeness, trust - IMG_Love, closeness, trust	.722	2.386	.398	-.085	1.529	1.816	35	.078	ns
Pair 9	VR_Proud, confident, self-assured - IMG_Proud, confident, self-assured	.778	1.958	.326	.115	1.440	2.383	35	.023	.24
Pair 10	VR_Serene, content, peaceful - IMG_Serene, content, peaceful	1.222	2.684	.447	.314	2.131	2.732	35	.010	.40

Paired Samples Test

		Paired Differences								
					95% Confidence Interval of the Difference					
		Mean	Std. Deviation	Std. Error Mean	Lower	Upper	t	df	Sig. (2-tailed)	d'
Pair 1	2D_Amused, fun-loving, silly - IMG_Amused, fun-loving, silly	1.361	2.919	.487	.373	2.349	2.797	35	.008	.53
Pair 2	2D_Awe, wonder, amazement - IMG_Awe, wonder, amazement	.500	2.490	.415	-.342	1.342	1.205	35	.236	ns
Pair 3	2D_Grateful, appreciative, thankful - IMG_Grateful, appreciative, thankful	-.167	3.342	.557	-1.298	.964	-.299	35	.767	ns
Pair 4	2D_Hopeful, optimistic, encouraged - IMG_Hopeful, optimistic, encouraged	-.111	2.670	.445	-1.015	.792	-.250	35	.804	ns
Pair 5	2D_Inspired, uplifted, elevated - IMG_Inspired, uplifted, elevated	-.056	2.651	.442	-.952	.841	-.126	35	.901	ns
Pair 6	2D_Interested, alert, curious - IMG_Interested, alert, curious	.083	3.138	.523	-.979	1.145	.159	35	.874	ns
Pair 7	2D_Joyful, glad, happy - IMG_Joyful, glad, happy	.083	2.419	.403	-.735	.902	.207	35	.837	ns

Pair 8	2D_Love, closeness, trust - IMG_Love, closeness, trust	.167	2.613	.436	-.717	1.051	.383	35	.704	ns
Pair 9	2D_Proud, confident, self-assured - IMG_Proud, confident, self-assured	-.444	2.524	.421	-1.298	.409	-1.057	35	.298	ns
Pair 10	2D_Serene, content, peaceful - IMG_Serene, content, peaceful	-.639	3.118	.520	-1.694	.416	-1.229	35	.227	ns

Study 1 MANOVA of Negative Affect Ratings (mDES Negative)

Multivariate Tests[a]

Effect			Value	F	Hypothesis df	Error df	Sig.	Partial Eta Squared	Noncent. Parameter	Observed Power[d]
Between Subjects	Intercept	Pillai's Trace	.529	2.697[b]	10.000	24.000	.023	.529	26.967	.871
		Wilks' Lambda	.471	2.697[b]	10.000	24.000	.023	.529	26.967	.871
		Hotelling's Trace	1.124	2.697[b]	10.000	24.000	.023	.529	26.967	.871
		Roy's Largest Root	1.124	2.697[b]	10.000	24.000	.023	.529	26.967	.871
	Order	Pillai's Trace	.614	1.107	20.000	50.000	.373	.307	22.131	.677
		Wilks' Lambda	.472	1.095[b]	20.000	48.000	.385	.313	21.900	.665
		Hotelling's Trace	.940	1.081	20.000	46.000	.399	.320	21.621	.651
		Roy's Largest Root	.671	1.677[c]	10.000	25.000	.142	.401	16.769	.643
Within Subjects	Format	Pillai's Trace	.595	2.097[b]	14.000	20.000	.064	.595	29.351	.787
		Wilks' Lambda	.405	2.097[b]	14.000	20.000	.064	.595	29.351	.787
		Hotelling's Trace	1.468	2.097[b]	14.000	20.000	.064	.595	29.351	.787
		Roy's Largest Root	1.468	2.097[b]	14.000	20.000	.064	.595	29.351	.787
	Format * Order	Pillai's Trace	.916	1.268	28.000	42.000	.238	.458	35.515	.798
		Wilks' Lambda	.276	1.290[b]	28.000	40.000	.227	.474	36.113	.798
		Hotelling's Trace	1.924	1.305	28.000	38.000	.220	.490	36.549	.795
		Roy's Largest Root	1.439	2.159[c]	14.000	21.000	.054	.590	30.226	.812

Study 2 MANOVA of Satisfaction Ratings

Multivariate Tests[a]

Effect			Value	F	Hypothesis df	Error df	Sig.	Partial Eta Squared	Noncent. Parameter	Observed Power[c]
Between Subjects	Intercept	Pillai's Trace	.984	222.857[b]	10.000	37.000	.000	.984	2228.574	1.000
		Wilks' Lambda	.016	222.857[b]	10.000	37.000	.000	.984	2228.574	1.000
		Hotelling's Trace	60.232	222.857[b]	10.000	37.000	.000	.984	2228.574	1.000
		Roy's Largest Root	60.232	222.857[b]	10.000	37.000	.000	.984	2228.574	1.000
	Order	Pillai's Trace	.262	1.310[b]	10.000	37.000	.261	.262	13.102	.564
		Wilks' Lambda	.738	1.310[b]	10.000	37.000	.261	.262	13.102	.564
		Hotelling's Trace	.354	1.310[b]	10.000	37.000	.261	.262	13.102	.564
		Roy's Largest Root	.354	1.310[b]	10.000	37.000	.261	.262	13.102	.564
Within Subjects	Format	Pillai's Trace	.902	12.439[b]	20.000	27.000	.000	.902	248.789	1.000
		Wilks' Lambda	.098	12.439[b]	20.000	27.000	.000	.902	248.789	1.000
		Hotelling's Trace	9.214	12.439[b]	20.000	27.000	.000	.902	248.789	1.000
		Roy's Largest Root	9.214	12.439[b]	20.000	27.000	.000	.902	248.789	1.000
	Format * Order	Pillai's Trace	.582	1.883[b]	20.000	27.000	.063	.582	37.665	.854
		Wilks' Lambda	.418	1.883[b]	20.000	27.000	.063	.582	37.665	.854
		Hotelling's Trace	1.395	1.883[b]	20.000	27.000	.063	.582	37.665	.854
		Roy's Largest Root	1.395	1.883[b]	20.000	27.000	.063	.582	37.665	.854

Univariate Tests

Source	Measure		Type III Sum of Squares	df	Mean Square	F	Sig.	Partial Eta Squared	Noncent. Parameter	Observed Power[a]
Format	credibleasawaytoimproveselfregulationandenhancewellbeing	Sphericity Assumed	43.556	2	21.778	29.530	.000	.391	59.061	1.000
		Greenhouse-Geisser	43.556	1.694	25.708	29.530	.000	.391	50.031	1.000
		Huynh-Feldt	43.556	1.790	24.328	29.530	.000	.391	52.869	1.000
		Lower-bound	43.556	1.000	43.556	29.530	.000	.391	29.530	1.000
	credibleasaninterventionformentalhealthproblems	Sphericity Assumed	55.816	2	27.908	34.631	.000	.430	69.263	1.000
		Greenhouse-Geisser	55.816	1.706	32.725	34.631	.000	.430	59.068	1.000
		Huynh-Feldt	55.816	1.803	30.955	34.631	.000	.430	62.445	1.000
		Lower-bound	55.816	1.000	55.816	34.631	.000	.430	34.631	1.000
	easytocomplete	Sphericity Assumed	1.483	2	.741	1.533	.221	.032	3.066	.318
		Greenhouse-Geisser	1.483	1.548	.958	1.533	.225	.032	2.373	.279
		Huynh-Feldt	1.483	1.626	.912	1.533	.224	.032	2.493	.286
		Lower-bound	1.483	1.000	1.483	1.533	.222	.032	1.533	.228
	helpful	Sphericity Assumed	25.566	2	12.783	21.883	.000	.322	43.765	1.000
		Greenhouse-Geisser	25.566	1.746	14.644	21.883	.000	.322	38.204	1.000
		Huynh-Feldt	25.566	1.848	13.831	21.883	.000	.322	40.449	1.000
		Lower-bound	25.566	1.000	25.566	21.883	.000	.322	21.883	.996

	informative	Sphericity Assumed	47.722	2	23.861	33.338	.000	.420	66.676	1.000
		Greenhouse-Geisser	47.722	1.694	28.172	33.338	.000	.420	56.474	1.000
		Huynh-Feldt	47.722	1.790	26.660	33.338	.000	.420	59.677	1.000
		Lower-bound	47.722	1.000	47.722	33.338	.000	.420	33.338	1.000
	calming	Sphericity Assumed	23.983	2	11.991	18.643	.000	.288	37.287	1.000
		Greenhouse-Geisser	23.983	1.787	13.423	18.643	.000	.288	33.309	1.000
		Huynh-Feldt	23.983	1.894	12.660	18.643	.000	.288	35.318	1.000
		Lower-bound	23.983	1.000	23.983	18.643	.000	.288	18.643	.988
	enjoyable	Sphericity Assumed	96.347	2	48.174	70.009	.000	.603	140.018	1.000
		Greenhouse-Geisser	96.347	1.979	48.691	70.009	.000	.603	138.530	1.000
		Huynh-Feldt	96.347	2.000	48.174	70.009	.000	.603	140.018	1.000
		Lower-bound	96.347	1.000	96.347	70.009	.000	.603	70.009	1.000
	Iwouldrecommendthisexercisetoafriend	Sphericity Assumed	71.733	2	35.866	40.442	.000	.468	80.885	1.000
		Greenhouse-Geisser	71.733	2.000	35.873	40.442	.000	.468	80.869	1.000
		Huynh-Feldt	71.733	2.000	35.866	40.442	.000	.468	80.885	1.000
		Lower-bound	71.733	1.000	71.733	40.442	.000	.468	40.442	1.000
	Iwouldcompletethisexerciseagain	Sphericity Assumed	80.792	2	40.396	43.110	.000	.484	86.219	1.000
		Greenhouse-Geisser	80.792	1.796	44.980	43.110	.000	.484	77.433	1.000

		Huynh-Feldt	80.792	1.905	42.406	43.110	.000	.484	82.132	1.000
		Lower-bound	80.792	1.000	80.792	43.110	.000	.484	43.110	1.000
	distressing	Sphericity Assumed	.899	2	.450	1.821	.168	.038	3.641	.371
		Greenhouse-Geisser	.899	1.898	.474	1.821	.170	.038	3.455	.361
		Huynh-Feldt	.899	2.000	.450	1.821	.168	.038	3.641	.371
		Lower-bound	.899	1.000	.899	1.821	.184	.038	1.821	.262

Paired Samples Test

		Paired Differences					t	df	Sig. (2-tailed)	d'
		Mean	Std. Deviation	Std. Error Mean	95% Confidence Interval of the Difference Lower	95% Confidence Interval of the Difference Upper				
Pair 1	credibleasawaytoimproveselfregulationandenhancewellbeing_VR - credibleasawaytoimproveselfregulationandenhancewellbeing_2D	1.33333	.93019	.13426	1.06324	1.60343	9.931	47	.000	1.30
Pair 2	credibleasaninterventionformentalhealthproblems_VR - credibleasaninterventionformentalhealthproblems_2D	1.51042	.98656	.14240	1.22395	1.79688	10.607	47	.000	1.59
Pair 3	easytocomplete_VR - easytocomplete_2D	-.17708	.76136	.10989	-.39816	.04399	-1.611	47	.114	ns
Pair 4	helpful_VR - helpful_2D	1.01042	.84103	.12139	.76621	1.25463	8.324	47	.000	.82
Pair 5	informative_VR - informative_2D	.95833	.93873	.13549	.68576	1.23091	7.073	47	.000	.72
Pair 6	calming_VR - calming_2D	.96875	1.05893	.15284	.66127	1.27623	6.338	47	.000	.80
Pair 7	enjoyable_VR - enjoyable_2D	1.81250	1.21439	.17528	1.45988	2.16512	10.341	47	.000	1.58
Pair 8	Iwouldrecommendthisexercisetoafriend_VR - Iwouldrecommendthisexercisetoafriend_2D	1.64583	1.32070	.19063	1.26234	2.02932	8.634	47	.000	1.37
Pair 9	Iwouldcompletethisexerciseagain_VR - Iwouldcompletethisexerciseagain_2D	1.72917	1.36428	.19692	1.33302	2.12531	8.781	47	.000	1.24
Pair 10	distressing_VR - distressing_2D	-.02083	.73628	.10627	-.23463	.19296	-.196	47	.845	ns

Paired Samples Test

		Paired Differences					t	df	Sig. (2-tailed)	d'
		Mean	Std. Deviation	Std. Error Mean	95% Confidence Interval of the Difference Lower	95% Confidence Interval of the Difference Upper				
Pair 1	credibleasawaytoimproveselfregulationandenhancewellbeing_VR - credibleasawaytoimproveselfregulationandenhancewellbeing_IMG	.83333	1.28962	.18614	.45887	1.20780	4.477	47	.000	.67
Pair 2	credibleasaninterventionformentalhealthproblems_VR - credibleasaninterventionformentalhealthproblems_IMG	.93750	1.43892	.20769	.51968	1.35532	4.514	47	.000	.76
Pair 3	easytocomplete_VR - easytocomplete_IMG	-.23958	1.19836	.17297	-.58755	.10838	-1.385	47	.173	ns
Pair 4	helpful_VR - helpful_IMG	.68750	1.18333	.17080	.34390	1.03110	4.025	47	.000	.53
Pair 5	informative_VR - informative_IMG	1.37500	1.35073	.19496	.98279	1.76721	7.053	47	.000	.96
Pair 6	calming_VR - calming_IMG	.69792	1.32384	.19108	.31351	1.08232	3.652	47	.001	.56
Pair 7	enjoyable_VR - enjoyable_IMG	1.64583	1.22890	.17738	1.28900	2.00267	9.279	47	.000	1.30
Pair 8	Iwouldrecommendthisexercisetoafriend_VR - Iwouldrecommendthisexercisetoafriend_IMG	1.28125	1.32852	.19176	.89549	1.66701	6.682	47	.000	1.03
Pair 9	Iwouldcompletethisexerciseagain_VR - Iwouldcompletethisexerciseagain_IMG	1.39583	1.55042	.22378	.94564	1.84603	6.237	47	.000	1.05
Pair 10	distressing_VR - distressing_IMG	-.17708	.74011	.10683	-.39199	.03782	-1.658	47	.104	ns

Paired Samples Test

		Paired Differences					t	df	Sig. (2-tailed)	d'
					95% Confidence Interval of the Difference					
		Mean	Std. Deviation	Std. Error Mean	Lower	Upper				
Pair 1	credibleasawaytoimproveselfregulationandenhancewellbeing_2D - credibleasawaytoimproveselfregulationandenhancewellbeing_IMG	-.50000	1.37222	.19806	-.89845	-.10155	-2.524	47	.015	.40
Pair 2	credibleasaninterventionformentalhealthproblems_2D - credibleasaninterventionformentalhealthproblems_IMG	-.57292	1.41793	.20466	-.98464	-.16119	-2.799	47	.007	.47
Pair 3	easytocomplete_2D - easytocomplete_IMG	-.06250	.93185	.13450	-.33308	.20808	-.465	47	.644	ns
Pair 4	helpful_2D - helpful_IMG	-.32292	1.18272	.17071	-.66634	.02051	-1.892	47	.065	Ns
Pair 5	informative_2D - informative_IMG	.41667	1.29374	.18674	.04100	.79233	2.231	47	.030	.29
Pair 6	calming_2D - calming_IMG	-.27083	1.01561	.14659	-.56574	.02407	-1.848	47	.071	ns
Pair 7	enjoyable_2D - enjoyable_IMG	-.16667	1.11724	.16126	-.49108	.15775	-1.034	47	.307	ns
Pair 8	Iwouldrecommendthisexercisetoafriend_2D - Iwouldrecommendthisexercisetoafriend_IMG	-.36458	1.32785	.19166	-.75015	.02098	-1.902	47	.063	ns
Pair 9	Iwouldcompletethisexerciseagain_2D - Iwouldcompletethisexerciseagain_IMG	-.33333	1.17298	.16931	-.67393	.00727	-1.969	47	.055	ns
Pair 10	distressing_2D - distressing_IMG	-.15625	.61156	.08827	-.33383	.02133	-1.770	47	.083	ns

Study 2 MANOVA of Positive Affect Ratings (mDES Positive)

Multivariate Tests[a]

Effect			Value	F	Hypothesis df	Error df	Sig.	Partial Eta Squared	Noncent. Parameter	Observed Power[c]
Between Subjects	Intercept	Pillai's Trace	.905	35.419[b]	10.000	37.000	.000	.905	354.192	1.000
		Wilks' Lambda	.095	35.419[b]	10.000	37.000	.000	.905	354.192	1.000
		Hotelling's Trace	9.573	35.419[b]	10.000	37.000	.000	.905	354.192	1.000
		Roy's Largest Root	9.573	35.419[b]	10.000	37.000	.000	.905	354.192	1.000
	Order	Pillai's Trace	.192	.878[b]	10.000	37.000	.562	.192	8.776	.379
		Wilks' Lambda	.808	.878[b]	10.000	37.000	.562	.192	8.776	.379
		Hotelling's Trace	.237	.878[b]	10.000	37.000	.562	.192	8.776	.379
		Roy's Largest Root	.237	.878[b]	10.000	37.000	.562	.192	8.776	.379
Within Subjects	Format	Pillai's Trace	.838	6.995[b]	20.000	27.000	.000	.838	139.906	1.000
		Wilks' Lambda	.162	6.995[b]	20.000	27.000	.000	.838	139.906	1.000
		Hotelling's Trace	5.182	6.995[b]	20.000	27.000	.000	.838	139.906	1.000
		Roy's Largest Root	5.182	6.995[b]	20.000	27.000	.000	.838	139.906	1.000
	Format * Order	Pillai's Trace	.318	.630[b]	20.000	27.000	.855	.318	12.591	.315
		Wilks' Lambda	.682	.630[b]	20.000	27.000	.855	.318	12.591	.315
		Hotelling's Trace	.466	.630[b]	20.000	27.000	.855	.318	12.591	.315
		Roy's Largest Root	.466	.630[b]	20.000	27.000	.855	.318	12.591	.315

Univariate Tests

Source	Measure		Type III Sum of Squares	df	Mean Square	F	Sig.	Partial Eta Squared	Noncent. Parameter	Observed Power[a]
Format	Amusedfunlovingsilly	Sphericity Assumed	271.566	2	135.783	42.609	.000	.481	85.217	1.000
		Greenhouse-Geisser	271.566	1.765	153.899	42.609	.000	.481	75.186	1.000
		Huynh-Feldt	271.566	1.870	145.258	42.609	.000	.481	79.658	1.000
		Lower-bound	271.566	1.000	271.566	42.609	.000	.481	42.609	1.000
	Awewonderamazement	Sphericity Assumed	625.219	2	312.609	103.926	.000	.693	207.852	1.000
		Greenhouse-Geisser	625.219	1.909	327.472	103.926	.000	.693	198.418	1.000
		Huynh-Feldt	625.219	2.000	312.609	103.926	.000	.693	207.852	1.000
		Lower-bound	625.219	1.000	625.219	103.926	.000	.693	103.926	1.000
	Gratefulappreciativethankful	Sphericity Assumed	76.191	2	38.095	5.071	.008	.099	10.142	.808
		Greenhouse-Geisser	76.191	1.205	63.253	5.071	.022	.099	6.108	.652
		Huynh-Feldt	76.191	1.246	61.145	5.071	.021	.099	6.319	.662
		Lower-bound	76.191	1.000	76.191	5.071	.029	.099	5.071	.597
	Hopefuloptimisticencouraged	Sphericity Assumed	59.753	2	29.877	21.306	.000	.317	42.613	1.000
		Greenhouse-Geisser	59.753	1.864	32.052	21.306	.000	.317	39.721	1.000
		Huynh-Feldt	59.753	1.982	30.145	21.306	.000	.317	42.233	1.000
		Lower-bound	59.753	1.000	59.753	21.306	.000	.317	21.306	.995

	Inspiredupliftedelevated	Sphericity Assumed	162.667	2	81.333	38.315	.000	.454	76.631	1.000
		Greenhouse-Geisser	162.667	1.917	84.844	38.315	.000	.454	73.460	1.000
		Huynh-Feldt	162.667	2.000	81.333	38.315	.000	.454	76.631	1.000
		Lower-bound	162.667	1.000	162.667	38.315	.000	.454	38.315	1.000
	Interestedalertcurious	Sphericity Assumed	440.385	2	220.193	79.181	.000	.633	158.362	1.000
		Greenhouse-Geisser	440.385	1.850	238.040	79.181	.000	.633	146.489	1.000
		Huynh-Feldt	440.385	1.966	223.992	79.181	.000	.633	155.676	1.000
		Lower-bound	440.385	1.000	440.385	79.181	.000	.633	79.181	1.000
	Joyfulgladhappy	Sphericity Assumed	151.531	2	75.766	32.276	.000	.412	64.551	1.000
		Greenhouse-Geisser	151.531	1.809	83.771	32.276	.000	.412	58.383	1.000
		Huynh-Feldt	151.531	1.920	78.942	32.276	.000	.412	61.954	1.000
		Lower-bound	151.531	1.000	151.531	32.276	.000	.412	32.276	1.000
	Loveclosenesstrust	Sphericity Assumed	31.191	2	15.595	7.212	.001	.136	14.423	.927
		Greenhouse-Geisser	31.191	1.841	16.943	7.212	.002	.136	13.276	.911
		Huynh-Feldt	31.191	1.956	15.948	7.212	.001	.136	14.104	.923
		Lower-bound	31.191	1.000	31.191	7.212	.010	.136	7.212	.748
	Proudconfidentselfassured	Sphericity Assumed	22.823	2	11.411	8.560	.000	.157	17.120	.963
		Greenhouse-Geisser	22.823	1.802	12.668	8.560	.001	.157	15.421	.948

		Huynh-Feldt	22.823	1.911	11.941	8.560	.000	.157	16.360	.957
		Lower-bound	22.823	1.000	22.823	8.560	.005	.157	8.560	.817
	Serenecontentpeaceful	Sphericity Assumed	104.128	2	52.064	19.588	.000	.299	39.176	1.000
		Greenhouse-Geisser	104.128	1.932	53.897	19.588	.000	.299	37.844	1.000
		Huynh-Feldt	104.128	2.000	52.064	19.588	.000	.299	39.176	1.000
		Lower-bound	104.128	1.000	104.128	19.588	.000	.299	19.588	.991

Paired Samples Test

		Paired Differences								
		Mean	Std. Deviation	Std. Error Mean	95% Confidence Interval of the Difference Lower	Upper	t	df	Sig. (2-tailed)	d'
Pair 1	Amusedfunlovingsilly_VR - Amusedfunlovingsilly_2D	2.85417	2.31774	.33454	2.18117	3.52717	8.532	47	.000	1.20
Pair 2	Awewonderamazement_VR - Awewonderamazement_2D	3.93750	2.36694	.34164	3.25021	4.62479	11.525	47	.000	2.05
Pair 3	Gratefulappreciativethankful_VR - Gratefulappreciativethankful_2D	1.78125	1.95945	.28282	1.21229	2.35021	6.298	47	.000	.64
Pair 4	Hopefuloptimisticencouraged_VR - Hopefuloptimisticencouraged_2D	1.40625	1.75237	.25293	.89741	1.91509	5.560	47	.000	.56
Pair 5	Inspiredupliftedelevated_VR - Inspiredupliftedelevated_2D	2.33333	2.22733	.32149	1.68658	2.98008	7.258	47	.000	.96
Pair 6	Interestedalertcurious_VR - Interestedalertcurious_2D	3.22917	2.04720	.29549	2.63472	3.82361	10.928	47	.000	1.32
Pair 7	Joyfulgladhappy_VR - Joyfulgladhappy_2D	2.09375	2.09522	.30242	1.48536	2.70214	6.923	47	.000	.79
Pair 8	Loveclosenesstrust_VR - Loveclosenesstrust_2D	1.13542	1.85007	.26703	.59821	1.67262	4.252	47	.000	.47
Pair 9	Proudconfidentselfassured_VR - Proudconfidentselfassured_2D	.95833	1.79192	.25864	.43801	1.47865	3.705	47	.001	.35
Pair 10	Serenecontentpeaceful_VR - Serenecontentpeaceful_2D	1.95833	2.19969	.31750	1.31961	2.59706	6.168	47	.000	.70

Paired Samples Test

		Paired Differences					t	df	Sig. (2-tailed)	d'
		Mean	Std. Deviation	Std. Error Mean	95% Confidence Interval of the Difference					
					Lower	Upper				
Pair 1	Amusedfunlovingsilly_VR - Amusedfunlovingsilly_IMG	2.96875	2.93440	.42354	2.11669	3.82081	7.009	47	.000	1.10
Pair 2	Awewonderamazement_VR - Awewonderamazement_IMG	4.78125	2.69930	.38961	3.99745	5.56505	12.272	47	.000	2.12
Pair 3	Gratefulappreciativethankful_VR - Gratefulappreciativethankful_IMG	.85417	4.08156	.58912	-.33099	2.03933	1.450	47	.154	ns
Pair 4	Hopefuloptimisticencouraged_VR - Hopefuloptimisticencouraged_IMG	1.32292	2.03817	.29418	.73109	1.91474	4.497	47	.000	.50
Pair 5	Inspireduplifteddelevated_VR - Inspireduplifteddelevated_IMG	2.16667	2.38197	.34381	1.47502	2.85832	6.302	47	.000	.80
Pair 6	Interestedalertcurious_VR - Interestedalertcurious_IMG	4.05208	2.64020	.38108	3.28545	4.81872	10.633	47	.000	1.54
Pair 7	Joyfulgladhappy_VR - Joyfulgladhappy_IMG	2.25000	2.52435	.36436	1.51701	2.98299	6.175	47	.000	.77
Pair 8	Loveclosenesstrust_VR - Loveclosenesstrust_IMG	.65625	2.37486	.34278	-.03334	1.34584	1.914	47	.062	ns
Pair 9	Proudconfidentselfassured_VR - Proudconfidentselfassured_IMG	.63542	1.84719	.26662	.09905	1.17179	2.383	47	.021	.23
Pair 10	Serenecontentpeaceful_VR - Serenecontentpeaceful_IMG	1.59375	2.62690	.37916	.83098	2.35652	4.203	47	.000	.56

Paired Samples Test

		Paired Differences								
		Mean	Std. Deviation	Std. Error Mean	95% Confidence Interval of the Difference Lower	95% Confidence Interval of the Difference Upper	t	df	Sig. (2-tailed)	d'
Pair 1	Amusedfunlovingsilly_2D - Amusedfunlovingsilly_IMG	.11458	2.21513	.31973	-.52862	.75779	.358	47	.722	ns
Pair 2	Awewonderamazement_2D - Awewonderamazement_IMG	.84375	2.24830	.32451	.19091	1.49659	2.600	47	.012	.37
Pair 3	Gratefulappreciativethankful_2D - Gratefulappreciativethankful_IMG	-.92708	4.90981	.70867	-2.35274	.49858	-1.308	47	.197	ns
Pair 4	Hopefuloptimisticencouraged_2D - Hopefuloptimisticencouraged_IMG	-.08333	1.48157	.21385	-.51354	.34687	-.390	47	.699	ns
Pair 5	Inspiredupliftedelevated_2D - Inspiredupliftedelevated_IMG	-.16667	1.83446	.26478	-.69934	.36601	-.629	47	.532	ns
Pair 6	Interestedalertcurious_2D - Interestedalertcurious_IMG	.82292	2.32354	.33537	.14823	1.49760	2.454	47	.018	.31
Pair 7	Joyfulgladhappy_2D - Joyfulgladhappy_IMG	.15625	1.88543	.27214	-.39122	.70372	.574	47	.569	ns
Pair 8	Loveclosenesstrust_2D - Loveclosenesstrust_IMG	-.47917	1.98655	.28673	-1.05600	.09767	-1.671	47	.101	ns
Pair 9	Proudconfidentselfassured_2D - Proudconfidentselfassured_IMG	-.32292	1.32283	.19093	-.70703	.06119	-1.691	47	.097	ns
Pair 10	Serenecontentpeaceful_2D - Serenecontentpeaceful_IMG	-.36458	2.24968	.32471	-1.01782	.28866	-1.123	47	.267	ns

Study 2 MANOVA of Negative Affect Ratings (mDES Negative)

Multivariate Tests[a]

Effect			Value	F	Hypothesis df	Error df	Sig.	Partial Eta Squared	Noncent. Parameter	Observed Power[c]
Between Subjects	Intercept	Pillai's Trace	.552	4.556[b]	10.000	37.000	.000	.552	45.562	.995
		Wilks' Lambda	.448	4.556[b]	10.000	37.000	.000	.552	45.562	.995
		Hotelling's Trace	1.231	4.556[b]	10.000	37.000	.000	.552	45.562	.995
		Roy's Largest Root	1.231	4.556[b]	10.000	37.000	.000	.552	45.562	.995
	Group	Pillai's Trace	.287	1.488[b]	10.000	37.000	.183	.287	14.883	.632
		Wilks' Lambda	.713	1.488[b]	10.000	37.000	.183	.287	14.883	.632
		Hotelling's Trace	.402	1.488[b]	10.000	37.000	.183	.287	14.883	.632
		Roy's Largest Root	.402	1.488[b]	10.000	37.000	.183	.287	14.883	.632
Within Subjects	VRorPAINTorIMG	Pillai's Trace	.520	1.460[b]	20.000	27.000	.178	.520	29.210	.725
		Wilks' Lambda	.480	1.460[b]	20.000	27.000	.178	.520	29.210	.725
		Hotelling's Trace	1.082	1.460[b]	20.000	27.000	.178	.520	29.210	.725
		Roy's Largest Root	1.082	1.460[b]	20.000	27.000	.178	.520	29.210	.725
	VRorPAINTorIMG * Group	Pillai's Trace	.594	1.977[b]	20.000	27.000	.050	.594	39.538	.875
		Wilks' Lambda	.406	1.977[b]	20.000	27.000	.050	.594	39.538	.875
		Hotelling's Trace	1.464	1.977[b]	20.000	27.000	.050	.594	39.538	.875
		Roy's Largest Root	1.464	1.977[b]	20.000	27.000	.050	.594	39.538	.875

Univariate Tests

Source	Measure		Type III Sum of Squares	df	Mean Square	F	Sig.	Partial Eta Squared	Noncent. Parameter	Observed Power[a]
Format * Order	Angryirritatedannoyed	Sphericity Assumed	1.066	2	.533	.921	.402	.020	1.843	.205
		Greenhouse-Geisser	1.066	1.731	.616	.921	.390	.020	1.595	.193
		Huynh-Feldt	1.066	1.832	.582	.921	.395	.020	1.688	.197
		Lower-bound	1.066	1.000	1.066	.921	.342	.020	.921	.156
	Ashamedhumiliateddisgraced	Sphericity Assumed	.524	2	.262	.832	.438	.018	1.664	.189
		Greenhouse-Geisser	.524	1.758	.298	.832	.425	.018	1.463	.179
		Huynh-Feldt	.524	1.863	.282	.832	.431	.018	1.550	.183
		Lower-bound	.524	1.000	.524	.832	.366	.018	.832	.145
	Contemptuousscornfuldisdainful	Sphericity Assumed	.066	2	.033	.081	.922	.002	.162	.062
		Greenhouse-Geisser	.066	1.960	.034	.081	.919	.002	.159	.062
		Huynh-Feldt	.066	2.000	.033	.081	.922	.002	.162	.062
		Lower-bound	.066	1.000	.066	.081	.777	.002	.081	.059
	Disgustdistasterevulsion	Sphericity Assumed	.483	2	.241	1.087	.342	.023	2.174	.236
		Greenhouse-Geisser	.483	1.572	.307	1.087	.330	.023	1.709	.211
		Huynh-Feldt	.483	1.653	.292	1.087	.332	.023	1.797	.216
		Lower-bound	.483	1.000	.483	1.087	.303	.023	1.087	.175

	Embarrassedselfconsciousblushing	Sphericity Assumed	1.149	2	.575	.925	.400	.020	1.850	.206
		Greenhouse-Geisser	1.149	1.756	.655	.925	.390	.020	1.624	.194
		Huynh-Feldt	1.149	1.859	.618	.925	.394	.020	1.720	.199
		Lower-bound	1.149	1.000	1.149	.925	.341	.020	.925	.156
	Guiltyrepentantblameworthy	Sphericity Assumed	.899	2	.450	.757	.472	.016	1.515	.175
		Greenhouse-Geisser	.899	1.873	.480	.757	.464	.016	1.419	.171
		Huynh-Feldt	.899	1.992	.451	.757	.471	.016	1.509	.175
		Lower-bound	.899	1.000	.899	.757	.389	.016	.757	.136
	Hatedistrustsuspicion	Sphericity Assumed	.135	2	.068	.130	.878	.003	.261	.069
		Greenhouse-Geisser	.135	1.600	.085	.130	.832	.003	.209	.068
		Huynh-Feldt	.135	1.685	.080	.130	.844	.003	.220	.068
		Lower-bound	.135	1.000	.135	.130	.720	.003	.130	.064
	Saddownheartedunhappy	Sphericity Assumed	.014	2	.007	.007	.993	.000	.014	.051
		Greenhouse-Geisser	.014	1.974	.007	.007	.992	.000	.014	.051
		Huynh-Feldt	.014	2.000	.007	.007	.993	.000	.014	.051
		Lower-bound	.014	1.000	.014	.007	.933	.000	.007	.051
	Scaredfearfulafraid	Sphericity Assumed	.014	2	.007	.022	.978	.000	.044	.053
		Greenhouse-Geisser	.014	1.527	.009	.022	.953	.000	.034	.053

		Huynh-Feldt	.014	1.603	.009	.022	.958	.000	.035	.053
		Lower-bound	.014	1.000	.014	.022	.883	.000	.022	.052
	Stressednervousoverwhelmed	Sphericity Assumed	6.003	2	3.002	2.857	.063	.058	5.713	.547
		Greenhouse-Geisser	6.003	1.771	3.390	2.857	.070	.058	5.059	.513
		Huynh-Feldt	6.003	1.877	3.199	2.857	.066	.058	5.361	.529
		Lower-bound	6.003	1.000	6.003	2.857	.098	.058	2.857	.380

Study 3 MANOVA of Satisfaction Ratings

Multivariate Tests[a]

Effect			Value	F	Hypothesis df	Error df	Sig.	Partial Eta Squared	Noncent. Parameter	Observed Power[c]
Between Subjects	Intercept	Pillai's Trace	.987	271.735[b]	10.000	37.000	.000	.987	2717.352	1.000
		Wilks' Lambda	.013	271.735[b]	10.000	37.000	.000	.987	2717.352	1.000
		Hotelling's Trace	73.442	271.735[b]	10.000	37.000	.000	.987	2717.352	1.000
		Roy's Largest Root	73.442	271.735[b]	10.000	37.000	.000	.987	2717.352	1.000
	WhatFirst	Pillai's Trace	.201	.931[b]	10.000	37.000	.516	.201	9.313	.403
		Wilks' Lambda	.799	.931[b]	10.000	37.000	.516	.201	9.313	.403
		Hotelling's Trace	.252	.931[b]	10.000	37.000	.516	.201	9.313	.403
		Roy's Largest Root	.252	.931[b]	10.000	37.000	.516	.201	9.313	.403
Within Subjects	VRorPAINTorIMG	Pillai's Trace	.849	7.617[b]	20.000	27.000	.000	.849	152.338	1.000
		Wilks' Lambda	.151	7.617[b]	20.000	27.000	.000	.849	152.338	1.000
		Hotelling's Trace	5.642	7.617[b]	20.000	27.000	.000	.849	152.338	1.000
		Roy's Largest Root	5.642	7.617[b]	20.000	27.000	.000	.849	152.338	1.000
	VRorPAINTorIMG * WhatFirst	Pillai's Trace	.398	.892[b]	20.000	27.000	.598	.398	17.847	.457
		Wilks' Lambda	.602	.892[b]	20.000	27.000	.598	.398	17.847	.457
		Hotelling's Trace	.661	.892[b]	20.000	27.000	.598	.398	17.847	.457
		Roy's Largest Root	.661	.892[b]	20.000	27.000	.598	.398	17.847	.457

Univariate Tests

Source	Measure		Type III Sum of Squares	df	Mean Square	F	Sig.	Partial Eta Squared	Noncent. Parameter	Observed Power[a]
VRorPAINTorIMG	credibleasawaytoimproveselfregulationandenhancewellbeing	Sphericity Assumed	84.875	2	42.437	42.631	.000	.481	85.261	1.000
		Greenhouse-Geisser	84.875	1.836	46.235	42.631	.000	.481	78.259	1.000
		Huynh-Feldt	84.875	1.950	43.528	42.631	.000	.481	83.125	1.000
		Lower-bound	84.875	1.000	84.875	42.631	.000	.481	42.631	1.000
	credibleasaninterventionformentalhealthproblems	Sphericity Assumed	113.389	2	56.694	57.705	.000	.556	115.410	1.000
		Greenhouse-Geisser	113.389	1.869	60.667	57.705	.000	.556	107.853	1.000
		Huynh-Feldt	113.389	1.988	57.049	57.705	.000	.556	114.694	1.000

		Lower-bound	113.389	1.000	113.389	57.705	.000	.556	57.705	1.000
	easytocomplete	Sphericity Assumed	6.681	2	3.340	4.642	.012	.092	9.285	.770
		Greenhouse-Geisser	6.681	1.524	4.383	4.642	.020	.092	7.076	.685
		Huynh-Feldt	6.681	1.600	4.175	4.642	.019	.092	7.428	.700
		Lower-bound	6.681	1.000	6.681	4.642	.036	.092	4.642	.559
	helpful	Sphericity Assumed	70.792	2	35.396	37.347	.000	.448	74.693	1.000
		Greenhouse-Geisser	70.792	1.932	36.642	37.347	.000	.448	72.152	1.000
		Huynh-Feldt	70.792	2.000	35.396	37.347	.000	.448	74.693	1.000
		Lower-bound	70.792	1.000	70.792	37.347	.000	.448	37.347	1.000
	informative	Sphericity Assumed	40.667	2	20.333	27.002	.000	.370	54.005	1.000

		Greenhouse-Geisser	40.667	1.939	20.971	27.002	.000	.370	52.363	1.000
		Huynh-Feldt	40.667	2.000	20.333	27.002	.000	.370	54.005	1.000
		Lower-bound	40.667	1.000	40.667	27.002	.000	.370	27.002	.999
	calming	Sphericity Assumed	53.389	2	26.694	27.021	.000	.370	54.042	1.000
		Greenhouse-Geisser	53.389	1.861	28.689	27.021	.000	.370	50.285	1.000
		Huynh-Feldt	53.389	1.978	26.985	27.021	.000	.370	53.459	1.000
		Lower-bound	53.389	1.000	53.389	27.021	.000	.370	27.021	.999
	enjoyable	Sphericity Assumed	90.097	2	45.049	46.538	.000	.503	93.076	1.000
		Greenhouse-Geisser	90.097	1.991	45.258	46.538	.000	.503	92.646	1.000
		Huynh-Feldt	90.097	2.000	45.049	46.538	.000	.503	93.076	1.000

		Lower-bound	90.097	1.000	90.097	46.538	.000	.503	46.538	1.000
	Iwouldrecommendthisexercisetoafriend	Sphericity Assumed	85.764	2	42.882	38.005	.000	.452	76.010	1.000
		Greenhouse-Geisser	85.764	1.992	43.050	38.005	.000	.452	75.714	1.000
		Huynh-Feldt	85.764	2.000	42.882	38.005	.000	.452	76.010	1.000
		Lower-bound	85.764	1.000	85.764	38.005	.000	.452	38.005	1.000
	Iwouldcompletethisexerciseagain	Sphericity Assumed	101.375	2	50.688	30.690	.000	.400	61.381	1.000
		Greenhouse-Geisser	101.375	1.980	51.199	30.690	.000	.400	60.767	1.000
		Huynh-Feldt	101.375	2.000	50.688	30.690	.000	.400	61.381	1.000
		Lower-bound	101.375	1.000	101.375	30.690	.000	.400	30.690	1.000
	distressing	Sphericity Assumed	1.292	2	.646	1.042	.357	.022	2.084	.227

	Greenhouse-Geisser	1.292	1.625	.795	1.042	.345	.022	1.694	.207
	Huynh-Feldt	1.292	1.713	.754	1.042	.348	.022	1.785	.212
	Lower-bound	1.292	1.000	1.292	1.042	.313	.022	1.042	.170

Paired Samples Test

		Paired Differences					t	df	Sig. (2-tailed)	d'
		Mean	Std. Deviation	Std. Error Mean	95% Confidence Interval of the Difference					
					Lower	Upper				
Pair 1	VR_credible as a way to improve self-regulation and enhance well-being – 2D_credible as a way to improve self-regulation and enhance well-being	1.688	1.170	.169	1.348	2.027	9.995	47	.000	1.61

Pair 2	VR_credible as an intervention for mental health problems associated with stressful/traumatic life events like anxiety, depression, PTSD, and dissociation – 2D_credible as an intervention for mental health problems associated with stressful/traumatic life events like anxiety, depression, PTSD, and dissociation	2.042	1.237	.179	1.682	2.401	11.435	47	.000	1.94
Pair 3	VR_easy to complete – 2D_easy to complete	-.396	.869	.125	-.648	-.144	-3.156	47	.003	.51
Pair 4	VR_helpful – 2D_helpful	1.604	1.267	.183	1.236	1.972	8.770	47	.000	1.32
Pair 5	VR_informative – 2D_informative	1.083	1.127	.163	.756	1.410	6.661	47	.000	.81
Pair 6	VR_calming – 2D_calming	1.458	1.237	.179	1.099	1.818	8.168	47	.000	1.21
Pair 7	VR_enjoyable – 2D_enjoyable	1.708	1.414	.204	1.298	2.119	8.373	47	.000	1.34

Pair 8	VR_I would recommend this exercise to a friend – 2D_I would recommend this exercise to a friend	1.771	1.448	.209	1.351	2.191	8.476	47	.000	1.21
Pair 9	VR_I would complete this exercise again – 2D_I would complete this exercise again	1.938	1.791	.259	1.417	2.458	7.494	47	.000	1.13
Pair 10	VR_distressing (e.g., made me anxious or upset) – 2D_distressing (e.g., made me anxious or upset)	.229	1.225	.177	-.126	.585	1.297	47	.201	ns

Paired Samples Test

		Paired Differences					t	df	Sig. (2-tailed)	d'
		Mean	Std. Deviation	Std. Error Mean	95% Confidence Interval of the Difference					
					Lower	Upper				
Pair 1	VR_credible as a way to improve self-regulation and enhance well-being - IMG_credible as a way to improve self-regulation and enhance well-being	1.563	1.529	.221	1.119	2.006	7.082	47	.000	1.18
Pair 2	VR_credible as an intervention for mental health problems associated with stressful/traumatic life events like anxiety, depression, PTSD, and dissociation - IMG_credible as an intervention for mental health problems associated with stressful/traumatic life events like anxiety, depression, PTSD, and dissociation	1.667	1.374	.198	1.268	2.065	8.407	47	.000	1.25

Pair 3	VR_easy to complete - IMG_easy to complete	.104	1.477	.213	-.325	.533	.489	47	.627	Ns
Pair 4	VR_helpful - IMG_helpful	1.333	1.342	.194	.944	1.723	6.883	47	.000	1.00
Pair 5	VR_informative - IMG_informative	1.167	1.294	.187	.791	1.542	6.248	47	.000	.80
Pair 6	VR_calming - IMG_calming	1.000	1.368	.198	.603	1.397	5.063	47	.000	.85
Pair 7	VR_enjoyable - IMG_enjoyable	1.646	1.422	.205	1.233	2.059	8.021	47	.000	1.27
Pair 8	VR_I would recommend this exercise to a friend - IMG_I would recommend this exercise to a friend	1.458	1.529	.221	1.014	1.902	6.607	47	.000	.99
Pair 9	VR_I would complete this exercise again – IMG_I would complete this exercise again	1.563	1.725	.249	1.062	2.063	6.276	47	.000	.94
Pair 10	VR_distressing (e.g., made me anxious or upset) - IMG_distressing (e.g., made me anxious or upset)	.146	1.238	.179	-.214	.505	.816	47	.418	ns

Paired Samples Test

		Paired Differences					t	df	Sig. (2-tailed)	d'
		Mean	Std. Deviation	Std. Error Mean	95% Confidence Interval of the Difference					
					Lower	Upper				
Pair 1	2D_credible as a way to improve self-regulation and enhance well-being - IMG_credible as a way to improve self-regulation and enhance well-being	-.125	1.496	.216	-.560	.310	-.579	47	.566	ns
Pair 2	2D_credible as an intervention for mental health problems associated with stressful/traumatic life events like anxiety, depression, PTSD, and dissociation - IMG_credible as an intervention for mental health problems associated with stressful/traumatic life events like anxiety, depression, PTSD, and dissociation	-.375	1.552	.224	-.826	.076	-1.674	47	.101	ns

Pair 3	2D_easy to complete - IMG_easy to complete	.500	1.185	.171	.156	.844	2.923	47	.005	.45
Pair 4	2D_helpful - IMG_helpful	-.271	1.484	.214	-.702	.160	-1.265	47	.212	ns
Pair 5	2D_informative - IMG_informative	.083	1.302	.188	-.295	.461	.443	47	.659	ns
Pair 6	2D_calming - IMG_calming	-.458	1.570	.227	-.914	-.002	-2.022	47	.049	.39
Pair 7	2D_enjoyable - IMG_enjoyable	-.062	1.390	.201	-.466	.341	-.312	47	.757	ns
Pair 8	2D_I would recommend this exercise to a friend - IMG_I would recommend this exercise to a friend	-.312	1.490	.215	-.745	.120	-1.453	47	.153	ns
Pair 9	2D_I would complete this exercise again - IMG_I would complete this exercise again	-.375	1.886	.272	-.923	.173	-1.377	47	.175	ns
Pair 10	2D_distressing (e.g., made me anxious or upset) - IMG_distressing (e.g., made me anxious or upset)	-.083	.794	.115	-.314	.147	-.727	47	.471	ns

Study 3 MANOVA of Positive Affect Ratings (mDES Positive)

Multivariate Tests[a]

Effect			Value	F	Hypothesis df	Error df	Sig.	Partial Eta Squared	Noncent. Parameter	Observed Power[c]
Between Subjects	Intercept	Pillai's Trace	.974	139.444[b]	10.000	37.000	.000	.974	1394.436	1.000
		Wilks' Lambda	.026	139.444[b]	10.000	37.000	.000	.974	1394.436	1.000
		Hotelling's Trace	37.687	139.444[b]	10.000	37.000	.000	.974	1394.436	1.000
		Roy's Largest Root	37.687	139.444[b]	10.000	37.000	.000	.974	1394.436	1.000
	Order	Pillai's Trace	.234	1.130[b]	10.000	37.000	.367	.234	11.298	.490
		Wilks' Lambda	.766	1.130[b]	10.000	37.000	.367	.234	11.298	.490
		Hotelling's Trace	.305	1.130[b]	10.000	37.000	.367	.234	11.298	.490
		Roy's Largest Root	.305	1.130[b]	10.000	37.000	.367	.234	11.298	.490
Within Subjects	Format	Pillai's Trace	.868	8.902[b]	20.000	27.000	.000	.868	178.031	1.000
		Wilks' Lambda	.132	8.902[b]	20.000	27.000	.000	.868	178.031	1.000
		Hotelling's Trace	6.594	8.902[b]	20.000	27.000	.000	.868	178.031	1.000
		Roy's Largest Root	6.594	8.902[b]	20.000	27.000	.000	.868	178.031	1.000
	Format * Order	Pillai's Trace	.317	.626[b]	20.000	27.000	.858	.317	12.519	.313
		Wilks' Lambda	.683	.626[b]	20.000	27.000	.858	.317	12.519	.313
		Hotelling's Trace	.464	.626[b]	20.000	27.000	.858	.317	12.519	.313
		Roy's Largest Root	.464	.626[b]	20.000	27.000	.858	.317	12.519	.313

Univariate Tests

Source	Measure		Type III Sum of Squares	df	Mean Square	F	Sig.	Partial Eta Squared	Noncent. Parameter	Observed Power[a]
Format	Amusedfunlovingsilly	Sphericity Assumed	313.431	2	156.715	60.480	.000	.568	120.960	1.000
		Greenhouse-Geisser	313.431	1.975	158.682	60.480	.000	.568	119.461	1.000
		Huynh-Feldt	313.431	2.000	156.715	60.480	.000	.568	120.960	1.000
		Lower-bound	313.431	1.000	313.431	60.480	.000	.568	60.480	1.000
	Awewonderamazement	Sphericity Assumed	572.722	2	286.361	85.506	.000	.650	171.011	1.000
		Greenhouse-Geisser	572.722	1.943	294.752	85.506	.000	.650	166.143	1.000
		Huynh-Feldt	572.722	2.000	286.361	85.506	.000	.650	171.011	1.000
		Lower-bound	572.722	1.000	572.722	85.506	.000	.650	85.506	1.000
	Gratefulappreciativethankful	Sphericity Assumed	240.292	2	120.146	34.280	.000	.427	68.560	1.000
		Greenhouse-Geisser	240.292	1.997	120.354	34.280	.000	.427	68.442	1.000
		Huynh-Feldt	240.292	2.000	120.146	34.280	.000	.427	68.560	1.000
		Lower-bound	240.292	1.000	240.292	34.280	.000	.427	34.280	1.000
	Hopefuloptimisticencouraged	Sphericity Assumed	248.597	2	124.299	35.658	.000	.437	71.317	1.000
		Greenhouse-Geisser	248.597	1.910	130.130	35.658	.000	.437	68.121	1.000
		Huynh-Feldt	248.597	2.000	124.299	35.658	.000	.437	71.317	1.000
		Lower-bound	248.597	1.000	248.597	35.658	.000	.437	35.658	1.000

	Inspiredupliftedelevated	Sphericity Assumed	381.722	2	190.861	48.566	.000	.514	97.132	1.000
		Greenhouse-Geisser	381.722	1.903	200.589	48.566	.000	.514	92.421	1.000
		Huynh-Feldt	381.722	2.000	190.861	48.566	.000	.514	97.132	1.000
		Lower-bound	381.722	1.000	381.722	48.566	.000	.514	48.566	1.000
	Interestedalertcurious	Sphericity Assumed	532.597	2	266.299	61.501	.000	.572	123.001	1.000
		Greenhouse-Geisser	532.597	1.922	277.035	61.501	.000	.572	118.235	1.000
		Huynh-Feldt	532.597	2.000	266.299	61.501	.000	.572	123.001	1.000
		Lower-bound	532.597	1.000	532.597	61.501	.000	.572	61.501	1.000
	Joyfulgladhappy	Sphericity Assumed	243.931	2	121.965	36.573	.000	.443	73.146	1.000
		Greenhouse-Geisser	243.931	1.903	128.180	36.573	.000	.443	69.599	1.000
		Huynh-Feldt	243.931	2.000	121.965	36.573	.000	.443	73.146	1.000
		Lower-bound	243.931	1.000	243.931	36.573	.000	.443	36.573	1.000
	Loveclosenesstrust	Sphericity Assumed	69.264	2	34.632	15.500	.000	.252	31.000	.999
		Greenhouse-Geisser	69.264	1.883	36.785	15.500	.000	.252	29.186	.999
		Huynh-Feldt	69.264	2.000	34.632	15.500	.000	.252	31.000	.999
		Lower-bound	69.264	1.000	69.264	15.500	.000	.252	15.500	.971
	Proudconfidentselfassured	Sphericity Assumed	46.764	2	23.382	10.816	.000	.190	21.632	.989
		Greenhouse-Geisser	46.764	1.927	24.265	10.816	.000	.190	20.844	.987

		Huynh-Feldt	46.764	2.000	23.382	10.816	.000	.190	21.632	.989
		Lower-bound	46.764	1.000	46.764	10.816	.002	.190	10.816	.896
	Serenecontentpeaceful	Sphericity Assumed	273.556	2	136.778	41.522	.000	.474	83.045	1.000
		Greenhouse-Geisser	273.556	1.841	148.620	41.522	.000	.474	76.428	1.000
		Huynh-Feldt	273.556	1.955	139.895	41.522	.000	.474	81.194	1.000
		Lower-bound	273.556	1.000	273.556	41.522	.000	.474	41.522	1.000

Paired Samples Test

		Paired Differences					t	df	Sig. (2-tailed)	d'
					95% Confidence Interval of the Difference					
		Mean	Std. Deviation	Std. Error Mean	Lower	Upper				
Pair 1	VR_Amused, fun-loving, silly – 2D_Amused, fun-loving, silly	3.292	2.250	.325	2.638	3.945	10.136	47	.000	1.56
Pair 2	VR_Awe, wonder, amazement – 2D_Awe, wonder, amazement	4.167	2.391	.345	3.472	4.861	12.074	47	.000	1.70
Pair 3	VR_Grateful, appreciative, thankful – 2D_Grateful, appreciative, thankful	2.917	2.664	.385	2.143	3.690	7.584	47	.000	1.03
Pair 4	VR_Hopeful, optimistic, encouraged – 2D_Hopeful, optimistic, encouraged	3.125	2.573	.371	2.378	3.872	8.413	47	.000	1.13
Pair 5	VR_Inspired, uplifted, elevated – 2D_Inspired, uplifted, elevated	3.708	2.475	.357	2.990	4.427	10.380	47	.000	1.38
Pair 6	VR_Interested, alert, curious – 2D_Interested, alert, curious	4.208	2.649	.382	3.439	4.978	11.005	47	.000	1.71
Pair 7	VR_Joyful, glad, happy – 2D_Joyful, glad, happy	2.938	2.319	.335	2.264	3.611	8.775	47	.000	1.16
Pair 8	VR_Love, closeness, trust – 2D_Love, closeness, trust	1.646	2.383	.344	.954	2.338	4.784	47	.000	.52
Pair 9	VR_Proud, confident, self-assured – 2D_Proud, confident, self-assured	1.375	2.199	.317	.737	2.013	4.332	47	.000	.45
Pair 10	VR_Serene, content, peaceful – 2D_Serene, content, peaceful	3.250	2.188	.316	2.615	3.885	10.291	47	.000	1.39

Paired Samples Test

		Paired Differences					t	df	Sig. (2-tailed)	d'
		Mean	Std. Deviation	Std. Error Mean	95% Confidence Interval of the Difference					
					Lower	Upper				
Pair 1	VR_Amused, fun-loving, silly - IMG_Amused, fun-loving, silly	2.938	2.374	.343	2.248	3.627	8.574	47	.000	1.23
Pair 2	VR_Awe, wonder, amazement - IMG_Awe, wonder, amazement	4.292	2.568	.371	3.546	5.037	11.579	47	.000	1.63
Pair 3	VR_Grateful, appreciative, thankful - IMG_Grateful, appreciative, thankful	2.521	2.593	.374	1.768	3.274	6.736	47	.000	.85
Pair 4	VR_Hopeful, optimistic, encouraged - IMG_ Hopeful, optimistic, encouraged	2.229	2.399	.346	1.533	2.926	6.438	47	.000	.77

Pair 5	VR_Inspired, uplifted, elevated - IMG_Inspired, uplifted, elevated	3.125	2.856	.412	2.296	3.954	7.582	47	.000	1.05
Pair 6	VR_Interested, alert, curious - IMG_Interested, alert, curious	3.938	3.097	.447	3.038	4.837	8.808	47	.000	1.33
Pair 7	VR_Joyful, glad, happy - IMG_Joyful, glad, happy	2.542	2.633	.380	1.777	3.306	6.687	47	.000	.88
Pair 8	VR_Love, closeness, trust - IMG_Love, closeness, trust	1.188	1.864	.269	.646	1.729	4.414	47	.000	.36
Pair 9	VR_Proud, confident, self-assured - IMG_Proud, confident, self-assured	.896	1.871	.270	.353	1.439	3.318	47	.002	.30
Pair 10	VR_Serene, content, peaceful - IMG_Serene, content, peaceful	2.417	2.550	.368	1.676	3.157	6.565	47	.000	.94

Paired Samples Test

		Paired Differences					t	df	Sig. (2-tailed)	d'
		Mean	Std. Deviation	Std. Error Mean	95% Confidence Interval of the Difference					
					Lower	Upper				
Pair 1	2D_Amused, fun-loving, silly - IMG_Amused, fun-loving, silly	-.354	2.198	.317	-.992	.284	-1.117	47	.270	ns
Pair 2	2D_Awe, wonder, amazement - IMG_Awe, wonder, amazement	.125	2.788	.402	-.684	.934	.311	47	.757	ns
Pair 3	2D_Grateful, appreciative, thankful - IMG_Grateful, appreciative, thankful	-.396	2.672	.386	-1.172	.380	-1.026	47	.310	ns
Pair 4	2D_Hopeful, optimistic, encouraged - IMG_Hopeful, optimistic, encouraged	-.896	2.904	.419	-1.739	-.052	-2.137	47	.038	.31
Pair 5	2D_Inspired, uplifted, elevated - IMG_Inspired, uplifted, elevated	-.583	3.009	.434	-1.457	.291	-1.343	47	.186	ns

Pair 6	2D_Interested, alert, curious - IMG_Interested, alert, curious	-.271	3.044	.439	-1.155	.613	-.616	47	.541	ns
Pair 7	2D_Joyful, glad, happy - IMG_Joyful, glad, happy	-.396	2.826	.408	-1.217	.425	-.970	47	.337	ns
Pair 8	2D_Love, closeness, trust - IMG_Love, closeness, trust	-.458	2.153	.311	-1.084	.167	-1.475	47	.147	ns
Pair 9	2D_Proud, confident, self-assured - IMG_Proud, confident, self-assured	-.479	2.114	.305	-1.093	.135	-1.571	47	.123	ns
Pair 10	2D_Serene, content, peaceful - IMG_Serene, content, peaceful	-.833	2.846	.411	-1.660	-.007	-2.029	47	.048	.33

Study 3 MANOVA of Negative Affect Ratings (mDES Negative)

Multivariate Tests[a]

Effect			Value	F	Hypothesis df	Error df	Sig.	Partial Eta Squared	Noncent. Parameter	Observed Power[c]
Between Subjects	Intercept	Pillai's Trace	.392	2.387[b]	10.000	37.000	.027	.392	23.869	.869
		Wilks' Lambda	.608	2.387[b]	10.000	37.000	.027	.392	23.869	.869
		Hotelling's Trace	.645	2.387[b]	10.000	37.000	.027	.392	23.869	.869
		Roy's Largest Root	.645	2.387[b]	10.000	37.000	.027	.392	23.869	.869
	Order	Pillai's Trace	.365	2.126[b]	10.000	37.000	.047	.365	21.260	.818
		Wilks' Lambda	.635	2.126[b]	10.000	37.000	.047	.365	21.260	.818
		Hotelling's Trace	.575	2.126[b]	10.000	37.000	.047	.365	21.260	.818
		Roy's Largest Root	.575	2.126[b]	10.000	37.000	.047	.365	21.260	.818
Within Subjects	Format	Pillai's Trace	.516	1.880[b]	17.000	30.000	.064	.516	31.956	.844
		Wilks' Lambda	.484	1.880[b]	17.000	30.000	.064	.516	31.956	.844
		Hotelling's Trace	1.065	1.880[b]	17.000	30.000	.064	.516	31.956	.844
		Roy's Largest Root	1.065	1.880[b]	17.000	30.000	.064	.516	31.956	.844
	Format * Order	Pillai's Trace	.409	1.222[b]	17.000	30.000	.307	.409	20.775	.614
		Wilks' Lambda	.591	1.222[b]	17.000	30.000	.307	.409	20.775	.614
		Hotelling's Trace	.693	1.222[b]	17.000	30.000	.307	.409	20.775	.614
		Roy's Largest Root	.693	1.222[b]	17.000	30.000	.307	.409	20.775	.614

Univariate Tests

Tests of Between-Subjects Effects

Transformed Variable: Average

Source	Measure	Type III Sum of Squares	df	Mean Square	F	Sig.	Partial Eta Squared	Noncent. Parameter	Observed Power[a]
Order	Angryirritatedannoyed	5.063	1	5.063	1.138	.292	.024	1.138	.181
	Ashamedhumiliateddisgraced	2.507	1	2.507	1.318	.257	.028	1.318	.203
	Contemptuousscornfuldisdainful	2.250	1	2.250	.330	.568	.007	.330	.087
	Disgustdistasterevulsion	2.778	1	2.778	1.550	.219	.033	1.550	.230
	Embarrassedselfconsciousblushing	5.444	1	5.444	1.109	.298	.024	1.109	.178
	Guiltyrepentantblameworthy	7.563	1	7.563	3.415	.071	.069	3.415	.440
	Hatedistrustsuspicion	2.250	1	2.250	1.081	.304	.023	1.081	.175
	Saddownheartedunhappy	6.250	1	6.250	1.915	.173	.040	1.915	.273
	Scaredfearfulafraid	13.444	1	13.444	2.655	.110	.055	2.655	.358
	Stressednervousoverwhelmed	.007	1	.007	.001	.970	.000	.001	.050
